# Network Pharmacology-Based Analysis on the Potential Biological Mechanisms of Yinzhihuang Oral Liquid in Treating Neonatal Hyperbilirubinemia

**DOI:** 10.1155/2022/1672670

**Published:** 2022-10-05

**Authors:** Tianqi Liang, Yanxiang Kong, Lijun Tang, Junbin Huang, Huabin Wang, Xiaoyi Fang, Airun Zhang, Chun Chen

**Affiliations:** ^1^Division of Hematology/Oncology, Department of Pediatrics, The Seventh Affiliated Hospital, Sun Yat-sen University, Shenzhen 518107, China; ^2^Department of Reproductive Medicine, The Seventh Affiliated Hospital, Sun Yat-sen University, Shenzhen 518107, China; ^3^Department of Neonatology, The South Hospital of Southern Medical University, Guangzhou 510515, China; ^4^Department of Neonatology, the Seventh Affiliated Hospital, Sun Yat-sen University, Shenzhen 518107, China

## Abstract

**Objective:**

Neonatal hyperbilirubinemia is caused by the excessive production of bilirubin and decreased excretion ability in the neonatal period. It leads to a concentration of blood bilirubin that exceeds a certain threshold. Yinzhihuang oral liquid (YZH) is a traditional Chinese medicine mixture used in the treatment of neonatal hyperbilirubinemia in China. This article systematically explores the pharmacological mechanisms by which YZH acts in the treatment of neonatal hyperbilirubinemia through network pharmacology at the molecular level.

**Methods:**

We adopted the method of network pharmacology, which includes active component prescreening, target gene prediction, gene enrichment analysis, and network analysis.

**Results:**

According to the network pharmacological analysis, 8 genes (STAT3, AKT1, MAPK14, JUN, TP53, MAPK3, ESR1, and RELA) may be targets of YZH in the treatment of neonatal hyperbilirubinemia. In addition, Gene Ontology (GO) and Kyoto Encyclopedia of Genes and Genomes (KEGG) enrichment analyses showed that YZH may regulate antioxidation, modulate lipid metabolism, and have anti-infective properties.

**Conclusion:**

In this study, the pharmacological action and molecular mechanisms of YZH were predicted as a whole. It was found that YZH is a promising drug for treating oxidative stress due to bilirubin, as it reduces immunosuppression and helps to eliminate virus infection.

## 1. Introduction

Neonatal jaundice is one of the most common conditions in the neonatal period. Approximately 60% of term and 80% of preterm infants develop jaundice in the first week of life, and approximately 10% of breast-fed babies are still jaundiced at the age of 1 month [1]. There are various reasons for the excessive production of bilirubin and decreased excretion ability. If the concentration of plasma bilirubin exceeds a certain threshold, it can result in neonatal hyperbilirubinemia. The average full-term newborn infant has a peak serum bilirubin concentration of 5 to 6 mg per deciliter (86 to 103 *µ*mol per liter). Serum bilirubin concentrations higher than 17 mg per deciliter in full-term infants are no longer considered physiologic. Significantly elevated serum bilirubin levels allow the entry of bilirubin into the brain, causing irreversible damage. This is termed as kernicterus [2]. Severe neonatal hyperbilirubinemia can also lead to permanent sequelae, such as deafness, cerebral palsy, dental dysplasia, mental disability, and other permanent nerve damage [3]. Therefore, the active treatment of hyperbilirubinemia is key for reducing bilirubin-induced damage. At present, therapies for this condition include phototherapy, exchange transfusion, and albumin infusion. As a noninvasive, safe, and effective method, phototherapy is widely used in the treatment of neonatal jaundice [4]. However, phototherapy has some side effects, mainly including fever, evaporative fluid loss, and circadian rhythm disorder in the short term and retinal damage and melanocytic nevi in the long term [5]. Some studies have suggested that phototherapy is associated with type 1 diabetes and possibly asthma [6]. For newborns whose bilirubin levels do not reach the phototherapy threshold and the jaundice fades slowly, safely, and effectively reducing bilirubin levels remains a challenge. In China, some traditional Chinese herbal formulae offer a relatively safe and effective choice for the treatment of neonatal hyperbilirubinemia.

Yinzhihuang oral liquid (YZH) is a compound formula of traditional Chinese medicine that is composed of *Artemisiae Scopariae Herba*, *Gardeniae Fructus, Scutellariae Radix,* and *Lonicerae Japonicae Flos*. For infants with hyperbilirubinemia in China, especially those for whom phototherapy monotherapy fails, YZH can reduce bilirubin and promote the regression of jaundice [7]. For example, a meta-analysis has shown that YZH can significantly shorten the time required for jaundice to regress [8]. Animal experiments have shown that the components of this formula may inhibit hepatocyte apoptosis and promote their regeneration. This formula may also promote the secretion and excretion of bile and prevent postoperative liver failure [9]. Nevertheless, the potential mechanism by which this formula acts in the treatment of neonatal hyperbilirubinemia is not fully understood.

Traditional Chinese medicine (TCM), especially compound formulae, has the characteristics of multicomponents, multitargets, and synergistic effects, with unclear mechanisms of action and other problems. Therefore, it is difficult to verify the potential mechanisms through traditional experimental methods, and it is not easy to establish a scientific and appropriate assessment system to evaluate efficacy [10].

Network pharmacology, which is a novel research field based on pharmacology and pharmacodynamics, generates complicated interaction networks based on target molecules, biological functions, and bioactive compounds [11]. This approach has been used to study “compound-proteins/genes-disease” pathways and is capable of describing the complexities among biological systems, drugs, and diseases from a network perspective [12]. Indeed, applying network pharmacology for the research of TCM and formulae address the problems of drug active component analysis, mechanism research, and quality control, among others, and explain the role of TCM in the human biological network as a whole [13].

In this study, a comprehensive network pharmacological method was established to explore the potential mechanism of action of YZH by molecular docking and network analysis. The aim was to determine drug action-related targets and key pathways and provide a direction for further drug research.

## 2. Materials and Methods

### 2.1. Chemical Compounds and Screening

We obtained the chemical components of YZH through the TCMSP database (https://old.tcmsp-e.com/tcmsp.php), which is a unique systems pharmacology database of Chinese herbal medicines that is used for collecting the chemical components and targets of TCM [14]. To maximize the screening of useful fully active substances, two conditions were set as criteria, oral bioavailability (OB) ≥30%, and drug-likeness (DL) ≥0.18. These are the most important indicators for evaluating the characteristics of absorption, distribution, metabolism, and excretion. Finally, a total of 115 active herbal ingredients were found to meet the conditions. These ingredients included 18 specimens of *Artemisiae Scopariae Herba*, 41 specimens of *Scutellariae Radix*, 41 specimens of *Lonicerae Japonicae Flos,* and 15 specimens of *Gardeniae Fructus* (Supplement Tables 1-S4).

### 2.2. Compound Target Screening

We downloaded targets of the above components through TCMSP. Protein sequence information and the corresponding gene names were reviewed manually and downloaded from UniProt (https://www.uniprot.org/) [15]. The targets and protein sequence information were integrated by Perl software. Finally, 83 active ingredients of TCM were included, including 32 specimens of *Scutellariae Radix*, 26 specimens of *Lonicerae Japonicae Flos*, 15 specimens of *Artemisiae Scopariae Herba,* and 10 specimens of *Gardeniae Fructus*. A total of 32 components were excluded because no corresponding protein sequence or genetic information was found (Supplement Tables S5–S8).

**Figure 1:**
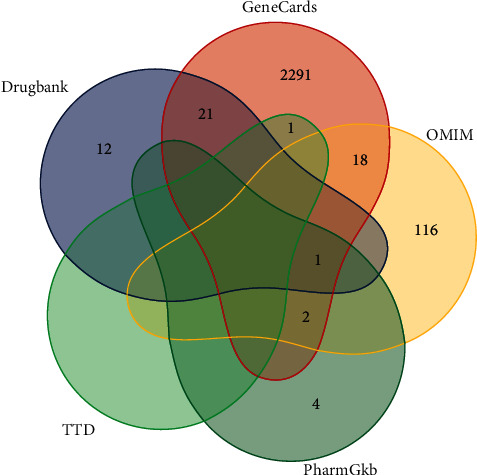
Related genes of neonatal hyperbilirubinemia via the intersection of all results from 5 databases.

Genes related to neonatal hyperbilirubinemia were searched and downloaded from public databases, including DrugBank (https://go.drugbank.com/) [16], OMIM (https://omim.org/) [17], GeneCards (https://www.genecards.org/) [18], PharmGkb (https://www.pharmgkb.org/) [19], and TTD (http://db.idrblab.net/ttd/) [20]. In total, 2466 target genes were obtained after integration and the elimination of repeats (Figure 1). To obtain candidate targets of YZH for neonatal hyperbilirubinemia, we integrated the predictive targets of the four components of YZH with the target genes of neonatal hyperbilirubinemia.

### 2.3. Protein-Protein Interaction (PPI) Data

PPI data were obtained from STRING (https://cn.string-db.org/) and used to predict protein-protein interactions [21]. Target proteins were selected with species limited to “Homo sapiens” and with a minimum required interaction score of highest confidence (0.900). Associated proteins that directly or indirectly interact with the common targets of YZH and neonatal hyperbilirubinemia were obtained through STRING.

### 2.4. Network Visualization and Acquisition of Hub Genes

Cytoscape software (version 3.9.1) was used to visually analyse the active components of YZH and the common targets of neonatal hyperbilirubinemia [22]. The nodes represent targets, compounds, and pathways, and the edges indicate interactions.

### 2.5. Gene Ontology and Pathway Analysis

Functional annotations and the involved pathways of the genes were studied by GO and KEGG enrichment analyses in R (version4.1.2 for Windows). A difference was considered to be statistically significant at *p* < 0.05.

## 3. Results

### 3.1. The Compound-CommonTarget-PPI Network between YZH and Neonatal Hyperbilirubinemia

**Figure 2:**
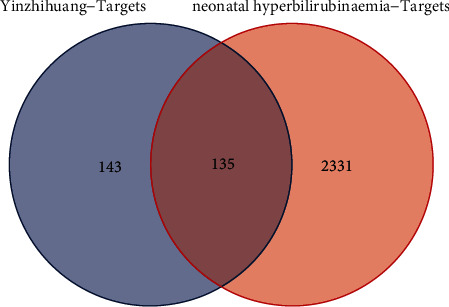
Intersection of the drug-target disease-related genes.

**Figure 3:**
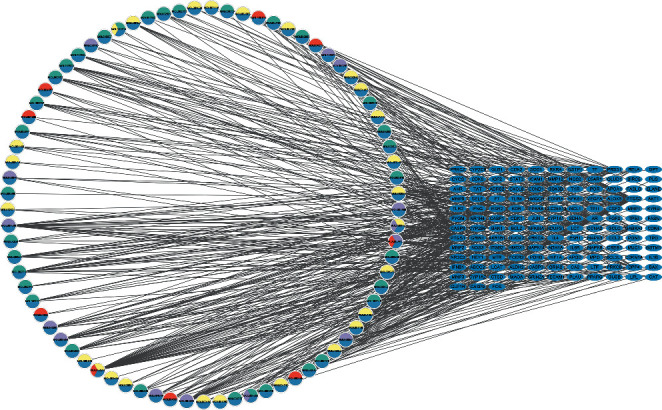
Drug-target interaction pharmacology network. Circles represent the small molecule active compounds in YZH. Each color represents a component of YZH. The ellipses represent the related target genes of neonatal hyperbilirubinemia, and the edges represent the interaction between the small molecule compounds and target genes.

**Figure 4:**
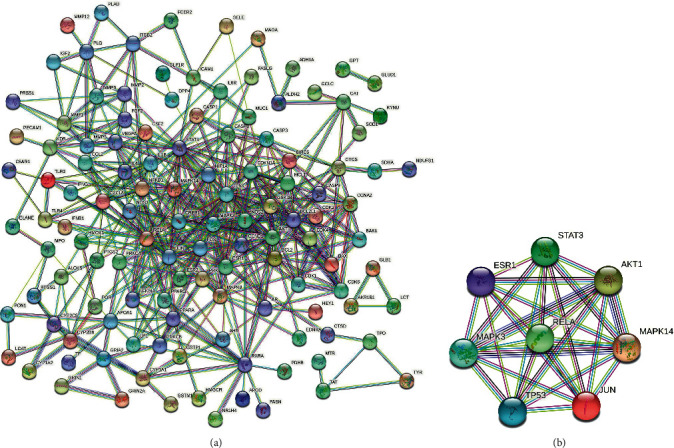
PPI network of the target proteins. (a) A PPI network for YZH and neonatal hyperbilirubinemia was exported from the STRING database. (b) A key subnetwork of 8 hub genes was analyzed by CytoNCA. The network nodes represent proteins, and the edges represent protein-protein associations.

We intersected the target genes of the drug and disease, and a total of 135 common genes were obtained (Figure 2). To further identify the targets of the drug components, we established a compound-common target network. This showed that these 135 genes correspond to 77 components in YZH (Figure 3) (Supplement Table S9). PPI network analysis was also applied to evaluate the candidate targets and interaction proteins of YZH in the treatment of neonatal hyperbilirubinemia. When setting the minimum required interaction score of 0.9, 123 genes were obtained. We imported the PPI network into Cytoscape for further analysis, including 123 nodes and 894 edges (Supplement Table S10). Furthermore, we analyzed the PPI network again in Cytoscape software, and finally, 8 hub genes forming an interactive network were obtained by using CytoNCA : STAT3, AKT1, MAPK14, JUN, TP53, MAPK3, ESR1, and RELA. These data suggest that YZH may act on neonatal hyperbilirubinemia mainly through the network of STAT3-AKT1-MAPK14-JUN-TP53-MAPK3-ESR1-RELA (Figure 4).

### 3.2. GO Enrichment Analysis

**Figure 5:**
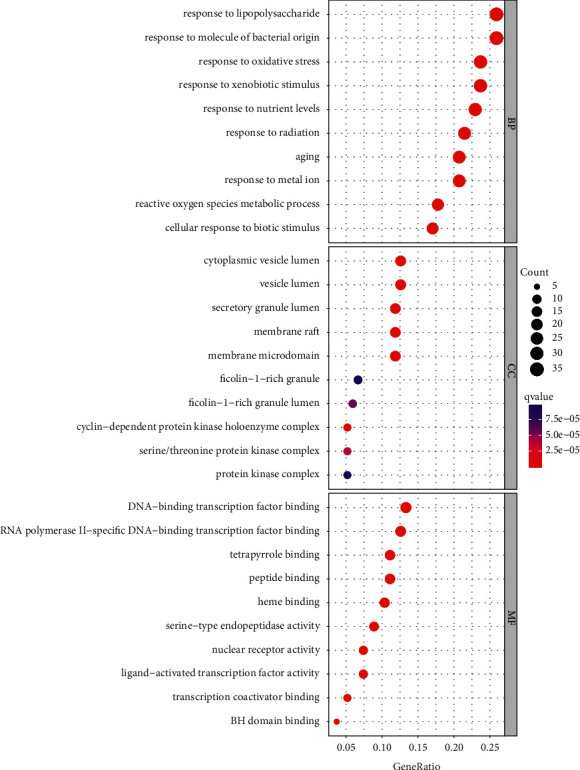
GO analysis of the target genes. The gene ratio refers to the ratio of enriched genes to all target genes, and counts refer to the numbers of enriched genes. BP: biological process; CC: cell component; MF: molecular function.

GO enrichment analysis was used to reveal the biological process (BP), cellular component (CC), and molecular function (MF) terms of the 123 target genes. By setting the filter as an adjusted q value <0.05 and *p* value <0.05, we obtained 2356 significantly enriched GO terms. The results of the GO analysis showed that YZH treats neonatal hyperbilirubinemia through a variety of biological processes. The top 5 processes were the response to lipopolysaccharide (GO:0032496), response to molecule of bacterial origin (GO:0002237), response to oxidative stress (GO:0006979), response to xenobiotic stimulus (GO:0009410), and response to nutrient levels (GO:0031667). The main CC terms were cytoplasmic vesicle lumen (GO:0060205), vesicle lumen (GO:0031983), secretory granule lumen (GO:0034774), membrane raft (GO:0045121), and membrane microdomain (GO:0098857). The top five MF terms were DNA-binding transcription factor binding (GO:0140297), RNA polymerase II-specific DNA-binding transcription factor binding (GO:0061629), tetrapyrrole binding (GO:0046906), peptide binding (GO:0042277), and heme binding (GO:0020037) (Figure 5) (Supplement Table S11). In conclusion, these target genes have an essential role in antibacterial origin and response to oxidative stress.

### 3.3. KEGG Pathway Enrichment Analysis

**Figure 6:**
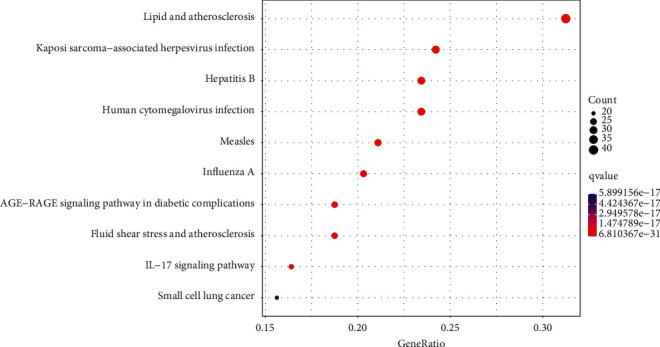
KEGG enrichment analysis of the target genes. The gene ratio refers to the ratio of enriched genes to all target genes, and counts refer to the numbers of enriched genes.

KEGG pathway enrichment analysis was performed to discover the pathway enrichment of the 123 target genes with an adjusted q value <0.05 and a *p* value <0.05. Overall, these target genes are involved in the pathways of lipid metabolism (hsa05417), hepatitis B virus infection (hsa05161), Kaposi sarcoma-associated herpesvirus infection (hsa05167), measles (hsa05162), and human cytomegalovirus infection (hsa05163). In summary, the related pathways are mainly involved in lipid metabolism and virus infection, including hepatitis B virus and human cytomegalovirus (Figure 6) (Supplement Table S12).

## 4. Discussion

Neonatal hyperbilirubinemia is a condition of yellow coloring of the skin, sclera, and mucosa caused by abnormal bilirubin metabolism in infants. Severe hyperbilirubinemia can lead to bilirubin encephalopathy and permanent damage to the central nervous system [3], resulting in serious harm to children, their family, and society. Therefore, the rapid, safe, and effective treatment of neonatal hyperbilirubinemia is key to reducing damage. In terms of treatment, phototherapy is a noninvasive treatment that has been widely used in the treatment of neonatal jaundice. However, phototherapy is associated with a series of short-term and long-term side effects [23], and how to shorten the time of phototherapy and improve the effect remain challenges. In China, TCM plays a significant role in the treatment of neonatal hyperbilirubinemia, especially in combination with phototherapy [7]. Through the study of network pharmacology, we found that YZH has 77 components targeting 123 genes related to neonatal hyperbilirubinemia. PPI network analysis revealed 8 hub targets of these 123 genes. GO and KEGG analyses showed that YZH might play a role in antioxidation, the regulation of lipid metabolism and anti-infection.

YZH is mainly composed of four components:A*rtemisiae Scopariae Herba, Scutellariae Radix, Lonicerae Japonicae Flos,* and *Gardeniae Fructus*. *Artemisiae Scopariae Herba* is an ancient TCM that is mainly used to treat “damp-heat style (Shi Re Zheng)” jaundice, hepatitis, heatstroke, and allergic inflammatory dermatitis. Randomized controlled clinical trials have reported that this drug preparation can significantly shorten the recovery time of serum total bilirubin and regression time of jaundice in the treatment of neonatal jaundice [24]. In TCM, *Scutellariae Radix* is the root of *Scutellaria baicalensis Georgi* (Lamiaceae). The small molecule components extracted from it have antiviral, antitumor, antibacterial, antioxidation, and anti-inflammation activities and protect hepatocytes and nerve cells [25]. Animal experiments show that *Scutellaria baicalensis Georgi* may treat lipopolysaccharide-induced liver injury in mice by inhibiting the cytokines TNF-*α*, IL-1*β*, IL-6, COX-2, iNOS, and NF-kB [26]. *Lonicerae Japonicae Flos* has been widely used in China as an edible herbal medicine. Its extract has anti-inflammatory, bacteriostatic, antiviral, antioxidant, and hepatoprotective effects [27]. The newly discovered components of monoterpenoids, japopenoid A, japopenoid B, japopenoid C, and caffeoylquinic acid derivative from its extract have anti-hepatoma and anti-HBV activities [28]. *Gardenia jasminoides* is another TCM, and the preparation can be used to treat acute or chronic hepatic diseases, icteric hepatitis, itching skin, eczema, diabetes, and depression [29]. Animal experiments also found that *Gardeniae Fructus* can reduce thioacetamide-induced liver fibrosis in mice through the AMPK/SIRT1/NF-*κ*B and Nrf2 signal pathways [30]. The analysis of the effects of the components of YZH indicate that YZH has certain effects on the treatment of jaundice and has liver protective, anti-inflammatory, and antioxidative properties.

Bilirubin is an effective antioxidant, and low levels of bilirubin have antioxidant effects. However, when the blood bilirubin level exceeds a certain threshold, it can cause oxidative stress and lead to DNA damage [31]. Bilirubin-induced DNA damage activates poly (ADP-ribose) polymerase (PARP), a DNA damage repair enzyme involved in DNA repair [32]. PARP activates NF-*κ*B signaling to regulate the inflammatory response [33]. RELA is a key protein of the NF-*κ*B transcription factor family and the core of DNA damage response pathway. It serves as a “master regulator” of the cellular inflammatory and stress responses. The RELA protein network can be phosphorylated to different degrees according to the type of DNA damage. Thus, it can affect different pathways to regulate DNA damage repair [34]. Nonetheless, unconjugated bilirubin can prevent NF-*κ*B protein production by inhibiting RELA phosphorylation [35]. In our PPI network analysis, we found that the RELA gene was one of the key nodes of the hub gene subnetwork, docking between small molecules and proteins. GO and KEGG enrichment analyses also showed enrichment in the function of the antioxidant response. The above results suggest that YZH plays a role in repairing DNA damage caused by excess bilirubin during the treatment of neonatal hyperbilirubinemia.

Bilirubin has been shown to possess potent immunomodulatory properties through the innate and adaptive immune systems [35]. It inhibits immune cells in vivo and in vitro by affecting cell migration, adhesion, proliferation, and infiltration [36]. In newborns, bilirubin can inhibit inflammation and increase the production of antioxidant enzymes in neutrophils [37]. Moreover, it was found that a certain concentration of bilirubin induces neutrophil apoptosis, resulting in the downregulation of the blood IL-8 level [38]. CD4+ T cells are crucial in the whole-body resistance to HAV infection, and they produce a large number of cytokines in the early stages of infection, including IFN-*γ*, TNF-*α*, IL-2, and IL-21. However, it has been reported that high levels of BR induce apoptosis in reactive CD4+ T cells [39]. The finding that the absence of an effective CD4+ T-cell response during HCV infection results in the development of an exhausted CD8+ T-cell pool has been attributed to chronic antigen-specific stimulation [40]. Furthermore, an increase in conjugated bilirubin in patients with chronic HBV infection can inhibit the activity of mucosal-associated invariant T cells and the expression of cytokines. This results in the decline of anti-infection ability [41]. These findings suggest that bilirubin may inhibit the activity of T cells and affect the severity of hepatitis virus infection. In our PPI network analysis, we found that one of the targets of YZH is AKT1, which plays an essential role in immune cell modulation. The kinase AKT, also known as protein kinase B, is a serine/threonine-specific protein kinase that is mainly involved in the PI3K-AKT-mammalian target of rapamycin (mTOR) pathway. It regulates the development and functions of innate immune cells, including neutrophils and macrophages [42]. In addition, Akt is a key signaling node in the development of protective memory CD8+ T-cell responses [43]. Akt can also mediate the early metabolic response of naive human CD4+ T cells to TCR stimulation [44]. Hence, YZH may reduce the immunosuppressive effect of bilirubin.

## 5. Conclusions

In summary, we studied the potential therapeutic mechanism by which YZH acts in the treatment of neonatal hyperbilirubinemia by means of network pharmacology. The results showed that YZH could improve the oxidative stress and immunosuppression caused by excess bilirubin in neonates with hyperbilirubinemia. It was also found that this drug targets the treatment of hepatitis virus infection, offering a new strategy for the treatment of hyperbilirubinemia caused by hepatitis virus infection. However, the mechanisms of the active components of YZH still need to be clarified through further experiments.

## Data Availability

The datasets used and/or analyzed during the current study are available from the corresponding author upon reasonable request.

## Supplementary Materials

Supplementary Materials Table S1: active herbal ingredients in Scutellariae Radix. Table S2: active herbal ingredients in Lonicerae Japonicae Flos. Table S3: active herbal ingredients in Artemisiae Scopariae Herba. Table S4: active herbal ingredients in Gardeniae Fructus. Table S5: ingredients in Scutellariae Radix and corresponding targets. Table S6: ingredients in Lonicerae Japonicae Flos and corresponding targets. Table S7: ingredients in Artemisiae Scopariae Herba and corresponding targets. Table S8: ingredients in Gardeniae Fructus and corresponding targets. Table S9: compound-common target network of YZH and neonatal hyperbilirubinemia. Table S10: PPI network into Cytoscape for YZH and neonatal hyperbilirubinemia analysis (minimum required interaction score of 0.9). Table S11: Gene Ontology (GO) Biological Process analysis (*p* < 0.05).
