# Supplementary material for: Network Pharmacology-Based Analysis on the Potential Biological Mechanisms of Yinzhihuang Oral Liquid in Treating Neonatal Hyperbilirubinemia
Source: Evid Based Complement Alternat Med. 2022 Oct 5;2022:1672670. doi: 10.1155/2022/1672670 (PMC9556251; doi:10.1155/2022/1672670)
Supplement: Supplementary Materials — Table S1: active herbal ingredients in Scutellariae Radix. Table S2: active herbal ingredients in Lonicerae Japonicae Flos. Table S3: active herbal ingredients in Artemisiae Scopariae Herba. Table S4: active herbal ingredients in Gardeniae Fructus. Table S5: ingredients in Scutellariae Radix and corresponding targets. Table S6: ingredients in Lonicerae Japonicae Flos and corresponding targets. Table S7: ingredients in Artemisiae Scopariae Herba and corresponding targets. Table S8: ingredients in Gardeniae Fructus and corresponding targets. Table S9: compound-common target network of YZH and neonatal hyperbilirubinemia. Table S10: PPI network into Cytoscape for YZH and neonatal hyperbilirubinemia analysis (minimum required interaction score of 0.9). Table S11: Gene Ontology (GO) Biological Process analysis (p < 0.05). [file 1672670.f1.zip › Table S1.pdf]

Table S1 Active Herbal Ingredients in Scutellariae Radix

Ingredients	MOL_ID	Molecule_Name	OB（%）	DL
Scutellariae Radix	MOL001300	PEL	86.98412518	0.78574
Scutellariae Radix	MOL000018	(+/-)-Isoborneol	76.25704989	0.77132
Scutellariae Radix	MOL000198	(R)-linalool	74.2352200131	0.75665
Scutellariae Radix	MOL000219	BOX	73.0751978707	0.75304
Scutellariae Radix	MOL002573	β-patchoulene	69.5104339827	0.75123
Scutellariae Radix	MOL002714	baicalein	69.0398755728	0.7512
Scutellariae Radix	MOL002737	scutellarein	67.6036274848	0.74097
Scutellariae Radix	MOL002908	-Trihydroxy-7-methoxyfla	66.0617387242	0.72244
Scutellariae Radix	MOL002909	etrahydroxy-8,6-dimethox	64.5416405	0.71577
Scutellariae Radix	MOL002910	Carthamidin	61.1969813041	0.6241
Scutellariae Radix	MOL002911	-tetrahydroxy-6'-methoxyc	58.6232646438	0.59239
Scutellariae Radix	MOL002912	Dihydrobaicalin	57.8975251428	0.5919
Scutellariae Radix	MOL002913	Dihydrobaicalin_qt	57.3971065901	0.47958
Scutellariae Radix	MOL002914	Eriodyctiol (flavanone)	56.2419020882	0.44739
Scutellariae Radix	MOL002915	Salvigenin	55.56099313	0.43917
Scutellariae Radix	MOL002916	oxyphenyl)-3,5,7-trihydro	55.2331738887	0.4379
Scutellariae Radix	MOL002917	Trihydroxy-7,8-dimethoxy	51.5964711696	0.40911
Scutellariae Radix	MOL002918	Ganhuangenin	50.6885654128	0.37374
Scutellariae Radix	MOL002922	ıydroxyethyl)-2-methoxyp	49.0659260554	0.35462
Scutellariae Radix	MOL002923	darendoside B	49.0614071247	0.33279
Scutellariae Radix	MOL002924	darendoside B_qt	48.95984114	0.33057
Scutellariae Radix	MOL002925	7,2',6'-Tetrahydroxyflavoı	48.192560362	0.2915
Scutellariae Radix	MOL002926	dihydrooroxylin A	46.1530068079	0.28551
Scutellariae Radix	MOL002927	Skullcapflavone II	45.9896389415	0.27314
Scutellariae Radix	MOL002928	oroxylin a	45.0474280192	0.26666
Scutellariae Radix	MOL002929	salidroside	44.0879595947	0.26546
Scutellariae Radix	MOL002930	Tyrosol	44.03418878	0.24387
Scutellariae Radix	MOL002931	scutellarin	43.9814962531	0.24382
Scutellariae Radix	MOL002932	Panicolin	43.8298515785	0.2436
Scutellariae Radix	MOL002933	-Trihydroxy-8-methoxyfla	43.5933254731	0.24189
Scutellariae Radix	MOL002934	NEOBAICALEIN	43.5933254731	0.24162
Scutellariae Radix	MOL002935	Baicalin	43.2902406442	0.23233

Scutellariae Radix	MOL002936	ihydroxy-6,7-dimethoxyfl	43.0923322819	0.23057
Scutellariae Radix	MOL002937	DIHYDROOROXYLIN	41.9343581437	0.22987
Scutellariae Radix	MOL000357	Sitogluside	41.9044360216	0.22151
Scutellariae Radix	MOL000358	beta-sitosterol	41.3675690034	0.21994
Scutellariae Radix	MOL000359	sitosterol	41.3504271334	0.21306
Scutellariae Radix	MOL000396	(+)-Syringaresinol	41.150962728	0.20888
Scutellariae Radix	MOL000458	campesterol	40.5088579998	0.20723
Scutellariae Radix	MOL000525	Norwogonin	40.107907069	0.20722
Scutellariae Radix	MOL000552	hydroxy-6,7,8-trimethoxy	40.0377810256	0.19818
