# Supplementary material for: Network Pharmacology-Based Analysis on the Potential Biological Mechanisms of Yinzhihuang Oral Liquid in Treating Neonatal Hyperbilirubinemia
Source: Evid Based Complement Alternat Med. 2022 Oct 5;2022:1672670. doi: 10.1155/2022/1672670 (PMC9556251; doi:10.1155/2022/1672670)
Supplement: Supplementary Materials — Table S1: active herbal ingredients in Scutellariae Radix. Table S2: active herbal ingredients in Lonicerae Japonicae Flos. Table S3: active herbal ingredients in Artemisiae Scopariae Herba. Table S4: active herbal ingredients in Gardeniae Fructus. Table S5: ingredients in Scutellariae Radix and corresponding targets. Table S6: ingredients in Lonicerae Japonicae Flos and corresponding targets. Table S7: ingredients in Artemisiae Scopariae Herba and corresponding targets. Table S8: ingredients in Gardeniae Fructus and corresponding targets. Table S9: compound-common target network of YZH and neonatal hyperbilirubinemia. Table S10: PPI network into Cytoscape for YZH and neonatal hyperbilirubinemia analysis (minimum required interaction score of 0.9). Table S11: Gene Ontology (GO) Biological Process analysis (p < 0.05). [file 1672670.f1.zip › Table S10.pdf]

Table S10 PPI network into Cytoscape for YZH and neonatal hyperbilirubinemia analysis（minimum required interaction score of 0.9）

Name	Betweenness	Closeness	Degree	Eigenvector	LAC	Network
ADH1A	0	0.104095563	2	1.65E-04	0	0
ALDH2	252.5784533	0.115530303	6	0.00239526	0	0
AHR	100.9518176	0.129787234	8	0.03859706	1	1.1428571
AR	331.3188792	0.135105205	22	0.1103409	5.0909091	5.5659148
RELA	1555.525666	0.141860465	60	0.25453129	14	36.471141
CYP1A1	450.5570738	0.125514403	14	0.01903496	1.1428571	1.2867133
ESR1	1280.55388	0.138321995	32	0.18422635	12.75	16.069107
AKR1B1	0	0.008264463	4	7.02E-09	2	2.6666667
LCT	0	0.008264463	4	1.86E-09	2	2.6666667
GLB1	0	0.008264463	4	3.27E-10	2	2.6666667
AKT1	1326.663799	0.14071511	48	0.22679091	12.833333	23.364199
NFKB1	568.2991382	0.136008919	30	0.1291368	8.5333333	12.307956
MAPK14	508.6207314	0.138794084	38	0.21080317	13.684211	18.288771
CAT	856.2763513	0.12721585	14	0.02909858	1.1428571	2.2564103
MAPK3	1321.278088	0.141367323	56	0.2637991	14.428571	28.864039
STAT3	2297.414678	0.141695703	62	0.25754917	13.032258	32.30379
TP53	1499.842539	0.140068886	60	0.26009527	13.6	33.362738
SOD1	0	0.125256674	4	0.01741905	2	2.6666667
NOS3	78.18886708	0.131891892	16	0.08674853	7.5	8.5333333
BCL2L1	97.48482702	0.133333333	26	0.12618749	12.615385	16.881488
CASP3	75.41739452	0.132464712	22	0.11014795	9.8181818	11.900661
EDNRA	0.869902028	0.125385406	4	0.02599888	0	0
GSK3B	87.75500796	0.133187773	20	0.10242763	7.6	8.4901484
NOS2	377.393916	0.136465324	20	0.11218274	8	8.5182186
CASP9	18.04482274	0.128421053	12	0.05958186	6	6.5454545
MCL1	34.53036121	0.13174946	20	0.10248188	10.4	11.948473
JUN	1521.884042	0.142025611	66	0.29523099	15.757576	42.237567
PRKCZ	44.46838584	0.131465517	16	0.07668788	6.5	7.2242424
BCL2	257.8085913	0.136465324	26	0.13905411	12	14.889599
CDKN1A	194.3380015	0.135405105	32	0.16174744	12.25	17.548291
CREB1	149.1854108	0.136312849	32	0.18748777	14.25	18.010278
RXRA	1499.17918	0.135405105	36	0.10331543	4.2222222	8.6110999
HIF1A	57.25724989	0.136924804	28	0.18437071	15.714286	17.466023
MAOA	85.9592775	0.117874396	4	0.00598679	0	0
ALOX5	113.1813393	0.122613065	10	0.01062216	4	4.6984127
IL4	444.8681535	0.130760986	22	0.08568827	8	11.095992
CYP2C9	159.7336645	0.121878122	12	0.00645452	4.6666667	6.7186147
CYP2B6	233.9820378	0.12286002	14	0.00965914	4	6.5507826
PTGS1	42.59599567	0.113488372	8	0.00211304	3	3.4285714
PTGS2	546.5814665	0.130901288	14	0.052455	3.4285714	4.0777001
APOA1	901.2756857	0.131182796	18	0.05560596	4.4444444	7.7828368
PON1	5.898290598	0.118102614	6	0.00436202	2.6666667	3.7333333
MPO	134.3581809	0.119960669	8	0.00441783	1	1.3714286
PPARA	266.2846406	0.133918771	20	0.08889118	8.4	10.77359

TF	0	0.116634799	2	0.00378726	0	0
LPL	15.16935842	0.130481283	12	0.06010969	7.3333333	8
LCAT	0	0.116746411	4	0.00408267	2	2.6666667
APOD	0	0.119960669	2	0.00701349	0	0
CCND1	194.4759267	0.135555556	32	0.17160803	14	19.023924
CDK6	14.2981203	0.125128205	16	0.05484736	9	9.7641026
HEY1	0	0.119725221	2	0.00749214	0	0
CASP8	193.7018926	0.133918771	22	0.10240261	6.5454545	8.0601504
MAPK8	375.0394708	0.135857461	32	0.15494661	7.5	10.432449
BAK1	10.24896138	0.127748691	12	0.05128951	8.6666667	9.4545455
BAX	17.67210636	0.129511677	14	0.06117847	9.1428571	10.125874
CYCS	560.8242741	0.129100529	20	0.064487	6.4	7.9470004
BIRC5	51.44691149	0.130760986	16	0.0839764	6	6.4820513
CCNA2	5.53604153	0.127083333	14	0.05228982	9.1428571	9.8461538
FOS	196.9467071	0.136312849	34	0.18017364	12.235294	18.016681
CDK1	111.9384489	0.129787234	18	0.06977619	9.7777778	11.414781
C5AR1	0	0.11380597	4	0.00533039	2	2.6666667
TLR2	112.3836039	0.127348643	10	0.03750816	5.6	7.1111111
TLR4	123.6878013	0.127481714	12	0.04084364	5.3333333	7.3189033
CASP1	3.586067527	0.127348643	8	0.04047513	4	4.5714286
IL1B	283.2645015	0.134657837	30	0.12231314	10.133333	17.083364
FASLG	2.349897292	0.126293996	4	0.02448439	0	0
GCLC	0	0.113488372	2	0.00197872	0	0
KYNU	0	0.113488372	2	0.00197872	0	0
CCL2	7.387301587	0.129100529	14	0.0750296	8.5714286	9.3986014
CSF2	24.32581396	0.131182796	12	0.06182371	6	6.5454545
CXCL8	56.52801821	0.133333333	20	0.104932	10.8	12.661023
CDK4	5.824136768	0.12721585	16	0.06439188	11	11.938462
CDK2	26.80027058	0.129237288	16	0.07467684	10	10.871795
HMOX1	8.542930637	0.128016789	12	0.06859099	7.3333333	8.2424242
POR	104.4888248	0.127348643	10	0.05033438	4.8	5.3333333
VEGFA	210.787398	0.132177681	24	0.09887991	9.6666667	13.22219
CTSD	904	0.123232323	4	0.01258087	0	0
TPO	684	0.110909091	6	8.63E-04	1.3333333	2.1333333
SDHA	232	0.115203022	4	0.00442234	0	0
GSTM1	0	0.112132353	2	0.0012976	0	0
GSTP1	34.61538927	0.128827878	6	0.03190072	1.3333333	1.6
CYP1A2	0	0.110108303	4	0.00109357	2	2.6666667
DPP4	344.6634793	0.126687435	6	0.01849225	0	0
PLG	371.0454343	0.120792079	16	0.01065135	3	4.8820513
GLP1R	0	0.113067655	2	0.00125788	0	0
ELANE	61.22493328	0.117988395	4	0.00282415	0	0
MMP1	469.6958469	0.129237288	20	0.03725937	8	12.224117
ESR2	2.450043378	0.130481283	10	0.06864068	6.4	7.1111111
MUC1	223.6321554	0.130202775	14	0.05964866	4	6.028638
FASN	0	0.119960669	2	0.00701349	0	0

FCER2	0	0.115859449	4	0.00289112	2	2.6666667
ICAM1	232.7330243	0.127083333	12	0.02607866	3.3333333	5.991342
ITGB2	341.7342045	0.124872057	14	0.01648058	2.2857143	4.5687646
FGF2	113.4406628	0.131182796	16	0.05431981	8	8.8752137
MMP2	125.1192229	0.127748691	14	0.03449536	6.8571429	7.8769231
KDR	234.3076923	0.124872057	10	0.03076566	2.4	2.6666667
MMP3	305.2836367	0.130481283	16	0.03889803	7	8.5333333
MMP9	178.3460887	0.128964059	18	0.03777618	8.8888889	11.487179
PPARG	51.67045107	0.132608696	14	0.08712481	5.1428571	5.7062937
IFNB1	8.757395412	0.127615063	8	0.05234212	4	4.5714286
GLUD1	0	0.008196721	2	0	0	0
GPT	0	0.008196721	2	0	0	0
GRIA2	460.9667702	0.119607843	8	0.00885993	1	2.6666667
PRKCB	262.9663657	0.129925453	12	0.06050516	5.3333333	5.8181818
GRIN2A	0	0.107488987	4	6.46E-04	2	2.6666667
GRIN1	0	0.107488987	4	6.46E-04	2	2.6666667
PRKCA	561.0288354	0.13304253	16	0.06836786	5.5	5.8666667
HMGCR	0	0.121878122	4	0.01306001	2	2.6666667
SELE	0	0.116634799	4	0.00582368	2	2.6666667
IL6R	22.54713074	0.129649309	8	0.04127857	4	4.5714286
IFNG	0	0.128556375	8	0.05146489	6	6.8571429
IGF2	2	0.116190476	6	0.00558473	2.6666667	3.2
PLAU	0	0.113172542	4	0.00184177	2	2.6666667
NFKBIA	130.1106644	0.136160714	24	0.13074118	10.666667	12.863907
PECAM1	0	0.111619396	2	0.00208823	0	0
PRSS1	0	0.117195005	6	0.00772265	4	4.8
MMP12	0	0.108348135	2	7.21E-04	0	0
MTR	0	0.091798345	2	4.28E-06	0	0
TAT	232	0.100577082	6	6.32E-05	1.3333333	2.1333333
NDUFS1	0	0.103829787	2	3.02E-04	0	0
NR1H4	0	0.119960669	2	0.00701349	0	0
PDHB	0	0.119960669	2	0.00701349	0	0
TYR	0	0.100494234	4	6.29E-05	2	2.6666667
