# Supplementary material for: Network Pharmacology-Based Analysis on the Potential Biological Mechanisms of Yinzhihuang Oral Liquid in Treating Neonatal Hyperbilirubinemia
Source: Evid Based Complement Alternat Med. 2022 Oct 5;2022:1672670. doi: 10.1155/2022/1672670 (PMC9556251; doi:10.1155/2022/1672670)
Supplement: Supplementary Materials — Table S1: active herbal ingredients in Scutellariae Radix. Table S2: active herbal ingredients in Lonicerae Japonicae Flos. Table S3: active herbal ingredients in Artemisiae Scopariae Herba. Table S4: active herbal ingredients in Gardeniae Fructus. Table S5: ingredients in Scutellariae Radix and corresponding targets. Table S6: ingredients in Lonicerae Japonicae Flos and corresponding targets. Table S7: ingredients in Artemisiae Scopariae Herba and corresponding targets. Table S8: ingredients in Gardeniae Fructus and corresponding targets. Table S9: compound-common target network of YZH and neonatal hyperbilirubinemia. Table S10: PPI network into Cytoscape for YZH and neonatal hyperbilirubinemia analysis (minimum required interaction score of 0.9). Table S11: Gene Ontology (GO) Biological Process analysis (p < 0.05). [file 1672670.f1.zip › Table S11.pdf]

Table S11 Gene Ontology (GO) Biological Process analysis (p < 0.05)

ONTOLOGY	ID	Description	GeneRatio	BgRatio	pvalue	p.adjust	qvalue	geneID	Count
BP	GO:0032496	response to lipopolysaccharide	35/135	343/18723	1.51E-30	5.62E-27	2.53E-27	PTGS2/RELA/AKT1/FOS/CASP3/MPO/NOS2/CYP1A2/MAPK14/CASP9/JUN/CASP8/PRKCA/MAPK3/CDK4/MAPK8/NFKBIA/ICAM1/IL1B/SELE/CSF2/NOS3/FASLG/CASP1/ELANE/TLR4/TLR2/GSTP1/NFKB1/LTF/CCL2/CXCL8/CYP1A1/ABCC2/C5AR1	35
BP	GO:0002237	response to molecule of bacterial origin	35/135	363/18723	1.09E-29	2.04E-26	9.15E-27	PTGS2/RELA/AKT1/FOS/CASP3/MPO/NOS2/CYP1A2/MAPK14/CASP9/JUN/CASP8/PRKCA/MAPK3/CDK4/MAPK8/NFKBIA/ICAM1/IL1B/SELE/CSF2/NOS3/FASLG/CASP1/ELANE/TLR4/TLR2/GSTP1/NFKB1/LTF/CCL2/CXCL8/CYP1A1/ABCC2/C5AR1	35
BP	GO:0006979	response to oxidative stress	32/135	446/18723	4.17E-23	5.18E-20	2.33E-20	PTGS2/PTGS1/RELA/AKT1/BCL2/FOS/MMP9/CASP3/TP53/HIF1A/CDK1/MPO/APOD/JUN/MAPK3/BAK1/MMP2/MAPK8/MMP3/MCL1/DUOX2/NOS3/GCLC/TLR4/ALOX5/SOD1/CAT/GSTP1/TPO/TAT/ABCC2/HMOX1	32
BP	GO:0009410	response to xenobiotic stimulus	32/135	462/18723	1.23E-22	1.15E-19	5.15E-20	PTGS2/RELA/BCL2/FOS/CASP3/CDK1/AHR/APOD/NOS2/CYP1A2/CYP2C9/JUN/BAK1/CYP2B6/STAT3/CDKN1A/ICAM1/IL1B/CREB1/GCLC/LPL/SOD1/CAT/GSTP1/APOA1/GRIN1/GRIN2A/CYP1A1/ABCC2/POR/HMOX1/GSTM1	32
BP	GO:0031667	response to nutrient levels	31/135	474/18723	3.41E-21	2.11E-18	9.51E-19	PTGS2/RELA/AKT1/BCL2/TP53/MPO/ADRB2/F7/PPARG/JUN/PON1/CCND1/MAPK3/HMGCR/CDKN1A/MAPK8/ICAM1/GCLC/LPL/PPARA/NR1H4/SOD1/CAT/GSTP1/APOA1/KYNU/GPT/TYR/CYP1A1/POR/HMOX1	31
BP	GO:0009314	response to radiation	29/135	456/18723	1.66E-19	6.88E-17	3.09E-17	PTGS2/RELA/AKT1/BCL2/FOS/BAX/MMP9/CASP3/TP53/HIF1A/BCL2L1/MAPK14/CASP9/JUN/CCND1/BAK1/HMGCR/CDKN1A/MMP2/MAPK8/MMP1/MMP3/ICAM1/CREB1/ELANE/CAT/GRIN1/GRIN2A/TYR	29
BP	GO:0007568	aging	28/135	339/18723	6.81E-22	5.08E-19	2.28E-19	PTGS2/RELA/AKT1/BCL2/FOS/TP53/CDK1/MPO/APOD/CDK2/MAPK14/CASP9/JUN/MAPK3/BAK1/HMGCR/STAT3/CDKN1A/CDK6/MAPK8/FGF2/ICAM1/CREB1/GCLC/SOD1/CAT/KYNU/CYP1A1	28
BP	GO:0010038	response to metal ion	28/135	373/18723	9.10E-21	4.24E-18	1.90E-18	PTGS2/AKT1/BCL2/FOS/MMP9/CASP3/HIF1A/CDK1/CYP1A2/CASP9/JUN/CASP8/CCND1/MAPK3/CDK4/MAPK8/ICAM1/CREB1/GCLC/SOD1/CAT/TAT/TF/SLC25A13/CYP1A1/ABCC2/CA2/HMOX1	28
BP	GO:0072593	reactive oxygen species metabolic process	24/135	239/18723	7.71E-21	4.10E-18	1.84E-18	AKT1/BCL2/TP53/HIF1A/MPO/NOS2/CYP1A2/MAPK14/STAT3/CDKN1A/MMP3/DUOX2/NOS3/TLR4/PPARA/ALOX5/SOD1/CAT/GSTP1/TPO/GRIN1/NDUFS1/CYP1A1/ITGB2	24
BP	GO:0071216	cellular response to biotic stimulus	23/135	246/18723	2.71E-19	1.01E-16	4.55E-17	RELA/AKT1/TP53/NOS2/GSK3B/MAPK14/PRKCA/MAPK3/CDK4/MAPK8/NFKBIA/ICAM1/IL1B/CSF2/CASP1/TLR4/TLR2/GSTP1/NFKB1/LTF/CCL2/CXCL8/ABCC2	23
CC	GO:0060205	cytoplasmic vesicle lumen	17/135	325/19550	1.02E-10	1.41E-08	1.04E-08	VEGFA/MPO/IGF2/MAPK14/FASLG/ELANE/ALOX5/CAT/GSTP1/APOA1/PLG/NFKB1/GUSB/CTSD/LTF/GLB1/TF	17
CC	GO:0031983	vesicle lumen	17/135	327/19550	1.13E-10	1.41E-08	1.04E-08	VEGFA/MPO/IGF2/MAPK14/FASLG/ELANE/ALOX5/CAT/GSTP1/APOA1/PLG/NFKB1/GUSB/CTSD/LTF/GLB1/TF	17
CC	GO:0034774	secretory granule lumen	16/135	322/19550	7.97E-10	6.64E-08	4.89E-08	VEGFA/MPO/IGF2/MAPK14/ELANE/ALOX5/CAT/GSTP1/APOA1/PLG/NFKB1/GUSB/CTSD/LTF/GLB1/TF	16
CC	GO:0045121	membrane raft	16/135	335/19550	1.41E-09	7.06E-08	5.20E-08	PTGS2/KDR/DPP4/CASP3/CASP8/MAPK3/ICAM1/SELE/PECAM1/NOS3/FASLG/TLR2/CTSD/ITGB2/HMOX1/PRKCZ	16
CC	GO:0098857	membrane microdomain	16/135	335/19550	1.41E-09	7.06E-08	5.20E-08	PTGS2/KDR/DPP4/CASP3/CASP8/MAPK3/ICAM1/SELE/PECAM1/NOS3/FASLG/TLR2/CTSD/ITGB2/HMOX1/PRKCZ	16
CC	GO:0101002	ficolin-1-rich granule	9/135	185/19550	5.62E-06	0.0001336	9.84E-05	MMP9/MAPK14/ALOX5/CAT/GSTP1/GUSB/CTSD/GLB1/ITGB2	9
CC	GO:1904813	ficolin-1-rich granule lumen	8/135	124/19550	2.36E-06	7.37E-05	5.43E-05	MMP9/MAPK14/ALOX5/CAT/GSTP1/GUSB/CTSD/GLB1	8
CC	GO:0000307	in-dependent protein kinase holoenzyme comp	7/135	42/19550	1.41E-08	5.88E-07	4.34E-07	CDK1/CDK2/CCND1/CDK4/CDKN1A/CDK6/CCNA2	7
CC	GO:1902554	serine/threonine protein kinase complex	7/135	81/19550	1.46E-06	5.20E-05	3.83E-05	CDK1/CDK2/CCND1/CDK4/CDKN1A/CDK6/CCNA2	7
CC	GO:1902911	protein kinase complex	7/135	96/19550	4.58E-06	0.0001272	9.37E-05	CDK1/CDK2/CCND1/CDK4/CDKN1A/CDK6/CCNA2	7
MF	GO:0140297	DNA-binding transcription factor binding	18/135	394/18368	6.72E-10	5.10E-08	3.25E-08	RXRA/AR/RELA/BCL2/FOS/TP53/HIF1A/PPARG/ESR1/GSK3B/MAPK14/JUN/PRKCB/STAT3/NFKBIA/CREB1/PPARA/NR1H4	18
MF	GO:0061629	erase II-specific DNA-binding transcription fa	17/135	299/18368	7.21E-11	6.56E-09	4.19E-09	RXRA/AR/RELA/FOS/TP53/HIF1A/PPARG/ESR1/GSK3B/MAPK14/JUN/PRKCB/STAT3/NFKBIA/CREB1/PPARA/NR1H4	17
MF	GO:0046906	tetrapyrrole binding	15/135	149/18368	2.83E-13	1.29E-10	8.23E-11	PTGS2/PTGS1/MPO/CYCS/NOS2/CYP1A2/CYP2C9/CYP2B6/DUOX2/NOS3/CAT/TPO/MTR/CYP1A1/HMOX1	15
MF	GO:0042277	peptide binding	15/135	321/18368	1.45E-08	6.62E-07	4.22E-07	RXRA/RELA/ADRB2/PPARG/NFKBIA/GRIA2/TLR4/TLR2/CAT/APOA1/GRIN1/GRIN2A/ITGB2/GLP1R/GSTM1	15
MF	GO:0020037	heme binding	14/135	139/18368	1.82E-12	4.13E-10	2.64E-10	PTGS2/PTGS1/MPO/CYCS/NOS2/CYP1A2/CYP2C9/CYP2B6/DUOX2/NOS3/CAT/TPO/CYP1A1/HMOX1	14
MF	GO:0004252	serine-type endopeptidase activity	12/135	174/18368	6.01E-09	3.42E-07	2.18E-07	DPP4/PRSS1/MMP9/F7/PLAU/MMP2/MMP1/MMP3/ELANE/PLG/LTF/MMP12	12
MF	GO:0004879	nuclear receptor activity	10/135	52/18368	3.99E-12	4.54E-10	2.90E-10	RXRA/AR/AHR/PPARG/ESR2/ESR1/NR3C2/STAT3/PPARA/NR1H4	10
MF	GO:0098531	ligand-activated transcription factor activity	10/135	52/18368	3.99E-12	4.54E-10	2.90E-10	RXRA/AR/AHR/PPARG/ESR2/ESR1/NR3C2/STAT3/PPARA/NR1H4	10
MF	GO:0001223	transcription coactivator binding	7/135	32/18368	2.86E-09	1.86E-07	1.19E-07	AR/RELA/HIF1A/AHR/ESR1/CREB1/PPARA	7
MF	GO:0051400	BH domain binding	5/135	11/18368	8.88E-09	4.49E-07	2.87E-07	BCL2/BAX/BCL2L1/BAK1/MCL1	5
