# Supplementary material for: Network Pharmacology-Based Analysis on the Potential Biological Mechanisms of Yinzhihuang Oral Liquid in Treating Neonatal Hyperbilirubinemia
Source: Evid Based Complement Alternat Med. 2022 Oct 5;2022:1672670. doi: 10.1155/2022/1672670 (PMC9556251; doi:10.1155/2022/1672670)
Supplement: Supplementary Materials — Table S1: active herbal ingredients in Scutellariae Radix. Table S2: active herbal ingredients in Lonicerae Japonicae Flos. Table S3: active herbal ingredients in Artemisiae Scopariae Herba. Table S4: active herbal ingredients in Gardeniae Fructus. Table S5: ingredients in Scutellariae Radix and corresponding targets. Table S6: ingredients in Lonicerae Japonicae Flos and corresponding targets. Table S7: ingredients in Artemisiae Scopariae Herba and corresponding targets. Table S8: ingredients in Gardeniae Fructus and corresponding targets. Table S9: compound-common target network of YZH and neonatal hyperbilirubinemia. Table S10: PPI network into Cytoscape for YZH and neonatal hyperbilirubinemia analysis (minimum required interaction score of 0.9). Table S11: Gene Ontology (GO) Biological Process analysis (p < 0.05). [file 1672670.f1.zip › Table S12.pdf]

Table S11 Kyoto Encyclopedia of Genes and Genomes (KEGG) Pathway analysis (p < 0.05)

ID	Description	q-value	p-value	geneID	Count
hsa05417	Lipid and atherosclerosis	6. 81E-31	7.44E-33	A/RELA/AKT1/BCL2/FOS/BAX/MMP9/CASP3/TP53/CYCS/PPARG/CYP2C9/BCL2L1/GSK3B/MAPK14/CASP9/JUN/CASP8/PRKCA/MAPK3/CYP2B6/STAT3/MAPK8/NFKBIA/MMP1/MMP3/ICAM1/IL1B/SELE/NOS3/FASLG/CASP1/TLR4/TLR2/APOA1/NFKB1/IFNB1/CCL2/CXCL8/CY	40
hsa05161	Hepatitis B	8. 99E-23	1.96E-24	RELA/AKT1/BCL2/FOS/BAX/MMP9/CASP3/TP53/CYCS/CDK2/MAPK14/CASP9/JUN/CASP8/PRKCA/MAPK3/PRKCB/STAT3/CDKN1A/MAPK8/NFKBIA/CREB1/BIRC5/FASLG/TLR4/TLR2/NFKB1/IFNB1/CXCL8/CCNA2	30
hsa05167	Kaposi sarcoma-associated herpesvirus infection	9. 91E-22	3.25E-23	PTGS2/RELA/AKT1/VEGFA/FOS/BAX/CASP3/TP53/HIF1A/CYCS/GSK3B/MAPK14/CASP9/JUN/CASP8/CCND1/MAPK3/CDK4/BAK1/STAT3/CDKN1A/CDK6/MAPK8/NFKBIA/FGF2/ICAM1/CREB1/CSF2/NFKB1/IFNB1/CXCL8	31
hsa05162	Measles	3. 14E-21	1.37E-22	RELA/AKT1/BCL2/FOS/BAX/CASP3/TP53/CYCS/CDK2/BCL2L1/GSK3B/CASP9/JUN/CASP8/CCND1/CDK4/BAK1/STAT3/CDK6/MAPK8/NFKBIA/IL1B/FASLG/TLR4/TLR2/NFKB1/IFNB1	27
hsa04933	AGE-RAGE signaling pathway in diabetic complications	3. 30E-21	1.80E-22	RELA/AKT1/VEGFA/BCL2/BAX/CASP3/MAPK14/JUN/PRKCA/CCND1/MAPK3/CDK4/PRKCB/STAT3/MMP2/MAPK8/ICAM1/IL1B/SELE/NOS3/NFKB1/CCL2/CXCL8/PRKCZ	24
hsa05163	Human cytomegalovirus infection	6. 21E-19	4.07E-20	PTGS2/RELA/AKT1/VEGFA/BAX/CASP3/TP53/CYCS/IL6R/GSK3B/MAPK14/CASP9/CASP8/PRKCA/CCND1/MAPK3/CDK4/BAK1/PRKCB/STAT3/CDKN1A/CDK6/NFKBIA/IL1B/CREB1/FASLG/NFKB1/IFNB1/CCL2/CXCL8	30
hsa04657	IL-17 signaling pathway	6. 37E-18	4.87E-19	PTGS2/RELA/FOS/MMP9/CASP3/GSK3B/MAPK14/JUN/CASP8/MAPK3/MAPK8/NFKBIA/MMP1/MMP3/IL1B/CSF2/IFNG/IL4/NFKB1/CCL2/CXCL8	21
hsa05164	Influenza A	7. 00E-18	6.12E-19	PRSS1/RELA/AKT1/BAX/CASP3/CYCS/CASP9/CASP8/PRKCA/MAPK3/CDK4/BAK1/PRKCB/CDK6/NFKBIA/ICAM1/IL1B/FASLG/CASP1/IFNG/TLR4/PLG/NFKB1/IFNB1/CCL2/CXCL8	26
hsa05418	Fluid shear stress and atherosclerosis	7. 47E-18	7.34E-19	KDR/RELA/AKT1/VEGFA/BCL2/FOS/MMP9/TP53/MAPK14/JUN/MMP2/MAPK8/ICAM1/IL1B/SELE/PECAM1/NOS3/IFNG/GSTP1/NFKB1/CCL2/HMOX1/GSTM1/PRKCZ	24
hsa05222	Small cell lung cancer	5. 90E-17	6.44E-18	PTGS2/RXRA/RELA/AKT1/BCL2/BAX/CASP3/TP53/CYCS/NOS2/CDK2/BCL2L1/CASP9/CCND1/CDK4/BAK1/CDKN1A/CDK6/NFKBIA/NFKB1	20
