# Supplementary material for: Network Pharmacology-Based Analysis on the Potential Biological Mechanisms of Yinzhihuang Oral Liquid in Treating Neonatal Hyperbilirubinemia
Source: Evid Based Complement Alternat Med. 2022 Oct 5;2022:1672670. doi: 10.1155/2022/1672670 (PMC9556251; doi:10.1155/2022/1672670)
Supplement: Supplementary Materials — Table S1: active herbal ingredients in Scutellariae Radix. Table S2: active herbal ingredients in Lonicerae Japonicae Flos. Table S3: active herbal ingredients in Artemisiae Scopariae Herba. Table S4: active herbal ingredients in Gardeniae Fructus. Table S5: ingredients in Scutellariae Radix and corresponding targets. Table S6: ingredients in Lonicerae Japonicae Flos and corresponding targets. Table S7: ingredients in Artemisiae Scopariae Herba and corresponding targets. Table S8: ingredients in Gardeniae Fructus and corresponding targets. Table S9: compound-common target network of YZH and neonatal hyperbilirubinemia. Table S10: PPI network into Cytoscape for YZH and neonatal hyperbilirubinemia analysis (minimum required interaction score of 0.9). Table S11: Gene Ontology (GO) Biological Process analysis (p < 0.05). [file 1672670.f1.zip › Table S2.pdf]

Table S2 Active Herbal Ingredients in Lonicerae Japonicae Flos

Ingredients	MOL_ID	Molecule_Name	OB（%）	DL
Lonicerae Japonicae Flos	MOL000116	Nonanal	89.3819859741	0.9383
Lonicerae Japonicae Flos	MOL001168	-4-isopropylidene-1-meth	89.2791403739	0.79511
Lonicerae Japonicae Flos	MOL001180	γ-muurolene	87.4721900226	0.78674
Lonicerae Japonicae Flos	MOL000123	geraniol	85.1157636606	0.78042
Lonicerae Japonicae Flos	MOL000125	(-)-alpha-Pinene	83.5449108643	0.77998
Lonicerae Japonicae Flos	MOL001283	C09704	81.2287930026	0.76905
Lonicerae Japonicae Flos	MOL001300	PEL	79.2136454153	0.75647
Lonicerae Japonicae Flos	MOL001388	(+)-Ledol	76.1570301876	0.75599
Lonicerae Japonicae Flos	MOL001392	Methyl myristate	73.1391653591	0.75457
Lonicerae Japonicae Flos	MOL001393	myristic acid	72.2736519589	0.7433
Lonicerae Japonicae Flos	MOL001410	Zeaxanthin	68.6831175958	0.74312
Lonicerae Japonicae Flos	MOL000511	ursolic acid	68.0800710003	0.73616
Lonicerae Japonicae Flos	MOL000149	inositol	67.4419315387	0.72618
Lonicerae Japonicae Flos	MOL001494	Mandenol	67.1738128547	0.68525
Lonicerae Japonicae Flos	MOL001495	Ethyl linolenate	66.4048805775	0.66762
Lonicerae Japonicae Flos	MOL001600	copaene	65.5209219822	0.62617
Lonicerae Japonicae Flos	MOL001604	Linalool	62.7422371601	0.58358
Lonicerae Japonicae Flos	MOL001620	Pentadecene	61.1857094418	0.5731
Lonicerae Japonicae Flos	MOL000163	Atractylodin	60.8905644579	0.56922
Lonicerae Japonicae Flos	MOL001640	NON	60.01364431	0.55114
Lonicerae Japonicae Flos	MOL001719	thenyl-5-methyloxolan-2-	59.5305643932	0.54186
Lonicerae Japonicae Flos	MOL000172	Furol	59.3294059663	0.50316
Lonicerae Japonicae Flos	MOL001773	indole	57.4658718451	0.45063
Lonicerae Japonicae Flos	MOL001837	Methyl-p-coumarate	56.2419020882	0.44911
Lonicerae Japonicae Flos	MOL001875	isochlorogenic,acid	55.9242283041	0.43365
Lonicerae Japonicae Flos	MOL000019	D-Camphene	55.79046742	0.42613
Lonicerae Japonicae Flos	MOL000196	L-Bornyl acetate	55.7070293811	0.36155
Lonicerae Japonicae Flos	MOL000551	Hederagenol	55.6365254379	0.35727
Lonicerae Japonicae Flos	MOL001980	Helixin	55.4791757285	0.35505
Lonicerae Japonicae Flos	MOL001982	disacoside B_qt	55.0731604766	0.33891
Lonicerae Japonicae Flos	MOL002042	thymol	54.9705395881	0.32964
Lonicerae Japonicae Flos	MOL000009	luteolin-7-o-glucoside	53.6463279259	0.32642

Lonicerae Japonicae Flos	MOL002083	tricin	53.6166900595	0.30132
Lonicerae Japonicae Flos	MOL002085	alpha-Cubebene	53.4613596861	0.28548
Lonicerae Japonicae Flos	MOL002121	1,11-tetramethylbicyclo[8	51.9565134525	0.27415
Lonicerae Japonicae Flos	MOL001955	Heriguard	51.16818355	0.25976
Lonicerae Japonicae Flos	MOL002229	HEPTACOSANE	50.2041941021	0.2436
Lonicerae Japonicae Flos	MOL000252	farnesol	49.3673709995	0.23288
Lonicerae Japonicae Flos	MOL002522	beta-Rhodinol	48.8731127145	0.19716
Lonicerae Japonicae Flos	MOL000254	eugenol	48.46185122	0.19321
Lonicerae Japonicae Flos	MOL002566	3-O-Methylquercetin	47.2476268843	0.182
