# Supplementary material for: Network Pharmacology-Based Analysis on the Potential Biological Mechanisms of Yinzhihuang Oral Liquid in Treating Neonatal Hyperbilirubinemia
Source: Evid Based Complement Alternat Med. 2022 Oct 5;2022:1672670. doi: 10.1155/2022/1672670 (PMC9556251; doi:10.1155/2022/1672670)
Supplement: Supplementary Materials — Table S1: active herbal ingredients in Scutellariae Radix. Table S2: active herbal ingredients in Lonicerae Japonicae Flos. Table S3: active herbal ingredients in Artemisiae Scopariae Herba. Table S4: active herbal ingredients in Gardeniae Fructus. Table S5: ingredients in Scutellariae Radix and corresponding targets. Table S6: ingredients in Lonicerae Japonicae Flos and corresponding targets. Table S7: ingredients in Artemisiae Scopariae Herba and corresponding targets. Table S8: ingredients in Gardeniae Fructus and corresponding targets. Table S9: compound-common target network of YZH and neonatal hyperbilirubinemia. Table S10: PPI network into Cytoscape for YZH and neonatal hyperbilirubinemia analysis (minimum required interaction score of 0.9). Table S11: Gene Ontology (GO) Biological Process analysis (p < 0.05). [file 1672670.f1.zip › Table S3.pdf]

Table S3 Active Herbal Ingredients in Artemisiae Scopariae Herba

Ingredients	MOL_ID	Molecule_Name	OB（%）	DL
Artemisiae Scopariae Herba	MOL000118	(L)-alpha-Terpineol	87.0101665582	0.76904
Artemisiae Scopariae Herba	MOL001314	Azelex	74.7549576865	0.75123
Artemisiae Scopariae Herba	MOL000172	Furol	73.3601144144	0.68283
Artemisiae Scopariae Herba	MOL001801	salicylic acid	72.1821809631	0.41394
Artemisiae Scopariae Herba	MOL001880	OXL	68.6578184608	0.41385
Artemisiae Scopariae Herba	MOL001955	Heriguard	68.3171908193	0.37367
Artemisiae Scopariae Herba	MOL001999	scoparone	62.0183203488	0.36988
Artemisiae Scopariae Herba	MOL000207	Methyleugenol	59.9887758946	0.35197
Artemisiae Scopariae Herba	MOL000251	Rhamnocitrin	57.5559199642	0.34347
Artemisiae Scopariae Herba	MOL000254	eugenol	57.4037150566	0.32797
Artemisiae Scopariae Herba	MOL002818	Piceol	56.2419020882	0.32642
Artemisiae Scopariae Herba	MOL000339	Isoscopoletin	52.4396760331	0.31388
Artemisiae Scopariae Herba	MOL000354	isorhamnetin	52.3323214051	0.306
Artemisiae Scopariae Herba	MOL000358	beta-sitosterol	51.9960077723	0.26607
Artemisiae Scopariae Herba	MOL000040	Scopoletol	49.604377053	0.26434
Artemisiae Scopariae Herba	MOL000415	rutin	48.9643507174	0.24932
Artemisiae Scopariae Herba	MOL000437	Hirsutrin	48.7977727286	0.23696
Artemisiae Scopariae Herba	MOL004609	Areapillin	47.19217569	0.23516
