# Supplementary material for: Network Pharmacology-Based Analysis on the Potential Biological Mechanisms of Yinzhihuang Oral Liquid in Treating Neonatal Hyperbilirubinemia
Source: Evid Based Complement Alternat Med. 2022 Oct 5;2022:1672670. doi: 10.1155/2022/1672670 (PMC9556251; doi:10.1155/2022/1672670)
Supplement: Supplementary Materials — Table S1: active herbal ingredients in Scutellariae Radix. Table S2: active herbal ingredients in Lonicerae Japonicae Flos. Table S3: active herbal ingredients in Artemisiae Scopariae Herba. Table S4: active herbal ingredients in Gardeniae Fructus. Table S5: ingredients in Scutellariae Radix and corresponding targets. Table S6: ingredients in Lonicerae Japonicae Flos and corresponding targets. Table S7: ingredients in Artemisiae Scopariae Herba and corresponding targets. Table S8: ingredients in Gardeniae Fructus and corresponding targets. Table S9: compound-common target network of YZH and neonatal hyperbilirubinemia. Table S10: PPI network into Cytoscape for YZH and neonatal hyperbilirubinemia analysis (minimum required interaction score of 0.9). Table S11: Gene Ontology (GO) Biological Process analysis (p < 0.05). [file 1672670.f1.zip › Table S4.pdf]

Table S4 Active Herbal Ingredients in Gardeniae Fructus

Ingredients	MOL_ID	Molecule_Name	OB（%）	DL
Gardeniae Fructus	MOL001955	Heriguard	84.0659257749	0.75713
Gardeniae Fructus	MOL001663	12a-heptamethyl-1,3,4,5,6,	73.0751978707	0.75665
Gardeniae Fructus	MOL004552	Isoimperatorin	62.4640299666	0.75599
Gardeniae Fructus	MOL004553	Gardenone	60.1630714796	0.75457
Gardeniae Fructus	MOL004557	geniposide	55.7070293811	0.7433
Gardeniae Fructus	MOL000415	rutin	54.5883540952	0.73518
Gardeniae Fructus	MOL004559	droxy-6-(hydroxymethyl)	53.136139693	0.72269
Gardeniae Fructus	MOL004560	SHANZHISIDE_qt	52.7691380119	0.68283
Gardeniae Fructus	MOL004561	Sudan III	51.9565134525	0.59097
Gardeniae Fructus	MOL000511	ursolic acid	51.0281707395	0.44151
Gardeniae Fructus	MOL000551	Hederagenol	50.34658294	0.43958
Gardeniae Fructus	MOL000003	MTL	46.4333481195	0.39372
Gardeniae Fructus	MOL000023	Hemo-sol	45.9896389415	0.32642
Gardeniae Fructus	MOL000035	beta-Selinene	45.5774475544	0.24066
Gardeniae Fructus	MOL000105	protocatechuic acid	45.4642467387	0.21626
