# Supplementary material for: Network Pharmacology-Based Analysis on the Potential Biological Mechanisms of Yinzhihuang Oral Liquid in Treating Neonatal Hyperbilirubinemia
Source: Evid Based Complement Alternat Med. 2022 Oct 5;2022:1672670. doi: 10.1155/2022/1672670 (PMC9556251; doi:10.1155/2022/1672670)
Supplement: Supplementary Materials — Table S1: active herbal ingredients in Scutellariae Radix. Table S2: active herbal ingredients in Lonicerae Japonicae Flos. Table S3: active herbal ingredients in Artemisiae Scopariae Herba. Table S4: active herbal ingredients in Gardeniae Fructus. Table S5: ingredients in Scutellariae Radix and corresponding targets. Table S6: ingredients in Lonicerae Japonicae Flos and corresponding targets. Table S7: ingredients in Artemisiae Scopariae Herba and corresponding targets. Table S8: ingredients in Gardeniae Fructus and corresponding targets. Table S9: compound-common target network of YZH and neonatal hyperbilirubinemia. Table S10: PPI network into Cytoscape for YZH and neonatal hyperbilirubinemia analysis (minimum required interaction score of 0.9). Table S11: Gene Ontology (GO) Biological Process analysis (p < 0.05). [file 1672670.f1.zip › Table S5.pdf]

Table S5 Ingredients in Scutellariae Radix and corresponding targets

Ingredients	MOL_ID	Molecule_Name	Protein name	Gene Name	Uniprot
Scutellariae Radix	MOL001300	PEL	Alcohol dehydrogenase 1B	ADH1B	P00325
Scutellariae Radix	MOL001300	PEL	Alcohol dehydrogenase 1C	ADH1C	P00326
Scutellariae Radix	MOL001300	PEL	Trypsin-3	PRSS3	P35030
Scutellariae Radix	MOL001300	PEL	Alcohol dehydrogenase 1A	ADH1A	P07327
Scutellariae Radix	MOL001300	PEL	Amine oxidase [flavin-containing] A	MAOA	P21397
Scutellariae Radix	MOL000018	(+/-)-Isoborneol	Muscarinic acetylcholine receptor M2	CHRM2	P08172
Scutellariae Radix	MOL000018	(+/-)-Isoborneol	Gamma-aminobutyric acid receptor subunit alpha-1	GABRA1	P14867
Scutellariae Radix	MOL000018	(+/-)-Isoborneol	Muscarinic acetylcholine receptor M3	CHRM3	P20309
Scutellariae Radix	MOL000018	(+/-)-Isoborneol	Muscarinic acetylcholine receptor M1	CHRM1	P11229
Scutellariae Radix	MOL000018	(+/-)-Isoborneol	Prostaglandin G/H synthase 2	PTGS2	P35354
Scutellariae Radix	MOL000018	(+/-)-Isoborneol	Sodium-dependent noradrenaline transporter	SLC6A2	P23975
Scutellariae Radix	MOL000018	(+/-)-Isoborneol	Alpha-1A adrenergic receptor	ADRA1A	P35348
Scutellariae Radix	MOL000018	(+/-)-Isoborneol	Progesterone receptor	PGR	P06401
Scutellariae Radix	MOL000198	(R)-linalool	Muscarinic acetylcholine receptor M1	CHRM1	P11229
Scutellariae Radix	MOL000198	(R)-linalool	Muscarinic acetylcholine receptor M2	CHRM2	P08172
Scutellariae Radix	MOL000198	(R)-linalool	Prostaglandin G/H synthase 1	PTGS1	P23219
Scutellariae Radix	MOL000198	(R)-linalool	Potassium voltage-gated channel subfamily H member 2	KCNH2	Q12809
Scutellariae Radix	MOL000198	(R)-linalool	Sodium channel protein type 5 subunit alpha	SCN5A	Q14524
Scutellariae Radix	MOL000198	(R)-linalool	Prostaglandin G/H synthase 2	PTGS2	P35354
Scutellariae Radix	MOL000198	(R)-linalool	Vascular endothelial growth factor receptor 2	KDR	P35968
Scutellariae Radix	MOL000198	(R)-linalool	Heat shock protein HSP 90	HSP90AB1	P08238
Scutellariae Radix	MOL000198	(R)-linalool	Nuclear receptor coactivator 2	NCOA2	Q15596
Scutellariae Radix	MOL000198	(R)-linalool	Muscarinic acetylcholine receptor M3	CHRM3	P20309
Scutellariae Radix	MOL000198	(R)-linalool	Gamma-aminobutyric acid receptor subunit alpha-1	GABRA1	P14867
Scutellariae Radix	MOL000198	(R)-linalool	Sodium-dependent noradrenaline transporter	SLC6A2	P23975
Scutellariae Radix	MOL000198	(R)-linalool	Progesterone receptor	PGR	P06401
Scutellariae Radix	MOL000219	BOX	Amine oxidase [flavin-containing] B	MAOB	P27338
Scutellariae Radix	MOL000219	BOX	Alcohol dehydrogenase 1B	ADH1B	P00325
Scutellariae Radix	MOL000219	BOX	Alcohol dehydrogenase 1C	ADH1C	P00326
Scutellariae Radix	MOL000219	BOX	Alcohol dehydrogenase 1A	ADH1A	P07327
Scutellariae Radix	MOL000219	BOX	Lysozyme	LYZ	P61626
Scutellariae Radix	MOL000219	BOX	Group IIE secretory phospholipase A2	PLA2G2E	Q9NZK7
Scutellariae Radix	MOL000219	BOX	Trypsin-3	PRSS3	P35030
Scutellariae Radix	MOL000219	BOX	D-amino-acid oxidase	DAO	P14920
Scutellariae Radix	MOL000219	BOX	Muscarinic acetylcholine receptor M3	CHRM3	P20309
Scutellariae Radix	MOL000219	BOX	Prostaglandin G/H synthase 2	PTGS2	P35354
Scutellariae Radix	MOL000219	BOX	Retinoic acid receptor RXR-alpha	RXRA	P19793

Scutellariae Radix	MOL000219	BOX	Gamma-aminobutyric acid receptor subunit alpha-1	GABRA1	P14867
Scutellariae Radix	MOL000219	BOX	Nuclear receptor coactivator 2	NCOA2	Q15596
Scutellariae Radix	MOL002573	β-patchoulene	Muscarinic acetylcholine receptor M3	CHRM3	P20309
Scutellariae Radix	MOL002573	β-patchoulene	Prostaglandin G/H synthase 2	PTGS2	P35354
Scutellariae Radix	MOL002573	β-patchoulene	Gamma-aminobutyric acid receptor subunit alpha-1	GABRA1	P14867
Scutellariae Radix	MOL002573	β-patchoulene	Nuclear receptor coactivator 2	NCOA2	Q15596
Scutellariae Radix	MOL002714	baicalein	Prostaglandin G/H synthase 1	PTGS1	P23219
Scutellariae Radix	MOL002714	baicalein	Androgen receptor	AR	P10275
Scutellariae Radix	MOL002714	baicalein	Prostaglandin G/H synthase 2	PTGS2	P35354
Scutellariae Radix	MOL002714	baicalein	Heat shock protein HSP 90	HSP90AB1	P08238
Scutellariae Radix	MOL002714	baicalein	Dipeptidyl peptidase IV	DPP4	P27487
Scutellariae Radix	MOL002714	baicalein	Trypsin-1	PRSS1	P07477
Scutellariae Radix	MOL002714	baicalein	Nuclear receptor coactivator 2	NCOA2	Q15596
Scutellariae Radix	MOL002714	baicalein	Nuclear receptor coactivator 1	NCOA1	Q15788
Scutellariae Radix	MOL002714	baicalein	Calmodulin	CAMSAP2	Q08AD1
Scutellariae Radix	MOL002714	baicalein	Transcription factor p65	RELA	Q04206
Scutellariae Radix	MOL002714	baicalein	RAC-alpha serine/threonine-protein kinase	AKT1	P31749
Scutellariae Radix	MOL002714	baicalein	Vascular endothelial growth factor A	VEGFA	P15692
Scutellariae Radix	MOL002714	baicalein	Apoptosis regulator Bcl-2	BCL2	P10415
Scutellariae Radix	MOL002714	baicalein	Proto-oncogene c-Fos	FOS	P01100
Scutellariae Radix	MOL002714	baicalein	Apoptosis regulator BAX	BAX	Q07812
Scutellariae Radix	MOL002714	baicalein	Matrix metalloproteinase-9	MMP9	P14780
Scutellariae Radix	MOL002714	baicalein	Caspase-3	CASP3	P42574
Scutellariae Radix	MOL002714	baicalein	Cellular tumor antigen p53	TP53	P04637
Scutellariae Radix	MOL002714	baicalein	Hypoxia-inducible factor 1-alpha	HIF1A	Q16665
Scutellariae Radix	MOL002714	baicalein	Fos-related antigen 1	FOSL1	P15407
Scutellariae Radix	MOL002714	baicalein	Fos-related antigen 2	FOSL2	P15408
Scutellariae Radix	MOL002714	baicalein	Cell division control protein 2 homolog	CDK1	P06493
Scutellariae Radix	MOL002714	baicalein	G2/mitotic-specific cyclin-B1	CCNB1	P14635
Scutellariae Radix	MOL002714	baicalein	Myeloperoxidase	MPO	P05164
Scutellariae Radix	MOL002714	baicalein	Aryl hydrocarbon receptor	AHR	P35869
Scutellariae Radix	MOL002714	baicalein	Insulin-like growth factor II	IGF2	P01344
Scutellariae Radix	MOL002714	baicalein	Cytochrome c	CYCS	P99999
Scutellariae Radix	MOL002714	baicalein	Nuclear factor of activated T-cells, cytoplasmic 1	NFATC1	O95644
Scutellariae Radix	MOL002714	baicalein	Tudor domain-containing protein 7	TDRD7	Q8NHU6
Scutellariae Radix	MOL002714	baicalein	Egl nine homolog 1	EGLN1	Q9GZT9
Scutellariae Radix	MOL002714	baicalein	NADPH oxidase 5	NOX5	Q96PH1
Scutellariae Radix	MOL002714	baicalein	Fatty acid-binding protein, epidermal	FABP5	Q01469
Scutellariae Radix	MOL002714	baicalein	Apolipoprotein D	APOD	P05090

Scutellariae Radix	MOL002737	scutellarein	Prostaglandin G/H synthase 1	PTGS1	P23219
Scutellariae Radix	MOL002737	scutellarein	Androgen receptor	AR	P10275
Scutellariae Radix	MOL002737	scutellarein	Prostaglandin G/H synthase 2	PTGS2	P35354
Scutellariae Radix	MOL002737	scutellarein	Heat shock protein HSP 90	HSP90AB1	P08238
Scutellariae Radix	MOL002737	scutellarein	Trypsin-1	PRSS1	P07477
Scutellariae Radix	MOL002737	scutellarein	Nuclear receptor coactivator 2	NCOA2	Q15596
Scutellariae Radix	MOL002737	scutellarein	Vascular endothelial growth factor A	VEGFA	P15692
Scutellariae Radix	MOL002909	5,7,2,5-tetrahydroxy-8,6-dimethoxyflavone	Nitric oxide synthase, inducible	NOS2	P35228
Scutellariae Radix	MOL002909	5,7,2,5-tetrahydroxy-8,6-dimethoxyflavone	Androgen receptor	AR	P10275
Scutellariae Radix	MOL002909	5,7,2,5-tetrahydroxy-8,6-dimethoxyflavone	Prostaglandin G/H synthase 2	PTGS2	P35354
Scutellariae Radix	MOL002909	5,7,2,5-tetrahydroxy-8,6-dimethoxyflavone	Dipeptidyl peptidase IV	DPP4	P27487
Scutellariae Radix	MOL002909	5,7,2,5-tetrahydroxy-8,6-dimethoxyflavone	Glycogen phosphorylase, muscle form	PYGM	P11217
Scutellariae Radix	MOL002909	5,7,2,5-tetrahydroxy-8,6-dimethoxyflavone	Heat shock protein HSP 90	HSP90AB1	P08238
Scutellariae Radix	MOL002909	5,7,2,5-tetrahydroxy-8,6-dimethoxyflavone	Trypsin-1	PRSS1	P07477
Scutellariae Radix	MOL002909	5,7,2,5-tetrahydroxy-8,6-dimethoxyflavone	Nuclear receptor coactivator 2	NCOA2	Q15596
Scutellariae Radix	MOL002910	Carthamidin	Prostaglandin G/H synthase 1	PTGS1	P23219
Scutellariae Radix	MOL002910	Carthamidin	Prostaglandin G/H synthase 2	PTGS2	P35354
Scutellariae Radix	MOL002910	Carthamidin	Heat shock protein HSP 90	HSP90AB1	P08238
Scutellariae Radix	MOL002913	Dihydrobaicalin_qt	Prostaglandin G/H synthase 1	PTGS1	P23219
Scutellariae Radix	MOL002913	Dihydrobaicalin_qt	Prostaglandin G/H synthase 2	PTGS2	P35354
Scutellariae Radix	MOL002913	Dihydrobaicalin_qt	Heat shock protein HSP 90	HSP90AB1	P08238
Scutellariae Radix	MOL002914	Eriodyctiol (flavanone)	Prostaglandin G/H synthase 1	PTGS1	P23219
Scutellariae Radix	MOL002914	Eriodyctiol (flavanone)	Prostaglandin G/H synthase 2	PTGS2	P35354
Scutellariae Radix	MOL002914	Eriodyctiol (flavanone)	Heat shock protein HSP 90	HSP90AB1	P08238
Scutellariae Radix	MOL002914	Eriodyctiol (flavanone)	Nuclear receptor coactivator 2	NCOA2	Q15596
Scutellariae Radix	MOL002914	Eriodyctiol (flavanone)	Glycogen phosphorylase, muscle form	PYGM	P11217
Scutellariae Radix	MOL002914	Eriodyctiol (flavanone)	Calmodulin	CAMSAP2	Q08AD1
Scutellariae Radix	MOL002915	Salvigenin	Nitric oxide synthase, inducible	NOS2	P35228
Scutellariae Radix	MOL002915	Salvigenin	Prostaglandin G/H synthase 1	PTGS1	P23219
Scutellariae Radix	MOL002915	Salvigenin	Sodium channel protein type 5 subunit alpha	SCN5A	Q14524
Scutellariae Radix	MOL002915	Salvigenin	Prostaglandin G/H synthase 2	PTGS2	P35354
Scutellariae Radix	MOL002915	Salvigenin	Retinoic acid receptor RXR-alpha	RXRA	P19793
Scutellariae Radix	MOL002915	Salvigenin	Acetylcholinesterase	COLQ	Q9Y215
Scutellariae Radix	MOL002915	Salvigenin	Alpha-1B adrenergic receptor	ADRA1B	P35368
Scutellariae Radix	MOL002915	Salvigenin	Beta-2 adrenergic receptor	ADRB2	P07550
Scutellariae Radix	MOL002915	Salvigenin	Dipeptidyl peptidase IV	DPP4	P27487
Scutellariae Radix	MOL002915	Salvigenin	Heat shock protein HSP 90	HSP90AB1	P08238
Scutellariae Radix	MOL002915	Salvigenin	Ig gamma-1 chain C region	IGHG1	P01857
Scutellariae Radix	MOL002915	Salvigenin	Trypsin-1	PRSS1	P07477

Scutellariae Radix	MOL002915	Salvigenin	Nuclear receptor coactivator 2	NCOA2	Q15596
Scutellariae Radix	MOL002915	Salvigenin	Calmodulin	CAMSAP2	Q08AD1
Scutellariae Radix	MOL002915	Salvigenin	Coagulation factor VII	F7	P08709
Scutellariae Radix	MOL002916	(2,6-dihydroxyphenyl)-3,5,7-trihydroxy-chromo	Nitric oxide synthase, inducible	NOS2	P35228
Scutellariae Radix	MOL002916	(2,6-dihydroxyphenyl)-3,5,7-trihydroxy-chromo	Prostaglandin G/H synthase 1	PTGS1	P23219
Scutellariae Radix	MOL002916	(2,6-dihydroxyphenyl)-3,5,7-trihydroxy-chromo	Androgen receptor	AR	P10275
Scutellariae Radix	MOL002916	(2,6-dihydroxyphenyl)-3,5,7-trihydroxy-chromo	Peroxisome proliferator activated receptor gamma	PPARG	P37231
Scutellariae Radix	MOL002916	(2,6-dihydroxyphenyl)-3,5,7-trihydroxy-chromo	Prostaglandin G/H synthase 2	PTGS2	P35354
Scutellariae Radix	MOL002916	(2,6-dihydroxyphenyl)-3,5,7-trihydroxy-chromo	Heat shock protein HSP 90	HSP90AB1	P08238
Scutellariae Radix	MOL002917	5,2',6'-Trihydroxy-7,8-dimethoxyflavone	Nitric oxide synthase, inducible	NOS2	P35228
Scutellariae Radix	MOL002917	5,2',6'-Trihydroxy-7,8-dimethoxyflavone	Prostaglandin G/H synthase 1	PTGS1	P23219
Scutellariae Radix	MOL002917	5,2',6'-Trihydroxy-7,8-dimethoxyflavone	Androgen receptor	AR	P10275
Scutellariae Radix	MOL002917	5,2',6'-Trihydroxy-7,8-dimethoxyflavone	Sodium channel protein type 5 subunit alpha	SCN5A	Q14524
Scutellariae Radix	MOL002917	5,2',6'-Trihydroxy-7,8-dimethoxyflavone	Prostaglandin G/H synthase 2	PTGS2	P35354
Scutellariae Radix	MOL002917	5,2',6'-Trihydroxy-7,8-dimethoxyflavone	Estrogen receptor beta	ESR2	Q92731
Scutellariae Radix	MOL002917	5,2',6'-Trihydroxy-7,8-dimethoxyflavone	Dipeptidyl peptidase IV	DPP4	P27487
Scutellariae Radix	MOL002917	5,2',6'-Trihydroxy-7,8-dimethoxyflavone	Heat shock protein HSP 90	HSP90AB1	P08238
Scutellariae Radix	MOL002917	5,2',6'-Trihydroxy-7,8-dimethoxyflavone	Cell division protein kinase 2	CDK2	P24941
Scutellariae Radix	MOL002917	5,2',6'-Trihydroxy-7,8-dimethoxyflavone	Serine/threonine-protein kinase Chk1	CHEK1	O14757
Scutellariae Radix	MOL002917	5,2',6'-Trihydroxy-7,8-dimethoxyflavone	Trypsin-1	PRSS1	P07477
Scutellariae Radix	MOL002917	5,2',6'-Trihydroxy-7,8-dimethoxyflavone	Nuclear receptor coactivator 2	NCOA2	Q15596
Scutellariae Radix	MOL002917	5,2',6'-Trihydroxy-7,8-dimethoxyflavone	Calmodulin	CAMSAP2	Q08AD1
Scutellariae Radix	MOL002918	Ganhuangenin	Nitric oxide synthase, inducible	NOS2	P35228
Scutellariae Radix	MOL002918	Ganhuangenin	Androgen receptor	AR	P10275
Scutellariae Radix	MOL002918	Ganhuangenin	Prostaglandin G/H synthase 2	PTGS2	P35354
Scutellariae Radix	MOL002918	Ganhuangenin	Coagulation factor VII	F7	P08709
Scutellariae Radix	MOL002918	Ganhuangenin	Estrogen receptor beta	ESR2	Q92731
Scutellariae Radix	MOL002918	Ganhuangenin	Dipeptidyl peptidase IV	DPP4	P27487
Scutellariae Radix	MOL002918	Ganhuangenin	Trypsin-1	PRSS1	P07477
Scutellariae Radix	MOL002922	5-(2-hydroxyethyl)-2-methoxyphenol	Prostaglandin G/H synthase 1	PTGS1	P23219
Scutellariae Radix	MOL002922	5-(2-hydroxyethyl)-2-methoxyphenol	Beta-1 adrenergic receptor	ADRB1	P08588
Scutellariae Radix	MOL002922	5-(2-hydroxyethyl)-2-methoxyphenol	Prostaglandin G/H synthase 2	PTGS2	P35354
Scutellariae Radix	MOL002922	5-(2-hydroxyethyl)-2-methoxyphenol	Alpha-2A adrenergic receptor	ADRA2A	P08913
Scutellariae Radix	MOL002922	5-(2-hydroxyethyl)-2-methoxyphenol	Alpha-2C adrenergic receptor	ADRA2C	P18825
Scutellariae Radix	MOL002922	5-(2-hydroxyethyl)-2-methoxyphenol	Sodium-dependent noradrenaline transporter	SLC6A2	P23975
Scutellariae Radix	MOL002922	5-(2-hydroxyethyl)-2-methoxyphenol	Alpha-1A adrenergic receptor	ADRA1A	P35348
Scutellariae Radix	MOL002922	5-(2-hydroxyethyl)-2-methoxyphenol	Alpha-2B adrenergic receptor	ADRA2B	P18089
Scutellariae Radix	MOL002922	5-(2-hydroxyethyl)-2-methoxyphenol	Sodium-dependent dopamine transporter	SLC6A3	Q01959
Scutellariae Radix	MOL002922	5-(2-hydroxyethyl)-2-methoxyphenol	Beta-2 adrenergic receptor	ADRB2	P07550

Scutellariae Radix	MOL002922	5-(2-hydroxyethyl)-2-methoxyphenol	Beta-lactamase	DPEP1	P16444
Scutellariae Radix	MOL002922	5-(2-hydroxyethyl)-2-methoxyphenol	Amine oxidase [flavin-containing] B	MAOB	P27338
Scutellariae Radix	MOL002922	5-(2-hydroxyethyl)-2-methoxyphenol	Amine oxidase [flavin-containing] A	MAOA	P21397
Scutellariae Radix	MOL002922	5-(2-hydroxyethyl)-2-methoxyphenol	Lysozyme	LYZ	P61626
Scutellariae Radix	MOL002922	5-(2-hydroxyethyl)-2-methoxyphenol	Muscarinic acetylcholine receptor M1	CHRM1	P11229
Scutellariae Radix	MOL002924	darendoside B_qt	Nitric oxide synthase, inducible	NOS2	P35228
Scutellariae Radix	MOL002924	darendoside B_qt	Estrogen receptor	ESR1	P03372
Scutellariae Radix	MOL002924	darendoside B_qt	Prostaglandin G/H synthase 2	PTGS2	P35354
Scutellariae Radix	MOL002924	darendoside B_qt	Dipeptidyl peptidase IV	DPP4	P27487
Scutellariae Radix	MOL002924	darendoside B_qt	Cell division protein kinase 2	CDK2	P24941
Scutellariae Radix	MOL002924	darendoside B_qt	Beta-lactamase	DPEP1	P16444
Scutellariae Radix	MOL002925	5,7,2',6'-Tetrahydroxyflavone	Prostaglandin G/H synthase 1	PTGS1	P23219
Scutellariae Radix	MOL002925	5,7,2',6'-Tetrahydroxyflavone	Androgen receptor	AR	P10275
Scutellariae Radix	MOL002925	5,7,2',6'-Tetrahydroxyflavone	Prostaglandin G/H synthase 2	PTGS2	P35354
Scutellariae Radix	MOL002925	5,7,2',6'-Tetrahydroxyflavone	Dipeptidyl peptidase IV	DPP4	P27487
Scutellariae Radix	MOL002925	5,7,2',6'-Tetrahydroxyflavone	Heat shock protein HSP 90	HSP90AB1	P08238
Scutellariae Radix	MOL002927	Skullcapflavone II	Nitric oxide synthase, inducible	NOS2	P35228
Scutellariae Radix	MOL002927	Skullcapflavone II	Prostaglandin G/H synthase 1	PTGS1	P23219
Scutellariae Radix	MOL002927	Skullcapflavone II	Potassium voltage-gated channel subfamily H member 2	KCNH2	Q12809
Scutellariae Radix	MOL002927	Skullcapflavone II	Androgen receptor	AR	P10275
Scutellariae Radix	MOL002927	Skullcapflavone II	Sodium channel protein type 5 subunit alpha	SCN5A	Q14524
Scutellariae Radix	MOL002927	Skullcapflavone II	Prostaglandin G/H synthase 2	PTGS2	P35354
Scutellariae Radix	MOL002927	Skullcapflavone II	Coagulation factor VII	F7	P08709
Scutellariae Radix	MOL002927	Skullcapflavone II	Vascular endothelial growth factor receptor 2	KDR	P35968
Scutellariae Radix	MOL002927	Skullcapflavone II	Voltage-dependent calcium channel subunit alpha-2/delta-1	CACNA2D1	P54289
Scutellariae Radix	MOL002927	Skullcapflavone II	Dipeptidyl peptidase IV	DPP4	P27487
Scutellariae Radix	MOL002927	Skullcapflavone II	Heat shock protein HSP 90	HSP90AB1	P08238
Scutellariae Radix	MOL002927	Skullcapflavone II	Ig gamma-1 chain C region	IGHG1	P01857
Scutellariae Radix	MOL002927	Skullcapflavone II	Trypsin-1	PRSS1	P07477
Scutellariae Radix	MOL002927	Skullcapflavone II	Nuclear receptor coactivator 2	NCOA2	Q15596
Scutellariae Radix	MOL002927	Skullcapflavone II	Nuclear receptor coactivator 1	NCOA1	Q15788
Scutellariae Radix	MOL002927	Skullcapflavone II	Calmodulin	CAMSAP2	Q08AD1
Scutellariae Radix	MOL002928	oroxylin a	Nitric oxide synthase, inducible	NOS2	P35228
Scutellariae Radix	MOL002928	oroxylin a	Prostaglandin G/H synthase 1	PTGS1	P23219
Scutellariae Radix	MOL002928	oroxylin a	Androgen receptor	AR	P10275
Scutellariae Radix	MOL002928	oroxylin a	Sodium channel protein type 5 subunit alpha	SCN5A	Q14524
Scutellariae Radix	MOL002928	oroxylin a	Prostaglandin G/H synthase 2	PTGS2	P35354
Scutellariae Radix	MOL002928	oroxylin a	Retinoic acid receptor RXR-alpha	RXRA	P19793
Scutellariae Radix	MOL002928	oroxylin a	Alpha-1B adrenergic receptor	ADRA1B	P35368

Scutellariae Radix	MOL002928	oroxylin a	Beta-2 adrenergic receptor	ADRB2	P07550
Scutellariae Radix	MOL002928	oroxylin a	Dipeptidyl peptidase IV	DPP4	P27487
Scutellariae Radix	MOL002928	oroxylin a	Heat shock protein HSP 90	HSP90AB1	P08238
Scutellariae Radix	MOL002928	oroxylin a	Trypsin-1	PRSS1	P07477
Scutellariae Radix	MOL002928	oroxylin a	Nuclear receptor coactivator 1	NCOA1	Q15788
Scutellariae Radix	MOL002928	oroxylin a	Calmodulin	CAMSAP2	Q08AD1
Scutellariae Radix	MOL002928	oroxylin a	Nuclear receptor coactivator 2	NCOA2	Q15596
Scutellariae Radix	MOL002928	oroxylin a	cAMP-dependent protein kinase inhibitor alpha	PKIA	P61925
Scutellariae Radix	MOL002928	oroxylin a	Apoptosis regulator Bcl-2	BCL2	P10415
Scutellariae Radix	MOL002928	oroxylin a	Interleukin-6	IL6R	P08887
Scutellariae Radix	MOL002928	oroxylin a	Caspase-3	CASP3	P42574
Scutellariae Radix	MOL002928	oroxylin a	Cell division control protein 2 homolog	CDK1	P06493
Scutellariae Radix	MOL002928	oroxylin a	Cytochrome P450 1A2	CYP1A2	P05177
Scutellariae Radix	MOL002928	oroxylin a	G2/mitotic-specific cyclin-B1	CCNB1	P14635
Scutellariae Radix	MOL002928	oroxylin a	Cell division protein kinase 7	CDK7	P50613
Scutellariae Radix	MOL002928	oroxylin a	Cytochrome P450 2C9	CYP2C9	P11712
Scutellariae Radix	MOL002930	Tyrosol	Beta-1 adrenergic receptor	ADRB1	P08588
Scutellariae Radix	MOL002930	Tyrosol	Sodium-dependent noradrenaline transporter	SLC6A2	P23975
Scutellariae Radix	MOL002930	Tyrosol	Alpha-1A adrenergic receptor	ADRA1A	P35348
Scutellariae Radix	MOL002930	Tyrosol	Sodium-dependent dopamine transporter	SLC6A3	Q01959
Scutellariae Radix	MOL002930	Tyrosol	Beta-lactamase	DPEP1	P16444
Scutellariae Radix	MOL002930	Tyrosol	Amine oxidase [flavin-containing] A	MAOA	P21397
Scutellariae Radix	MOL002930	Tyrosol	Alcohol dehydrogenase 1C	ADH1C	P00326
Scutellariae Radix	MOL002930	Tyrosol	Lysozyme	lYZ	P61626
Scutellariae Radix	MOL002930	Tyrosol	Trypsin-3	PRSS3	P35030
Scutellariae Radix	MOL002930	Tyrosol	Alpha-1B adrenergic receptor	ADRA1B	P35368
Scutellariae Radix	MOL002930	Tyrosol	Amine oxidase [flavin-containing] B	MAOB	P27338
Scutellariae Radix	MOL002931	scutellarin	Bcl-2-like protein 1	BCL2L1	Q07817
Scutellariae Radix	MOL002931	scutellarin	Activator of 90 kDa heat shock protein ATPase homolog 1	AHSA1	O95433
Scutellariae Radix	MOL002931	scutellarin	Caspase-3	CASP3	P42574
Scutellariae Radix	MOL002931	scutellarin	Protein kinase C gamma type	PRKCG	P05129
Scutellariae Radix	MOL002932	Panicolin	Nitric oxide synthase, inducible	NOS2	P35228
Scutellariae Radix	MOL002932	Panicolin	Prostaglandin G/H synthase 1	PTGS1	P23219
Scutellariae Radix	MOL002932	Panicolin	Androgen receptor	AR	P10275
Scutellariae Radix	MOL002932	Panicolin	Sodium channel protein type 5 subunit alpha	SCN5A	Q14524
Scutellariae Radix	MOL002932	Panicolin	Prostaglandin G/H synthase 2	PTGS2	P35354
Scutellariae Radix	MOL002932	Panicolin	Estrogen receptor beta	ESR2	Q92731
Scutellariae Radix	MOL002932	Panicolin	Dipeptidyl peptidase IV	DPP4	P27487
Scutellariae Radix	MOL002932	Panicolin	Heat shock protein HSP 90	HSP90AB1	P08238

Scutellariae Radix	MOL002932	Panicolin	Cell division protein kinase 2	CDK2	P24941
Scutellariae Radix	MOL002932	Panicolin	Serine/threonine-protein kinase Chk1	CHEK1	O14757
Scutellariae Radix	MOL002932	Panicolin	Trypsin-1	PRSS1	P07477
Scutellariae Radix	MOL002932	Panicolin	Calmodulin	CAMSAP2	Q08AD1
Scutellariae Radix	MOL002932	Panicolin	Nuclear receptor coactivator 1	NCOA1	Q15788
Scutellariae Radix	MOL002933	5,7,4'-Trihydroxy-8-methoxyflavone	Nitric oxide synthase, inducible	NOS2	P35228
Scutellariae Radix	MOL002933	5,7,4'-Trihydroxy-8-methoxyflavone	Prostaglandin G/H synthase 1	PTGS1	P23219
Scutellariae Radix	MOL002933	5,7,4'-Trihydroxy-8-methoxyflavone	Estrogen receptor	ESR1	P03372
Scutellariae Radix	MOL002933	5,7,4'-Trihydroxy-8-methoxyflavone	Androgen receptor	AR	P10275
Scutellariae Radix	MOL002933	5,7,4'-Trihydroxy-8-methoxyflavone	Peroxisome proliferator activated receptor gamma	PPARG	P37231
Scutellariae Radix	MOL002933	5,7,4'-Trihydroxy-8-methoxyflavone	Prostaglandin G/H synthase 2	PTGS2	P35354
Scutellariae Radix	MOL002933	5,7,4'-Trihydroxy-8-methoxyflavone	Dipeptidyl peptidase IV	DPP4	P27487
Scutellariae Radix	MOL002933	5,7,4'-Trihydroxy-8-methoxyflavone	Glycogen phosphorylase, muscle form	PYGM	P11217
Scutellariae Radix	MOL002933	5,7,4'-Trihydroxy-8-methoxyflavone	Mitogen-activated protein kinase 14	MAPK14	Q16539
Scutellariae Radix	MOL002933	5,7,4'-Trihydroxy-8-methoxyflavone	Glycogen synthase kinase-3 beta	GSK3B	P49841
Scutellariae Radix	MOL002933	5,7,4'-Trihydroxy-8-methoxyflavone	Heat shock protein HSP 90	HSP90AB1	P08238
Scutellariae Radix	MOL002933	5,7,4'-Trihydroxy-8-methoxyflavone	Cell division protein kinase 2	CDK2	P24941
Scutellariae Radix	MOL002933	5,7,4'-Trihydroxy-8-methoxyflavone	Serine/threonine-protein kinase Chk1	CHEK1	O14757
Scutellariae Radix	MOL002933	5,7,4'-Trihydroxy-8-methoxyflavone	Trypsin-1	PRSS1	P07477
Scutellariae Radix	MOL002933	5,7,4'-Trihydroxy-8-methoxyflavone	Nuclear receptor coactivator 2	NCOA2	Q15596
Scutellariae Radix	MOL002933	5,7,4'-Trihydroxy-8-methoxyflavone	Calmodulin	CAMSAP2	Q08AD1
Scutellariae Radix	MOL002934	NEOBAICALEIN	Nitric oxide synthase, inducible	NOS2	P35228
Scutellariae Radix	MOL002934	NEOBAICALEIN	Potassium voltage-gated channel subfamily H member 2	KCNH2	Q12809
Scutellariae Radix	MOL002934	NEOBAICALEIN	Estrogen receptor	ESR1	P03372
Scutellariae Radix	MOL002934	NEOBAICALEIN	Androgen receptor	AR	P10275
Scutellariae Radix	MOL002934	NEOBAICALEIN	Sodium channel protein type 5 subunit alpha	SCN5A	Q14524
Scutellariae Radix	MOL002934	NEOBAICALEIN	Peroxisome proliferator activated receptor gamma	PPARG	P37231
Scutellariae Radix	MOL002934	NEOBAICALEIN	Prostaglandin G/H synthase 2	PTGS2	P35354
Scutellariae Radix	MOL002934	NEOBAICALEIN	Coagulation factor VII	F7	P08709
Scutellariae Radix	MOL002934	NEOBAICALEIN	Estrogen receptor beta	ESR2	Q92731
Scutellariae Radix	MOL002934	NEOBAICALEIN	Dipeptidyl peptidase IV	DPP4	P27487
Scutellariae Radix	MOL002934	NEOBAICALEIN	Glycogen phosphorylase, muscle form	PYGM	P11217
Scutellariae Radix	MOL002934	NEOBAICALEIN	Glycogen synthase kinase-3 beta	GSK3B	P49841
Scutellariae Radix	MOL002934	NEOBAICALEIN	Heat shock protein HSP 90	HSP90AB1	P08238
Scutellariae Radix	MOL002934	NEOBAICALEIN	Serine/threonine-protein kinase Chk1	CHEK1	O14757
Scutellariae Radix	MOL002934	NEOBAICALEIN	Trypsin-1	PRSS1	P07477
Scutellariae Radix	MOL002934	NEOBAICALEIN	Nuclear receptor coactivator 2	NCOA2	Q15596
Scutellariae Radix	MOL002934	NEOBAICALEIN	Calmodulin	CAMSAP2	Q08AD1
Scutellariae Radix	MOL002936	5,8-Dihydroxy-6,7-dimethoxyflavone	Nitric oxide synthase, inducible	NOS2	P35228

Scutellariae Radix	MOL002936	5,8-Dihydroxy-6,7-dimethoxyflavone	Prostaglandin G/H synthase 1	PTGS1	P23219
Scutellariae Radix	MOL002936	5,8-Dihydroxy-6,7-dimethoxyflavone	Estrogen receptor	ESR1	P03372
Scutellariae Radix	MOL002936	5,8-Dihydroxy-6,7-dimethoxyflavone	Androgen receptor	AR	P10275
Scutellariae Radix	MOL002936	5,8-Dihydroxy-6,7-dimethoxyflavone	Sodium channel protein type 5 subunit alpha	SCN5A	Q14524
Scutellariae Radix	MOL002936	5,8-Dihydroxy-6,7-dimethoxyflavone	Peroxisome proliferator activated receptor gamma	PPARG	P37231
Scutellariae Radix	MOL002936	5,8-Dihydroxy-6,7-dimethoxyflavone	Prostaglandin G/H synthase 2	PTGS2	P35354
Scutellariae Radix	MOL002936	5,8-Dihydroxy-6,7-dimethoxyflavone	Coagulation factor VII	F7	P08709
Scutellariae Radix	MOL002936	5,8-Dihydroxy-6,7-dimethoxyflavone	Estrogen receptor beta	ESR2	Q92731
Scutellariae Radix	MOL002936	5,8-Dihydroxy-6,7-dimethoxyflavone	Dipeptidyl peptidase IV	DPP4	P27487
Scutellariae Radix	MOL002936	5,8-Dihydroxy-6,7-dimethoxyflavone	Peroxisome proliferator activated receptor delta	PPARD	Q03181
Scutellariae Radix	MOL002936	5,8-Dihydroxy-6,7-dimethoxyflavone	Mitogen-activated protein kinase 14	MAPK14	Q16539
Scutellariae Radix	MOL002936	5,8-Dihydroxy-6,7-dimethoxyflavone	Glycogen synthase kinase-3 beta	GSK3B	P49841
Scutellariae Radix	MOL002936	5,8-Dihydroxy-6,7-dimethoxyflavone	Heat shock protein HSP 90	HSP90AB1	P08238
Scutellariae Radix	MOL002936	5,8-Dihydroxy-6,7-dimethoxyflavone	Cell division protein kinase 2	CDK2	P24941
Scutellariae Radix	MOL002936	5,8-Dihydroxy-6,7-dimethoxyflavone	Serine/threonine-protein kinase Chk1	CHEK1	O14757
Scutellariae Radix	MOL002936	5,8-Dihydroxy-6,7-dimethoxyflavone	Trypsin-1	PRSS1	P07477
Scutellariae Radix	MOL002936	5,8-Dihydroxy-6,7-dimethoxyflavone	Nuclear receptor coactivator 2	NCOA2	Q15596
Scutellariae Radix	MOL002936	5,8-Dihydroxy-6,7-dimethoxyflavone	Nuclear receptor coactivator 1	NCOA1	Q15788
Scutellariae Radix	MOL002936	5,8-Dihydroxy-6,7-dimethoxyflavone	Calmodulin	CAMSAP2	Q08AD1
Scutellariae Radix	MOL002937	DIHYDROOROXYLIN	Prostaglandin G/H synthase 1	PTGS1	P23219
Scutellariae Radix	MOL002937	DIHYDROOROXYLIN	Sodium channel protein type 5 subunit alpha	SCN5A	Q14524
Scutellariae Radix	MOL002937	DIHYDROOROXYLIN	Prostaglandin G/H synthase 2	PTGS2	P35354
Scutellariae Radix	MOL002937	DIHYDROOROXYLIN	Retinoic acid receptor RXR-alpha	RXRA	P19793
Scutellariae Radix	MOL002937	DIHYDROOROXYLIN	Alpha-1B adrenergic receptor	ADRA1B	P35368
Scutellariae Radix	MOL002937	DIHYDROOROXYLIN	Beta-2 adrenergic receptor	ADRB2	P07550
Scutellariae Radix	MOL002937	DIHYDROOROXYLIN	Heat shock protein HSP 90	HSP90AB1	P08238
Scutellariae Radix	MOL002937	DIHYDROOROXYLIN	Calmodulin	CAMSAP2	Q08AD1
Scutellariae Radix	MOL002937	DIHYDROOROXYLIN	Nuclear receptor coactivator 1	NCOA1	Q15788
Scutellariae Radix	MOL000357	Sitogluside	Progesterone receptor	PGR	P06401
Scutellariae Radix	MOL000357	Sitogluside	Prostaglandin G/H synthase 1	PTGS1	P23219
Scutellariae Radix	MOL000357	Sitogluside	Muscarinic acetylcholine receptor M3	CHRM3	P20309
Scutellariae Radix	MOL000357	Sitogluside	Potassium voltage-gated channel subfamily H member 2	KCNH2	Q12809
Scutellariae Radix	MOL000357	Sitogluside	Muscarinic acetylcholine receptor M1	CHRM1	P11229
Scutellariae Radix	MOL000357	Sitogluside	Sodium channel protein type 5 subunit alpha	SCN5A	Q14524
Scutellariae Radix	MOL000357	Sitogluside	Prostaglandin G/H synthase 2	PTGS2	P35354
Scutellariae Radix	MOL000357	Sitogluside	5-hydroxytryptamine receptor 3A	HTR3A	P46098
Scutellariae Radix	MOL000357	Sitogluside	Retinoic acid receptor RXR-alpha	RXRA	P19793
Scutellariae Radix	MOL000357	Sitogluside	Alpha-1B adrenergic receptor	ADRA1B	P35368
Scutellariae Radix	MOL000357	Sitogluside	Beta-2 adrenergic receptor	ADRB2	P07550

Scutellariae Radix	MOL000357	Sitogluside	Alpha-1D adrenergic receptor	ADRA1D	P25100
Scutellariae Radix	MOL000357	Sitogluside	Heat shock protein HSP 90	HSP90AB1	P08238
Scutellariae Radix	MOL000357	Sitogluside	Nuclear receptor coactivator 2	NCOA2	Q15596
Scutellariae Radix	MOL000357	Sitogluside	Calmodulin	CAMSAP2	Q08AD1
Scutellariae Radix	MOL000358	beta-sitosterol	Progesterone receptor	PGR	P06401
Scutellariae Radix	MOL000358	beta-sitosterol	Nuclear receptor coactivator 2	NCOA2	Q15596
Scutellariae Radix	MOL000358	beta-sitosterol	Prostaglandin G/H synthase 1	PTGS1	P23219
Scutellariae Radix	MOL000358	beta-sitosterol	Prostaglandin G/H synthase 2	PTGS2	P35354
Scutellariae Radix	MOL000358	beta-sitosterol	Heat shock protein HSP 90	HSP90AB1	P08238
Scutellariae Radix	MOL000358	beta-sitosterol	Potassium voltage-gated channel subfamily H member 2	KCNH2	Q12809
Scutellariae Radix	MOL000358	beta-sitosterol	Dopamine D1 receptor	DRD1	P21918
Scutellariae Radix	MOL000358	beta-sitosterol	Muscarinic acetylcholine receptor M3	CHRM3	P20309
Scutellariae Radix	MOL000358	beta-sitosterol	Muscarinic acetylcholine receptor M1	CHRM1	P11229
Scutellariae Radix	MOL000358	beta-sitosterol	Sodium channel protein type 5 subunit alpha	SCN5A	Q14524
Scutellariae Radix	MOL000358	beta-sitosterol	Muscarinic acetylcholine receptor M4	CHRM4	P08173
Scutellariae Radix	MOL000358	beta-sitosterol	Alpha-1A adrenergic receptor	ADRA1A	P25100
Scutellariae Radix	MOL000358	beta-sitosterol	Muscarinic acetylcholine receptor M2	CHRM2	P08172
Scutellariae Radix	MOL000358	beta-sitosterol	Alpha-1B adrenergic receptor	ADRA1B	P35368
Scutellariae Radix	MOL000358	beta-sitosterol	Beta-2 adrenergic receptor	ADRB2	P07550
Scutellariae Radix	MOL000358	beta-sitosterol	Neuronal acetylcholine receptor subunit alpha-2	CHRNA2	Q15822
Scutellariae Radix	MOL000358	beta-sitosterol	Sodium-dependent serotonin transporter	SLC6A4	P31645
Scutellariae Radix	MOL000358	beta-sitosterol	Mu-type opioid receptor	OPRM1	P35372
Scutellariae Radix	MOL000358	beta-sitosterol	Gamma-aminobutyric acid receptor subunit alpha-1	GABRA1	P14867
Scutellariae Radix	MOL000358	beta-sitosterol	Apoptosis regulator Bcl-2	BCL2	O60238
Scutellariae Radix	MOL000358	beta-sitosterol	Apoptosis regulator BAX	BAX	Q07812
Scutellariae Radix	MOL000358	beta-sitosterol	Caspase-9	CASP9	P55211
Scutellariae Radix	MOL000358	beta-sitosterol	Transcription factor AP-1	JUN	P05412
Scutellariae Radix	MOL000358	beta-sitosterol	Caspase-3	CASP3	P42574
Scutellariae Radix	MOL000358	beta-sitosterol	Caspase-8	CASP8	Q14790
Scutellariae Radix	MOL000358	beta-sitosterol	Protein kinase C alpha type	PRKCA	P17252
Scutellariae Radix	MOL000358	beta-sitosterol	Serum paraoxonase/arylesterase 1	PON1	P27169
Scutellariae Radix	MOL000358	beta-sitosterol	Microtubule-associated protein 2	MAP2	P11137
Scutellariae Radix	MOL000359	sitosterol	Progesterone receptor	PGR	P06401
Scutellariae Radix	MOL000359	sitosterol	Nuclear receptor coactivator 2	NCOA2	Q15596
Scutellariae Radix	MOL000359	sitosterol	Mineralocorticoid receptor	NR3C2	P08235
Scutellariae Radix	MOL000396	(+)-Syringaresinol	Potassium voltage-gated channel subfamily H member 2	KCNH2	Q12809
Scutellariae Radix	MOL000396	(+)-Syringaresinol	Sodium channel protein type 5 subunit alpha	SCN5A	Q14524
Scutellariae Radix	MOL000396	(+)-Syringaresinol	Prostaglandin G/H synthase 2	PTGS2	P35354
Scutellariae Radix	MOL000396	(+)-Syringaresinol	Nuclear receptor coactivator 2	NCOA2	Q15596

Scutellariae Radix	MOL000396	(+)-Syringaresinol	Calmodulin	CAMSAP2	Q08AD1
Scutellariae Radix	MOL000396	(+)-Syringaresinol	Heat shock protein HSP 90	HSP90AB1	P08238
Scutellariae Radix	MOL000458	campesterol	Progesterone receptor	PGR	P06401
Scutellariae Radix	MOL000525	Norwogonin	Nitric oxide synthase, inducible	NOS2	P35228
Scutellariae Radix	MOL000525	Norwogonin	Prostaglandin G/H synthase 1	PTGS1	P23219
Scutellariae Radix	MOL000525	Norwogonin	Androgen receptor	AR	P10275
Scutellariae Radix	MOL000525	Norwogonin	Peroxisome proliferator activated receptor gamma	PPARG	P37231
Scutellariae Radix	MOL000525	Norwogonin	Prostaglandin G/H synthase 2	PTGS2	P35354
Scutellariae Radix	MOL000525	Norwogonin	Dipeptidyl peptidase IV	DPP4	P27487
Scutellariae Radix	MOL000525	Norwogonin	Heat shock protein HSP 90	HSP90AB1	P08238
Scutellariae Radix	MOL000525	Norwogonin	Cell division protein kinase 2	CDK2	P24941
Scutellariae Radix	MOL000525	Norwogonin	Serine/threonine-protein kinase Chk1	CHEK1	O14757
Scutellariae Radix	MOL000552	5,2'-Dihydroxy-6,7,8-trimethoxyflavone	Nitric oxide synthase, inducible	NOS2	P35228
Scutellariae Radix	MOL000552	5,2'-Dihydroxy-6,7,8-trimethoxyflavone	Prostaglandin G/H synthase 1	PTGS1	P23219
Scutellariae Radix	MOL000552	5,2'-Dihydroxy-6,7,8-trimethoxyflavone	Potassium voltage-gated channel subfamily H member 2	KCNH2	Q12809
Scutellariae Radix	MOL000552	5,2'-Dihydroxy-6,7,8-trimethoxyflavone	Androgen receptor	AR	P10275
Scutellariae Radix	MOL000552	5,2'-Dihydroxy-6,7,8-trimethoxyflavone	Sodium channel protein type 5 subunit alpha	SCN5A	Q14524
Scutellariae Radix	MOL000552	5,2'-Dihydroxy-6,7,8-trimethoxyflavone	Prostaglandin G/H synthase 2	PTGS2	P35354
Scutellariae Radix	MOL000552	5,2'-Dihydroxy-6,7,8-trimethoxyflavone	Coagulation factor VII	F7	P08709
Scutellariae Radix	MOL000552	5,2'-Dihydroxy-6,7,8-trimethoxyflavone	Estrogen receptor beta	ESR2	Q92731
Scutellariae Radix	MOL000552	5,2'-Dihydroxy-6,7,8-trimethoxyflavone	Dipeptidyl peptidase IV	DPP4	P27487
Scutellariae Radix	MOL000552	5,2'-Dihydroxy-6,7,8-trimethoxyflavone	Peroxisome proliferator activated receptor delta	PPARD	Q03181
Scutellariae Radix	MOL000552	5,2'-Dihydroxy-6,7,8-trimethoxyflavone	Heat shock protein HSP 90	HSP90AB1	P08238
Scutellariae Radix	MOL000552	5,2'-Dihydroxy-6,7,8-trimethoxyflavone	Trypsin-1	PRSS1	P07477
Scutellariae Radix	MOL000552	5,2'-Dihydroxy-6,7,8-trimethoxyflavone	Nuclear receptor coactivator 2	NCOA2	Q15596
Scutellariae Radix	MOL000552	5,2'-Dihydroxy-6,7,8-trimethoxyflavone	Calmodulin	CAMSAP2	Q08AD1
Scutellariae Radix	MOL000552	5,2'-Dihydroxy-6,7,8-trimethoxyflavone	Vascular endothelial growth factor receptor 2	KDR	P35968
Scutellariae Radix	MOL000552	5,2'-Dihydroxy-6,7,8-trimethoxyflavone	Nuclear receptor coactivator 1	NCOA1	Q15788
