# Supplementary material for: Network Pharmacology-Based Analysis on the Potential Biological Mechanisms of Yinzhihuang Oral Liquid in Treating Neonatal Hyperbilirubinemia
Source: Evid Based Complement Alternat Med. 2022 Oct 5;2022:1672670. doi: 10.1155/2022/1672670 (PMC9556251; doi:10.1155/2022/1672670)
Supplement: Supplementary Materials — Table S1: active herbal ingredients in Scutellariae Radix. Table S2: active herbal ingredients in Lonicerae Japonicae Flos. Table S3: active herbal ingredients in Artemisiae Scopariae Herba. Table S4: active herbal ingredients in Gardeniae Fructus. Table S5: ingredients in Scutellariae Radix and corresponding targets. Table S6: ingredients in Lonicerae Japonicae Flos and corresponding targets. Table S7: ingredients in Artemisiae Scopariae Herba and corresponding targets. Table S8: ingredients in Gardeniae Fructus and corresponding targets. Table S9: compound-common target network of YZH and neonatal hyperbilirubinemia. Table S10: PPI network into Cytoscape for YZH and neonatal hyperbilirubinemia analysis (minimum required interaction score of 0.9). Table S11: Gene Ontology (GO) Biological Process analysis (p < 0.05). [file 1672670.f1.zip › Table S6.pdf]

Table S6 Ingredients in Lonicerae Japonicae Flos and corresponding targets

Ingredients	MOL_ID	Molecule_Name	Protein name	Gene Name	Uniprot
Lonicerae Japonicae Flos	MOL000116	Nonanal	Alcohol dehydrogenase 1B	ADH1B	P00325
Lonicerae Japonicae Flos	MOL000116	Nonanal	Alcohol dehydrogenase 1C	ADH1C	P00326
Lonicerae Japonicae Flos	MOL000116	Nonanal	Gamma-aminobutyric acid receptor subunit alpha-1	GABRA1	P14867
Lonicerae Japonicae Flos	MOL001168	(1S,2S)-2-isopropenyl-4-isopropylidene-1-methyl-1-vinylcyclohexane	Prostaglandin G/H synthase 1	PTGS1	P23219
Lonicerae Japonicae Flos	MOL001168	(1S,2S)-2-isopropenyl-4-isopropylidene-1-methyl-1-vinylcyclohexane	Prostaglandin G/H synthase 2	PTGS2	P35354
Lonicerae Japonicae Flos	MOL001168	(1S,2S)-2-isopropenyl-4-isopropylidene-1-methyl-1-vinylcyclohexane	Retinoic acid receptor RXR-alpha	RXRA	P19793
Lonicerae Japonicae Flos	MOL001168	(1S,2S)-2-isopropenyl-4-isopropylidene-1-methyl-1-vinylcyclohexane	Sodium-dependent noradrenaline transporter	SLC6A2	P23975
Lonicerae Japonicae Flos	MOL001168	(1S,2S)-2-isopropenyl-4-isopropylidene-1-methyl-1-vinylcyclohexane	Gamma-aminobutyric acid receptor subunit alpha-1	GABRA1	P14867
Lonicerae Japonicae Flos	MOL001168	(1S,2S)-2-isopropenyl-4-isopropylidene-1-methyl-1-vinylcyclohexane	Muscarinic acetylcholine receptor M3	CHRM3	P20309
Lonicerae Japonicae Flos	MOL001168	(1S,2S)-2-isopropenyl-4-isopropylidene-1-methyl-1-vinylcyclohexane	Muscarinic acetylcholine receptor M1	CHRM1	P11229
Lonicerae Japonicae Flos	MOL001168	(1S,2S)-2-isopropenyl-4-isopropylidene-1-methyl-1-vinylcyclohexane	Muscarinic acetylcholine receptor M2	CHRM2	P08172
Lonicerae Japonicae Flos	MOL001180	γ-muurolene	Prostaglandin G/H synthase 1	PTGS1	P23219
Lonicerae Japonicae Flos	MOL001180	γ-muurolene	Muscarinic acetylcholine receptor M3	CHRM3	P20309
Lonicerae Japonicae Flos	MOL001180	γ-muurolene	Muscarinic acetylcholine receptor M1	CHRM1	P11229
Lonicerae Japonicae Flos	MOL001180	γ-muurolene	Prostaglandin G/H synthase 2	PTGS2	P35354
Lonicerae Japonicae Flos	MOL001180	γ-muurolene	Retinoic acid receptor RXR-alpha	RXRA	P19793
Lonicerae Japonicae Flos	MOL001180	γ-muurolene	Sodium-dependent noradrenaline transporter	SLC6A2	P23975
Lonicerae Japonicae Flos	MOL001180	γ-muurolene	Gamma-aminobutyric acid receptor subunit alpha-1	GABRA1	P14867
Lonicerae Japonicae Flos	MOL001180	γ-muurolene	Nuclear receptor coactivator 2	NCOA2	Q15596
Lonicerae Japonicae Flos	MOL000123	geraniol	Alcohol dehydrogenase 1B	ADH1B	P00325
Lonicerae Japonicae Flos	MOL000123	geraniol	Alcohol dehydrogenase 1C	ADH1C	P00326
Lonicerae Japonicae Flos	MOL000123	geraniol	Progesterone receptor	PGR	P06401
Lonicerae Japonicae Flos	MOL000123	geraniol	G1/S-specific cyclin-D1	CCND1	P24385
Lonicerae Japonicae Flos	MOL000123	geraniol	Mitogen-activated protein kinase 3	MAPK3	P27361
Lonicerae Japonicae Flos	MOL000123	geraniol	Cell division protein kinase 4	CDK4	P11802
Lonicerae Japonicae Flos	MOL000123	geraniol	Bcl-2 homologous antagonist/killer	BAK1	Q16611
Lonicerae Japonicae Flos	MOL000123	geraniol	Protein kinase C beta type	PRKCB	P05771
Lonicerae Japonicae Flos	MOL000123	geraniol	3-hydroxy-3-methylglutaryl-coenzyme A reductase	HMGCR	P04035
Lonicerae Japonicae Flos	MOL000123	geraniol	Cytochrome P450 2B6	CYP2B6	P20813
Lonicerae Japonicae Flos	MOL000123	geraniol	Lactase-phlorizin hydrolase	LCT	P09848
Lonicerae Japonicae Flos	MOL000125	(-)-alpha-Pinene	Gamma-aminobutyric acid receptor subunit alpha-1	GABRA1	P14867
Lonicerae Japonicae Flos	MOL001283	C09704	Prostaglandin G/H synthase 2	PTGS2	P35354
Lonicerae Japonicae Flos	MOL001283	C09704	Nuclear receptor coactivator 2	NCOA2	Q15596
Lonicerae Japonicae Flos	MOL001285	octanol	Protein kinase C beta type	PRKCB	P05771
Lonicerae Japonicae Flos	MOL000130	CAM	Muscarinic acetylcholine receptor M1	CHRM1	P11229
Lonicerae Japonicae Flos	MOL000130	CAM	Muscarinic acetylcholine receptor M2	CHRM2	P08172
Lonicerae Japonicae Flos	MOL001300	PEL	Alcohol dehydrogenase 1B	ADH1B	P00325
Lonicerae Japonicae Flos	MOL001300	PEL	Alcohol dehydrogenase 1C	ADH1C	P00326
Lonicerae Japonicae Flos	MOL001300	PEL	Trypsin-3	PRSS3	P35030
Lonicerae Japonicae Flos	MOL001300	PEL	Alcohol dehydrogenase 1A	ADH1A	P07327
Lonicerae Japonicae Flos	MOL001300	PEL	Amine oxidase [flavin-containing] A	MAOA	P21397
Lonicerae Japonicae Flos	MOL001388	(+)-Ledol	Muscarinic acetylcholine receptor M3	CHRM3	P20309

Lonicerae Japonicae Flos	MOL001388	(+)-Ledol	Muscarinic acetylcholine receptor M1	CHRM1	P11229
Lonicerae Japonicae Flos	MOL001388	(+)-Ledol	Muscarinic acetylcholine receptor M2	CHRM2	P08172
Lonicerae Japonicae Flos	MOL001388	(+)-Ledol	Gamma-aminobutyric acid receptor subunit alpha-1	GABRA1	P14867
Lonicerae Japonicae Flos	MOL001388	(+)-Ledol	Alpha-1B adrenergic receptor	ADRA1B	P35368
Lonicerae Japonicae Flos	MOL001393	myristic acid	Prostaglandin G/H synthase 1	PTGS1	P23219
Lonicerae Japonicae Flos	MOL001393	myristic acid	Prostaglandin G/H synthase 2	PTGS2	P35354
Lonicerae Japonicae Flos	MOL001393	myristic acid	Cholinesterase	BCHE	P06276
Lonicerae Japonicae Flos	MOL001393	myristic acid	Phospholipase A2	PLA2G1B	P04054
Lonicerae Japonicae Flos	MOL001393	myristic acid	Ig gamma-1 chain C region	IGHG1	P01857
Lonicerae Japonicae Flos	MOL001393	myristic acid	Nuclear receptor coactivator 2	NCOA2	Q15596
Lonicerae Japonicae Flos	MOL001393	myristic acid	Nuclear receptor coactivator 1	NCOA1	Q15788
Lonicerae Japonicae Flos	MOL001393	myristic acid	Phosphatidylcholine-sterol acyltransferase	LCAT	P04180
Lonicerae Japonicae Flos	MOL000511	ursolic acid	Urokinase-type plasminogen activator	PLAU	P00749
Lonicerae Japonicae Flos	MOL000511	ursolic acid	Cathepsin B	CTSB	P07858
Lonicerae Japonicae Flos	MOL000511	ursolic acid	Transcription factor p65	RELA	Q04206
Lonicerae Japonicae Flos	MOL000511	ursolic acid	Signal transducer and activator of transcription 3	STAT3	P40763
Lonicerae Japonicae Flos	MOL000511	ursolic acid	Vascular endothelial growth factor A	VEGFA	P15692
Lonicerae Japonicae Flos	MOL000511	ursolic acid	G1/S-specific cyclin-D1	CCND1	P24385
Lonicerae Japonicae Flos	MOL000511	ursolic acid	Apoptosis regulator Bcl-2	BCL2	P10415
Lonicerae Japonicae Flos	MOL000511	ursolic acid	Bcl-2-like protein 1	BCL2L1	Q07817
Lonicerae Japonicae Flos	MOL000511	ursolic acid	Proto-oncogene c-Fos	FOS	P01100
Lonicerae Japonicae Flos	MOL000511	ursolic acid	Cyclin-dependent kinase inhibitor 1	CDKN1A	P38936
Lonicerae Japonicae Flos	MOL000511	ursolic acid	Apoptosis regulator BAX	BAX	Q07812
Lonicerae Japonicae Flos	MOL000511	ursolic acid	Caspase-9	CASP9	P55211
Lonicerae Japonicae Flos	MOL000511	ursolic acid	72 kDa type IV collagenase	MMP2	P08253
Lonicerae Japonicae Flos	MOL000511	ursolic acid	Matrix metalloproteinase-9	MMP9	P14780
Lonicerae Japonicae Flos	MOL000511	ursolic acid	Cell division protein kinase 4	CDK4	P11802
Lonicerae Japonicae Flos	MOL000511	ursolic acid	Tumor necrosis factor	TNFAIP6	P98066
Lonicerae Japonicae Flos	MOL000511	ursolic acid	Transcription factor AP-1	JUN	P05412
Lonicerae Japonicae Flos	MOL000511	ursolic acid	Interleukin-6	IL6	P05231
Lonicerae Japonicae Flos	MOL000511	ursolic acid	Cell division protein kinase 6	CDK6	Q00534
Lonicerae Japonicae Flos	MOL000511	ursolic acid	Caspase-3	CASP3	P42574
Lonicerae Japonicae Flos	MOL000511	ursolic acid	Cellular tumor antigen p53	TP53	P04637
Lonicerae Japonicae Flos	MOL000511	ursolic acid	Mitogen-activated protein kinase 8	MAPK8	P45983
Lonicerae Japonicae Flos	MOL000511	ursolic acid	Prostaglandin G/H synthase 2	PTGS2	P35354
Lonicerae Japonicae Flos	MOL000511	ursolic acid	NF-kappa-B inhibitor alpha	NFKBIA	P25963
Lonicerae Japonicae Flos	MOL000511	ursolic acid	Caspase-8	CASP8	Q14790
Lonicerae Japonicae Flos	MOL000511	ursolic acid	Fatty acid synthase	FASN	P49327
Lonicerae Japonicae Flos	MOL000511	ursolic acid	Interstitial collagenase	MMP1	P03956
Lonicerae Japonicae Flos	MOL000511	ursolic acid	Stromelysin-1	MMP3	P08254
Lonicerae Japonicae Flos	MOL000511	ursolic acid	Heparin-binding growth factor 2	FGF2	P09038
Lonicerae Japonicae Flos	MOL000511	ursolic acid	Stromelysin-2	MMP10	P09238
Lonicerae Japonicae Flos	MOL000511	ursolic acid	Intercellular adhesion molecule 1	ICAM1	P05362
Lonicerae Japonicae Flos	MOL000511	ursolic acid	Interleukin-1 beta	IL1B	P01584

Lonicerae Japonicae Flos	MOL000511	ursolic acid	Cyclic AMP-responsive element-binding protein 1	CREB1	P16220
Lonicerae Japonicae Flos	MOL000511	ursolic acid	E-selectin	SELE	P16581
Lonicerae Japonicae Flos	MOL000511	ursolic acid	Prostaglandin E2 receptor EP3 subtype	PTGER3	P43115
Lonicerae Japonicae Flos	MOL000511	ursolic acid	Prostaglandin G/H synthase 1	PTGS1	P23219
Lonicerae Japonicae Flos	MOL000511	ursolic acid	Induced myeloid leukemia cell differentiation protein Mcl-1	MCL1	Q07820
Lonicerae Japonicae Flos	MOL000511	ursolic acid	Protein kinase C gamma type	PRKCG	P05129
Lonicerae Japonicae Flos	MOL000511	ursolic acid	Cyclic AMP-dependent transcription factor ATF-2	ATF2	P15336
Lonicerae Japonicae Flos	MOL000511	ursolic acid	Granulocyte-macrophage colony-stimulating factor	CSF2	P04141
Lonicerae Japonicae Flos	MOL000511	ursolic acid	Platelet endothelial cell adhesion molecule	PECAM1	P16284
Lonicerae Japonicae Flos	MOL000511	ursolic acid	C-Jun-amino-terminal kinase-interacting protein 2	MAPK8IP2	Q13387
Lonicerae Japonicae Flos	MOL000511	ursolic acid	Baculoviral IAP repeat-containing protein 5	BIRC5	O15392
Lonicerae Japonicae Flos	MOL000511	ursolic acid	Tyrosine-protein phosphatase non-receptor type 6	PTPN6	P29350
Lonicerae Japonicae Flos	MOL000511	ursolic acid	Neuromodulin	GAP43	P17677
Lonicerae Japonicae Flos	MOL000511	ursolic acid	Dual oxidase 2	DUOX2	Q9NRD8
Lonicerae Japonicae Flos	MOL000511	ursolic acid	Nitric oxide synthase, endothelial	NOS3	P29474
Lonicerae Japonicae Flos	MOL000511	ursolic acid	Tyrosine-protein phosphatase non-receptor type 1	PTPN1	P18031
Lonicerae Japonicae Flos	MOL000511	ursolic acid	ipopolysaccharide-induced tumor necrosis factor-alpha facto	LITAF	Q99732
Lonicerae Japonicae Flos	MOL000511	ursolic acid	G1/S-specific cyclin-D2	CCND2	P30279
Lonicerae Japonicae Flos	MOL000511	ursolic acid	Tumor necrosis factor ligand superfamily member 6	FASLG	P48023
Lonicerae Japonicae Flos	MOL000511	ursolic acid	Caspase-1	CASP1	P29466
Lonicerae Japonicae Flos	MOL000511	ursolic acid	nucleotide pyrophosphatase/phosphodiesterase family meml	ENPP7	Q6UWV6
Lonicerae Japonicae Flos	MOL001494	Mandenol	Prostaglandin G/H synthase 1	PTGS1	P23219
Lonicerae Japonicae Flos	MOL001494	Mandenol	Prostaglandin G/H synthase 2	PTGS2	P35354
Lonicerae Japonicae Flos	MOL001494	Mandenol	Nuclear receptor coactivator 2	NCOA2	Q15596
Lonicerae Japonicae Flos	MOL001495	Ethyl linolenate	Prostaglandin G/H synthase 1	PTGS1	P23219
Lonicerae Japonicae Flos	MOL001495	Ethyl linolenate	Nuclear receptor coactivator 2	NCOA2	Q15596
Lonicerae Japonicae Flos	MOL001600	copaene	Prostaglandin G/H synthase 2	PTGS2	P35354
Lonicerae Japonicae Flos	MOL001600	copaene	Gamma-aminobutyric acid receptor subunit alpha-1	GABRA1	P14867
Lonicerae Japonicae Flos	MOL001600	copaene	Muscarinic acetylcholine receptor M3	CHRM3	P20309
Lonicerae Japonicae Flos	MOL001600	copaene	Muscarinic acetylcholine receptor M1	CHRM1	P11229
Lonicerae Japonicae Flos	MOL001600	copaene	Retinoic acid receptor RXR-alpha	RXRA	P19793
Lonicerae Japonicae Flos	MOL001600	copaene	Muscarinic acetylcholine receptor M2	CHRM2	P08172
Lonicerae Japonicae Flos	MOL001600	copaene	Alpha-1B adrenergic receptor	ADRA1B	P35368
Lonicerae Japonicae Flos	MOL001604	Linalool	Muscarinic acetylcholine receptor M3	CHRM3	P20309
Lonicerae Japonicae Flos	MOL001604	Linalool	Muscarinic acetylcholine receptor M1	CHRM1	P11229
Lonicerae Japonicae Flos	MOL001604	Linalool	Muscarinic acetylcholine receptor M2	CHRM2	P08172
Lonicerae Japonicae Flos	MOL001604	Linalool	Gamma-aminobutyric acid receptor subunit alpha-1	GABRA1	P14867
Lonicerae Japonicae Flos	MOL001640	NON	Phospholipase A2	PLA2G1B	P04054
Lonicerae Japonicae Flos	MOL001640	NON	Alcohol dehydrogenase 1B	ADH1B	P00325
Lonicerae Japonicae Flos	MOL001640	NON	Alcohol dehydrogenase 1C	ADH1C	P00326
Lonicerae Japonicae Flos	MOL001640	NON	Ig gamma-1 chain C region	IGHG1	P01857
Lonicerae Japonicae Flos	MOL001719	2-[(2S,5R)-5-ethenyl-5-methyloxolan-2-yl]propan-2-ol	Muscarinic acetylcholine receptor M3	CHRM3	P20309
Lonicerae Japonicae Flos	MOL001719	2-[(2S,5R)-5-ethenyl-5-methyloxolan-2-yl]propan-2-ol	Muscarinic acetylcholine receptor M1	CHRM1	P11229
Lonicerae Japonicae Flos	MOL001719	2-[(2S,5R)-5-ethenyl-5-methyloxolan-2-yl]propan-2-ol	Muscarinic acetylcholine receptor M2	CHRM2	P08172

Lonicerae Japonicae Flos	MOL001719	2-[(2S,5R)-5-ethenyl-5-methyloxolan-2-yl]propan-2-ol	Alpha-1B adrenergic receptor	ADRA1B	P35368
Lonicerae Japonicae Flos	MOL001719	2-[(2S,5R)-5-ethenyl-5-methyloxolan-2-yl]propan-2-ol	Neuronal acetylcholine receptor subunit alpha-2	CHRNA2	Q15822
Lonicerae Japonicae Flos	MOL001719	2-[(2S,5R)-5-ethenyl-5-methyloxolan-2-yl]propan-2-ol	Gamma-aminobutyric acid receptor subunit alpha-1	GABRA1	P14867
Lonicerae Japonicae Flos	MOL001719	2-[(2S,5R)-5-ethenyl-5-methyloxolan-2-yl]propan-2-ol	Ig gamma-1 chain C region	IGHG1	P01857
Lonicerae Japonicae Flos	MOL001719	2-[(2S,5R)-5-ethenyl-5-methyloxolan-2-yl]propan-2-ol	Ig gamma-2 chain C region	IGHG2	P01859
Lonicerae Japonicae Flos	MOL000172	Furol	Alcohol dehydrogenase 1B	ADH1B	P00325
Lonicerae Japonicae Flos	MOL000172	Furol	Alcohol dehydrogenase 1C	ADH1C	P00326
Lonicerae Japonicae Flos	MOL000172	Furol	Alcohol dehydrogenase 1A	ADH1A	P07327
Lonicerae Japonicae Flos	MOL000172	Furol	Lysozyme	LYZ	P61626
Lonicerae Japonicae Flos	MOL001773	indole	Alcohol dehydrogenase 1C	ADH1C	P00326
Lonicerae Japonicae Flos	MOL001773	indole	Lysozyme	LYZ	P61626
Lonicerae Japonicae Flos	MOL001773	indole	Interferon gamma	IFNG	P01579
Lonicerae Japonicae Flos	MOL001773	indole	Glutamate--cysteine ligase catalytic subunit	GCLC	P48506
Lonicerae Japonicae Flos	MOL000019	D-Camphene	Gamma-aminobutyric acid receptor subunit alpha-1	GABRA1	P14867
Lonicerae Japonicae Flos	MOL000019	D-Camphene	Potassium voltage-gated channel subfamily H member 2	KCNH2	Q12809
Lonicerae Japonicae Flos	MOL000019	D-Camphene	Sodium channel protein type 5 subunit alpha	SCN5A	Q14524
Lonicerae Japonicae Flos	MOL000019	D-Camphene	Prostaglandin G/H synthase 2	PTGS2	P35354
Lonicerae Japonicae Flos	MOL000019	D-Camphene	Nuclear receptor coactivator 2	NCOA2	Q15596
Lonicerae Japonicae Flos	MOL000019	D-Camphene	Calmodulin	CAMSAP2	Q08AD1
Lonicerae Japonicae Flos	MOL000196	L-Bornyl acetate	Muscarinic acetylcholine receptor M3	CHRM3	P20309
Lonicerae Japonicae Flos	MOL000196	L-Bornyl acetate	Muscarinic acetylcholine receptor M1	CHRM1	P11229
Lonicerae Japonicae Flos	MOL000196	L-Bornyl acetate	Muscarinic acetylcholine receptor M2	CHRM2	P08172
Lonicerae Japonicae Flos	MOL000196	L-Bornyl acetate	Alpha-1B adrenergic receptor	ADRA1B	P35368
Lonicerae Japonicae Flos	MOL000196	L-Bornyl acetate	Gamma-aminobutyric acid receptor subunit alpha-1	GABRA1	P14867
Lonicerae Japonicae Flos	MOL000196	L-Bornyl acetate	Glutamate receptor 2	GRIA2	P42262
Lonicerae Japonicae Flos	MOL000196	L-Bornyl acetate	Prostaglandin G/H synthase 2	PTGS2	P35354
Lonicerae Japonicae Flos	MOL000196	L-Bornyl acetate	Neuronal acetylcholine receptor subunit alpha-2	CHRNA2	Q15822
Lonicerae Japonicae Flos	MOL000196	L-Bornyl acetate	Dopamine D1 receptor	DRD1	P21728
Lonicerae Japonicae Flos	MOL000196	L-Bornyl acetate	Alpha-2A adrenergic receptor	ADRA2A	P08913
Lonicerae Japonicae Flos	MOL000196	L-Bornyl acetate	Sodium-dependent noradrenaline transporter	SLC6A2	P23975
Lonicerae Japonicae Flos	MOL002042	thymol	Prostaglandin G/H synthase 1	PTGS1	P23219
Lonicerae Japonicae Flos	MOL002042	thymol	Muscarinic acetylcholine receptor M1	CHRM1	P11229
Lonicerae Japonicae Flos	MOL002042	thymol	Beta-1 adrenergic receptor	ADRB1	P08588
Lonicerae Japonicae Flos	MOL002042	thymol	Prostaglandin G/H synthase 2	PTGS2	P35354
Lonicerae Japonicae Flos	MOL002042	thymol	Alpha-2C adrenergic receptor	ADRA2C	P18825
Lonicerae Japonicae Flos	MOL002042	thymol	Sodium-dependent noradrenaline transporter	SLC6A2	P23975
Lonicerae Japonicae Flos	MOL002042	thymol	Alpha-1A adrenergic receptor	ADRA1A	P35348
Lonicerae Japonicae Flos	MOL002042	thymol	Sodium-dependent dopamine transporter	SLC6A3	Q01959
Lonicerae Japonicae Flos	MOL002042	thymol	Beta-2 adrenergic receptor	ADRB2	P07550
Lonicerae Japonicae Flos	MOL002042	thymol	Lysozyme	LYZ	P61626
Lonicerae Japonicae Flos	MOL002042	thymol	Muscarinic acetylcholine receptor M3	CHRM3	P20309
Lonicerae Japonicae Flos	MOL002042	thymol	Muscarinic acetylcholine receptor M2	CHRM2	P08172
Lonicerae Japonicae Flos	MOL002042	thymol	Alpha-1B adrenergic receptor	ADRA1B	P35368
Lonicerae Japonicae Flos	MOL002042	thymol	Alpha-1D adrenergic receptor	ADRA1D	P25100

Lonicerae Japonicae Flos	MOL002042	thymol	Neutrophil elastase	ELANE	P08246
Lonicerae Japonicae Flos	MOL000009	luteolin-7-o-glucoside	Trypsin-1	PRSS1	P07477
Lonicerae Japonicae Flos	MOL000009	luteolin-7-o-glucoside	Nitric oxide synthase, inducible	NOS2	P35228
Lonicerae Japonicae Flos	MOL000009	luteolin-7-o-glucoside	Prostaglandin G/H synthase 2	PTGS2	P35354
Lonicerae Japonicae Flos	MOL002083	tricin	Nitric oxide synthase, inducible	NOS2	P35228
Lonicerae Japonicae Flos	MOL002083	tricin	Estrogen receptor	ESR1	P03372
Lonicerae Japonicae Flos	MOL002083	tricin	Androgen receptor	AR	P10275
Lonicerae Japonicae Flos	MOL002083	tricin	Peroxisome proliferator activated receptor gamma	PPARG	P37231
Lonicerae Japonicae Flos	MOL002083	tricin	Prostaglandin G/H synthase 2	PTGS2	P35354
Lonicerae Japonicae Flos	MOL002083	tricin	Coagulation factor VII	F7	P08709
Lonicerae Japonicae Flos	MOL002083	tricin	Estrogen receptor beta	ESR2	Q92731
Lonicerae Japonicae Flos	MOL002083	tricin	Dipeptidyl peptidase IV	DPP4	P27487
Lonicerae Japonicae Flos	MOL002083	tricin	Mitogen-activated protein kinase 14	MAPK14	Q16539
Lonicerae Japonicae Flos	MOL002083	tricin	Glycogen synthase kinase-3 beta	GSK3B	P49841
Lonicerae Japonicae Flos	MOL002083	tricin	Heat shock protein HSP 90	HSP90AB1	P08238
Lonicerae Japonicae Flos	MOL002083	tricin	Cell division protein kinase 2	CDK2	P24941
Lonicerae Japonicae Flos	MOL002083	tricin	Trypsin-1	PRSS1	P07477
Lonicerae Japonicae Flos	MOL002083	tricin	Nuclear receptor coactivator 2	NCOA2	Q15596
Lonicerae Japonicae Flos	MOL002083	tricin	Calmodulin	CAMSAP2	Q08AD1
Lonicerae Japonicae Flos	MOL002083	tricin	Prostaglandin G/H synthase 1	PTGS1	P23219
Lonicerae Japonicae Flos	MOL002083	tricin	Sodium channel protein type 5 subunit alpha	SCN5A	Q14524
Lonicerae Japonicae Flos	MOL002083	tricin	Estrogen sulfotransferase	SULT1E1	P49888
Lonicerae Japonicae Flos	MOL002085	alpha-Cubebene	Muscarinic acetylcholine receptor M3	CHRM3	P20309
Lonicerae Japonicae Flos	MOL002085	alpha-Cubebene	Muscarinic acetylcholine receptor M1	CHRM1	P11229
Lonicerae Japonicae Flos	MOL002085	alpha-Cubebene	Prostaglandin G/H synthase 2	PTGS2	P35354
Lonicerae Japonicae Flos	MOL002085	alpha-Cubebene	Muscarinic acetylcholine receptor M2	CHRM2	P08172
Lonicerae Japonicae Flos	MOL002085	alpha-Cubebene	Neuronal acetylcholine receptor subunit alpha-2	CHRNA2	Q15822
Lonicerae Japonicae Flos	MOL002085	alpha-Cubebene	Gamma-aminobutyric acid receptor subunit alpha-1	GABRA1	P14867
Lonicerae Japonicae Flos	MOL002121	(1S,4E,8E,10R)-4,8,11,11-tetramethylbicyclo[8.1.0]undeca-4,8-diene	Prostaglandin G/H synthase 1	PTGS1	P23219
Lonicerae Japonicae Flos	MOL002121	(1S,4E,8E,10R)-4,8,11,11-tetramethylbicyclo[8.1.0]undeca-4,8-diene	Prostaglandin G/H synthase 2	PTGS2	P35354
Lonicerae Japonicae Flos	MOL002121	(1S,4E,8E,10R)-4,8,11,11-tetramethylbicyclo[8.1.0]undeca-4,8-diene	Muscarinic acetylcholine receptor M2	CHRM2	P08172
Lonicerae Japonicae Flos	MOL002121	(1S,4E,8E,10R)-4,8,11,11-tetramethylbicyclo[8.1.0]undeca-4,8-diene	Gamma-aminobutyric acid receptor subunit alpha-1	GABRA1	P14867
Lonicerae Japonicae Flos	MOL002121	(1S,4E,8E,10R)-4,8,11,11-tetramethylbicyclo[8.1.0]undeca-4,8-diene	Nuclear receptor coactivator 2	NCOA2	Q15596
Lonicerae Japonicae Flos	MOL000252	farnesol	Prostaglandin G/H synthase 2	PTGS2	P35354
Lonicerae Japonicae Flos	MOL000252	farnesol	Sodium-dependent noradrenaline transporter	SLC6A2	P23975
Lonicerae Japonicae Flos	MOL000252	farnesol	Amine oxidase [flavin-containing] B	MAOB	P27338
Lonicerae Japonicae Flos	MOL000252	farnesol	Nuclear receptor coactivator 2	NCOA2	Q15596
Lonicerae Japonicae Flos	MOL000252	farnesol	Prostaglandin G/H synthase 1	PTGS1	P23219
Lonicerae Japonicae Flos	MOL000252	farnesol	Retinoic acid receptor RXR-alpha	RXRA	P19793
Lonicerae Japonicae Flos	MOL000252	farnesol	Interleukin-6	IL6R	P08887
Lonicerae Japonicae Flos	MOL000252	farnesol	Caspase-3	CASP3	P42574
Lonicerae Japonicae Flos	MOL000252	farnesol	Involucrin	IVL	P07476
Lonicerae Japonicae Flos	MOL000252	farnesol	Ras-specific guanine nucleotide-releasing factor 2	RASGRF2	O14827
Lonicerae Japonicae Flos	MOL000252	farnesol	Bcl-2 homologous antagonist/killer	BAK1	Q16611

Lonicerae Japonicae Flos	MOL000252	farnesol	Toll-like receptor 4	TLR4	O00206
Lonicerae Japonicae Flos	MOL000252	farnesol	Lipoprotein lipase	LPL	P06858
Lonicerae Japonicae Flos	MOL000252	farnesol	3-hydroxy-3-methylglutaryl-coenzyme A reductase	HMGCR	P04035
Lonicerae Japonicae Flos	MOL000252	farnesol	Peroxisome proliferator-activated receptor alpha	PPARA	Q07869
Lonicerae Japonicae Flos	MOL000252	farnesol	Toll-like receptor 2	TLR2	O60603
Lonicerae Japonicae Flos	MOL000252	farnesol	Beta-defensin 2	DEFB4A	O15263
Lonicerae Japonicae Flos	MOL000252	farnesol	Protein HIRA	HIRA	P54198
Lonicerae Japonicae Flos	MOL000252	farnesol	SERTA domain-containing protein 3	SERTAD3	Q9UJW9
Lonicerae Japonicae Flos	MOL000252	farnesol	Progesterone receptor	PGR	P06401
Lonicerae Japonicae Flos	MOL000252	farnesol	Bile acid receptor	NR1H4	Q96RI1
Lonicerae Japonicae Flos	MOL000252	farnesol	Glutaminase liver isoform, mitochondrial	GLS2	Q9UI32
Lonicerae Japonicae Flos	MOL000252	farnesol	Ig delta chain C region	IGHD	P01880
Lonicerae Japonicae Flos	MOL002522	beta-Rhodinol	Alcohol dehydrogenase 1C	ADH1C	P00326
