# Supplementary material for: Network Pharmacology-Based Analysis on the Potential Biological Mechanisms of Yinzhihuang Oral Liquid in Treating Neonatal Hyperbilirubinemia
Source: Evid Based Complement Alternat Med. 2022 Oct 5;2022:1672670. doi: 10.1155/2022/1672670 (PMC9556251; doi:10.1155/2022/1672670)
Supplement: Supplementary Materials — Table S1: active herbal ingredients in Scutellariae Radix. Table S2: active herbal ingredients in Lonicerae Japonicae Flos. Table S3: active herbal ingredients in Artemisiae Scopariae Herba. Table S4: active herbal ingredients in Gardeniae Fructus. Table S5: ingredients in Scutellariae Radix and corresponding targets. Table S6: ingredients in Lonicerae Japonicae Flos and corresponding targets. Table S7: ingredients in Artemisiae Scopariae Herba and corresponding targets. Table S8: ingredients in Gardeniae Fructus and corresponding targets. Table S9: compound-common target network of YZH and neonatal hyperbilirubinemia. Table S10: PPI network into Cytoscape for YZH and neonatal hyperbilirubinemia analysis (minimum required interaction score of 0.9). Table S11: Gene Ontology (GO) Biological Process analysis (p < 0.05). [file 1672670.f1.zip › Table S7.pdf]

Table S7 Ingredients in Artemisiae Scopariae Herba and corresponding targets

Ingredients	MOL_ID	Molecule_Name	Protein name	Gene Name	Uniprot
Artemisiae Scopariae Herba	MOL000118	(L)-alpha-Terpineol	Muscarinic acetylcholine receptor M1	CHRM1	P11229
Artemisiae Scopariae Herba	MOL000118	(L)-alpha-Terpineol	Sodium-dependent noradrenaline transporter	SLC6A2	P23975
Artemisiae Scopariae Herba	MOL000118	(L)-alpha-Terpineol	Muscarinic acetylcholine receptor M2	CHRM2	P08172
Artemisiae Scopariae Herba	MOL000118	(L)-alpha-Terpineol	Alpha-1B adrenergic receptor	ADRA1B	P35368
Artemisiae Scopariae Herba	MOL000118	(L)-alpha-Terpineol	Sodium-dependent dopamine transporter	SLC6A3	Q01959
Artemisiae Scopariae Herba	MOL000118	(L)-alpha-Terpineol	Gamma-aminobutyric acid receptor subunit alpha-1	GABRA1	P14867
Artemisiae Scopariae Herba	MOL000118	(L)-alpha-Terpineol	Prostaglandin G/H synthase 1	PTGS1	P23219
Artemisiae Scopariae Herba	MOL000118	(L)-alpha-Terpineol	Sodium channel protein type 5 subunit alpha	SCN5A	Q14524
Artemisiae Scopariae Herba	MOL000118	(L)-alpha-Terpineol	Prostaglandin G/H synthase 2	PTGS2	P35354
Artemisiae Scopariae Herba	MOL000118	(L)-alpha-Terpineol	Heat shock protein HSP 90	HSP90AB1	P08238
Artemisiae Scopariae Herba	MOL000118	(L)-alpha-Terpineol	Nuclear receptor coactivator 2	NCOA2	Q15596
Artemisiae Scopariae Herba	MOL000118	(L)-alpha-Terpineol	Alpha-1A adrenergic receptor	ADRA1A	P35348
Artemisiae Scopariae Herba	MOL000118	(L)-alpha-Terpineol	Ig gamma-1 chain C region	IGHG1	P01857
Artemisiae Scopariae Herba	MOL000172	Furol	Alcohol dehydrogenase 1B	ADH1B	P00325
Artemisiae Scopariae Herba	MOL000172	Furol	Alcohol dehydrogenase 1C	ADH1C	P00326
Artemisiae Scopariae Herba	MOL000172	Furol	Alcohol dehydrogenase 1A	ADH1A	P07327
Artemisiae Scopariae Herba	MOL000172	Furol	Lysozyme	LYZ	P61626
Artemisiae Scopariae Herba	MOL001801	salicylic acid	Prostaglandin G/H synthase 1	PTGS1	P23219
Artemisiae Scopariae Herba	MOL001801	salicylic acid	Prostaglandin G/H synthase 2	PTGS2	P35354
Artemisiae Scopariae Herba	MOL001801	salicylic acid	Amine oxidase [flavin-containing] B	MAOB	P27338
Artemisiae Scopariae Herba	MOL001801	salicylic acid	Alcohol dehydrogenase 1B	ADH1B	P00325
Artemisiae Scopariae Herba	MOL001801	salicylic acid	Alcohol dehydrogenase 1C	ADH1C	P00326
Artemisiae Scopariae Herba	MOL001801	salicylic acid	Lysozyme	LYZ	P61626
Artemisiae Scopariae Herba	MOL001801	salicylic acid	Trypsin-3	PRSS3	P35030
Artemisiae Scopariae Herba	MOL001801	salicylic acid	Arachidonate 5-lipoxygenase	ALOX5	P09917
Artemisiae Scopariae Herba	MOL001801	salicylic acid	Alcohol dehydrogenase 1A	ADH1A	P07327
Artemisiae Scopariae Herba	MOL001801	salicylic acid	Chymotrypsinogen B	CTRB1	P17538
Artemisiae Scopariae Herba	MOL001801	salicylic acid	Transcription factor p65	RELA	Q04206
Artemisiae Scopariae Herba	MOL001801	salicylic acid	Fatty acid synthase	FASN	P49327
Artemisiae Scopariae Herba	MOL001801	salicylic acid	Superoxide dismutase [Cu-Zn]	SOD1	P00441
Artemisiae Scopariae Herba	MOL001801	salicylic acid	Catalase	CAT	P04040
Artemisiae Scopariae Herba	MOL001801	salicylic acid	Endothelin-1	EDNRA	P25101
Artemisiae Scopariae Herba	MOL001801	salicylic acid	Interleukin-4	IL4	P05112
Artemisiae Scopariae Herba	MOL001801	salicylic acid	Neutrophil cytosol factor 1	NCF1	P14598
Artemisiae Scopariae Herba	MOL001801	salicylic acid	Glutathione S-transferase P	GSTP1	P09211
Artemisiae Scopariae Herba	MOL001801	salicylic acid	Serum paraoxonase/arylesterase 1	PON1	P27169
Artemisiae Scopariae Herba	MOL001801	salicylic acid	Apolipoprotein A-I	APOA1	P02647

Artemisiae Scopariae Herba	MOL001801	salicylic acid	Plasminogen	PLG	P00747
Artemisiae Scopariae Herba	MOL001801	salicylic acid	Nuclear factor NF-kappa-B p105 subunit	NFKB1	P19838
Artemisiae Scopariae Herba	MOL001801	salicylic acid	Interferon beta	IFNB1	P01574
Artemisiae Scopariae Herba	MOL001801	salicylic acid	Ferritin, mitochondrial	FTMT	Q8N4E7
Artemisiae Scopariae Herba	MOL001801	salicylic acid	Aldo-keto reductase family 1 member C1	AKR1C1	Q04828
Artemisiae Scopariae Herba	MOL001801	salicylic acid	Thyroid peroxidase	TPO	P07202
Artemisiae Scopariae Herba	MOL001801	salicylic acid	Beta-glucuronidase	GUSB	P08236
Artemisiae Scopariae Herba	MOL001801	salicylic acid	Hairy/enhancer-of-split related with YRPW motif protein 1	HEY1	Q9Y5J3
Artemisiae Scopariae Herba	MOL001801	salicylic acid	Prolow-density lipoprotein receptor-related protein 1	LRP1	Q07954
Artemisiae Scopariae Herba	MOL001801	salicylic acid	Prostacyclin synthase	PTGIS	Q16647
Artemisiae Scopariae Herba	MOL001801	salicylic acid	Tyrosine aminotransferase	TAT	P17735
Artemisiae Scopariae Herba	MOL001801	salicylic acid	Golgi-associated plant pathogenesis-related protein 1	GLIPR2	Q9H4G4
Artemisiae Scopariae Herba	MOL001801	salicylic acid	Membrane primary amine oxidase	AOC3	Q16853
Artemisiae Scopariae Herba	MOL001801	salicylic acid	Telomeric repeat-binding factor 1	TERF1	P54274
Artemisiae Scopariae Herba	MOL001880	OXL	Prostaglandin G/H synthase 1	PTGS1	P23219
Artemisiae Scopariae Herba	MOL001880	OXL	Nitric-oxide synthase, brain	NOS1	P29475
Artemisiae Scopariae Herba	MOL001880	OXL	Procollagen-lysine,2-oxoglutarate 5-dioxygenase 1	PLOD1	Q02809
Artemisiae Scopariae Herba	MOL001880	OXL	Aldehyde dehydrogenase, mitochondrial	ALDH2	P05091
Artemisiae Scopariae Herba	MOL001880	OXL	Succinate semialdehyde dehydrogenase, mitochondrial	ALDH5A1	P51649
Artemisiae Scopariae Herba	MOL001880	OXL	4-aminobutyrate aminotransferase, mitochondrial	ABAT	P80404
Artemisiae Scopariae Herba	MOL001880	OXL	Prostaglandin G/H synthase 2	PTGS2	P35354
Artemisiae Scopariae Herba	MOL001880	OXL	Serine hydroxymethyltransferase, mitochondrial	SHMT2	P34897
Artemisiae Scopariae Herba	MOL001880	OXL	DNA polymerase (HSV)		
Artemisiae Scopariae Herba	MOL001880	OXL	Glutamate [NMDA] receptor subunit zeta-1	GRIN1	Q05586
Artemisiae Scopariae Herba	MOL001880	OXL	Kynureninase	KYNU	Q16719
Artemisiae Scopariae Herba	MOL001880	OXL	Ornithine aminotransferase, mitochondrial	OAT	P04181
Artemisiae Scopariae Herba	MOL001880	OXL	Glycine receptor alpha-1 chain		
Artemisiae Scopariae Herba	MOL001880	OXL	Aspartate aminotransferase, cytoplasmic	GOT1	P17174
Artemisiae Scopariae Herba	MOL001880	OXL	Glutamate [NMDA] receptor subunit epsilon 1	GRIN2A	Q12879
Artemisiae Scopariae Herba	MOL001880	OXL	Gamma-aminobutyric acid receptor subunit alpha-1	GABRA1	P14867
Artemisiae Scopariae Herba	MOL001880	OXL	Cathepsin D	CTSD	P07339
Artemisiae Scopariae Herba	MOL001880	OXL	Lactotransferrin	LTF	P02788
Artemisiae Scopariae Herba	MOL001880	OXL	Macrophage metalloelastase	MMP12	P39900
Artemisiae Scopariae Herba	MOL001880	OXL	Leukotriene A-4 hydrolase	LTA4H	P09960
Artemisiae Scopariae Herba	MOL001880	OXL	M-phase inducer phosphatase 2	CDC25B	P30305
Artemisiae Scopariae Herba	MOL001880	OXL	Cholinesterase	BCHE	P06276
Artemisiae Scopariae Herba	MOL001880	OXL	Alcohol dehydrogenase 1B	ADH1B	P00325
Artemisiae Scopariae Herba	MOL001880	OXL	Alcohol dehydrogenase 1C	ADH1C	P00326
Artemisiae Scopariae Herba	MOL001880	OXL	Alcohol dehydrogenase 1A	ADH1A	P07327

Artemisiae Scopariae Herba	MOL001880	OXL	Catalase	CAT	P04040
Artemisiae Scopariae Herba	MOL001880	OXL	Ribonucleoside-diphosphate reductase large subunit	RRM1	P23921
Artemisiae Scopariae Herba	MOL001880	OXL	Monocarboxylate transporter 2	SLC16A7	O60669
Artemisiae Scopariae Herba	MOL001880	OXL	Beta-galactosidase	GLB1	P16278
Artemisiae Scopariae Herba	MOL001880	OXL	Acetyl-CoA acetyltransferase	ACAA2	P42765
Artemisiae Scopariae Herba	MOL001880	OXL	Methionine synthase	MTR	Q99707
Artemisiae Scopariae Herba	MOL001880	OXL	-dicarboxylate transport transcriptional regulatory protein dc	ALDH9A1	P49189
Artemisiae Scopariae Herba	MOL001880	OXL	Glutamate dehydrogenase 1, mitochondrial	GLUD1	P00367
Artemisiae Scopariae Herba	MOL001880	OXL	Aspartate aminotransferase, mitochondrial	GOT2	P00505
Artemisiae Scopariae Herba	MOL001880	OXL	Serine--pyruvate aminotransferase	AGXT	P21549
Artemisiae Scopariae Herba	MOL001880	OXL	Monocarboxylate transporter 7	SLC16A6	O15403
Artemisiae Scopariae Herba	MOL001880	OXL	Alanine aminotransferase 1	GPT	P24298
Artemisiae Scopariae Herba	MOL001880	OXL	Aldehyde dehydrogenase X, mitochondrial	ALDH1B1	P30837
Artemisiae Scopariae Herba	MOL001880	OXL	ate dehydrogenase [ubiquinone] flavoprotein subunit, mitoch	SDHA	P31040
Artemisiae Scopariae Herba	MOL001880	OXL	Cystathionine gamma-lyase	CTH	P32929
Artemisiae Scopariae Herba	MOL001880	OXL	Serine hydroxymethyltransferase, cytosolic	SHMT1	P34896
Artemisiae Scopariae Herba	MOL001880	OXL	Betaine--homocysteine S-methyltransferase 1	BHMT	Q93088
Artemisiae Scopariae Herba	MOL001880	OXL	Alanine--glyoxylate aminotransferase 2, mitochondrial	AGXT2	Q9BYV1
Artemisiae Scopariae Herba	MOL001880	OXL	NADP-dependent malic enzyme	ME1	Q16798
Artemisiae Scopariae Herba	MOL001880	OXL	Glutamate dehydrogenase 2, mitochondrial	GLUD2	P49448
Artemisiae Scopariae Herba	MOL001880	OXL	Glycine amidinotransferase, mitochondrial	GATM	P50440
Artemisiae Scopariae Herba	MOL001880	OXL	Calcium-transporting ATPase type 2C member 1	ATP2C1	P98194
Artemisiae Scopariae Herba	MOL001880	OXL	Cysteine desulfurase, mitochondrial	NFS1	Q9Y697
Artemisiae Scopariae Herba	MOL001880	OXL	Alanine--glyoxylate aminotransferase 2-like 2	PHYKPL	Q8IUZ5
Artemisiae Scopariae Herba	MOL001880	OXL	Proton-coupled amino acid transporter 1	SLC36A1	Q7Z2H8
Artemisiae Scopariae Herba	MOL001880	OXL	Adenylosuccinate synthetase isozyme 1	ADSS1	Q8N142
Artemisiae Scopariae Herba	MOL001880	OXL	L-lactate dehydrogenase A chain	LDHA	P00338
Artemisiae Scopariae Herba	MOL001880	OXL	Glycine receptor subunit alpha-2	GLRA2	P23416
Artemisiae Scopariae Herba	MOL001880	OXL	2-amino-3-ketobutyrate coenzyme A ligase, mitochondrial	GCAT	O75600
Artemisiae Scopariae Herba	MOL001880	OXL	uvate dehydrogenase E1 component subunit beta, mitochond	PDHB	P11177
Artemisiae Scopariae Herba	MOL001880	OXL	Triosephosphate isomerase	TPI1	P60174
Artemisiae Scopariae Herba	MOL001880	OXL	Delta-1-pyrroline-5-carboxylate dehydrogenase, mitochondria	ALDH4A1	P30038
Artemisiae Scopariae Herba	MOL001880	OXL	NAD-dependent malic enzyme, mitochondrial	ME2	P23368
Artemisiae Scopariae Herba	MOL001880	OXL	Glycine receptor subunit alpha-3	GLRA3	O75311
Artemisiae Scopariae Herba	MOL001880	OXL	NADP-dependent malic enzyme, mitochondrial	ME3	Q16798
Artemisiae Scopariae Herba	MOL001880	OXL	L-lactate dehydrogenase B chain	LDHB	P07195
Artemisiae Scopariae Herba	MOL001880	OXL	Trypsin-3	PRSS3	P35030
Artemisiae Scopariae Herba	MOL001880	OXL	Aldose reductase	AKR1B1	P15121
Artemisiae Scopariae Herba	MOL001880	OXL	Glycogen phosphorylase, muscle form	PYGM	P11217

Artemisiae Scopariae Herba	MOL001880	OXL	N-acetylneuraminate lyase	NPL	Q9BXD5
Artemisiae Scopariae Herba	MOL001880	OXL	Phosphate-binding protein pstS precursor	PTER	Q96BW5
Artemisiae Scopariae Herba	MOL001880	OXL	ADH-ubiquinone oxidoreductase 75 kDa subunit, mitochondr	NDUFS1	P28331
Artemisiae Scopariae Herba	MOL001880	OXL	Gephyrin	GPHN	Q9NQX3
Artemisiae Scopariae Herba	MOL001880	OXL	Prolyl 4-hydroxylase subunit alpha-2	P4HA2	O15460
Artemisiae Scopariae Herba	MOL001880	OXL	Calcium-binding mitochondrial carrier protein Aralar2	SLC25A13	Q9UJS0
Artemisiae Scopariae Herba	MOL001999	scoparone	Prostaglandin G/H synthase 1	PTGS1	P23219
Artemisiae Scopariae Herba	MOL001999	scoparone	Muscarinic acetylcholine receptor M1	CHRM1	P11229
Artemisiae Scopariae Herba	MOL001999	scoparone	Prostaglandin G/H synthase 2	PTGS2	P35354
Artemisiae Scopariae Herba	MOL001999	scoparone	Beta-2 adrenergic receptor	ADRB2	P07550
Artemisiae Scopariae Herba	MOL001999	scoparone	Sodium-dependent serotonin transporter	SLC6A4	P31645
Artemisiae Scopariae Herba	MOL001999	scoparone	Gamma-aminobutyric acid receptor subunit alpha-1	GABRA1	P14867
Artemisiae Scopariae Herba	MOL001999	scoparone	Leukotriene A-4 hydrolase	LTA4H	P09960
Artemisiae Scopariae Herba	MOL001999	scoparone	Amine oxidase [flavin-containing] B	MAOB	P27338
Artemisiae Scopariae Herba	MOL001999	scoparone	Glutamate receptor 2	GRIA2	P42262
Artemisiae Scopariae Herba	MOL001999	scoparone	cAMP-dependent protein kinase inhibitor alpha	PKIA	P61925
Artemisiae Scopariae Herba	MOL001999	scoparone	Transcription factor p65	RELA	Q04206
Artemisiae Scopariae Herba	MOL001999	scoparone	NF-kappa-B inhibitor alpha	NFKBIA	P25963
Artemisiae Scopariae Herba	MOL001999	scoparone	C-C motif chemokine 2	CCL2	P13500
Artemisiae Scopariae Herba	MOL001999	scoparone	Interleukin-8	CXCL8	P10145
Artemisiae Scopariae Herba	MOL001999	scoparone	Tyrosinase	TYR	P14679
Artemisiae Scopariae Herba	MOL001999	scoparone	5,6-dihydroxyindole-2-carboxylic acid oxidase	TYRP1	P17643
Artemisiae Scopariae Herba	MOL001999	scoparone	L-dopachrome tautomerase	DCT	P40126
Artemisiae Scopariae Herba	MOL000207	Methyleugenol	Prostaglandin G/H synthase 1	PTGS1	P23219
Artemisiae Scopariae Herba	MOL000207	Methyleugenol	Dopamine D1 receptor	DRD1	P21728
Artemisiae Scopariae Herba	MOL000207	Methyleugenol	Muscarinic acetylcholine receptor M3	CHRM3	P20309
Artemisiae Scopariae Herba	MOL000207	Methyleugenol	Muscarinic acetylcholine receptor M1	CHRM1	P11229
Artemisiae Scopariae Herba	MOL000207	Methyleugenol	Beta-1 adrenergic receptor	ADRB1	P08588
Artemisiae Scopariae Herba	MOL000207	Methyleugenol	Sodium channel protein type 5 subunit alpha	SCN5A	Q14524
Artemisiae Scopariae Herba	MOL000207	Methyleugenol	Prostaglandin G/H synthase 2	PTGS2	P35354
Artemisiae Scopariae Herba	MOL000207	Methyleugenol	Alpha-2A adrenergic receptor	ADRA2A	P08913
Artemisiae Scopariae Herba	MOL000207	Methyleugenol	Alpha-2C adrenergic receptor	ADRA2C	P18825
Artemisiae Scopariae Herba	MOL000207	Methyleugenol	Sodium-dependent noradrenaline transporter	SLC6A2	P23975
Artemisiae Scopariae Herba	MOL000207	Methyleugenol	Alpha-1A adrenergic receptor	ADRA1A	P35348
Artemisiae Scopariae Herba	MOL000207	Methyleugenol	Muscarinic acetylcholine receptor M2	CHRM2	P08172
Artemisiae Scopariae Herba	MOL000207	Methyleugenol	Alpha-2B adrenergic receptor	ADRA2B	P18089
Artemisiae Scopariae Herba	MOL000207	Methyleugenol	Alpha-1B adrenergic receptor	ADRA1B	P35368
Artemisiae Scopariae Herba	MOL000207	Methyleugenol	Sodium-dependent dopamine transporter	SLC6A3	Q01959
Artemisiae Scopariae Herba	MOL000207	Methyleugenol	Beta-2 adrenergic receptor	ADRB2	P07550

Artemisiae Scopariae Herba	MOL000207	Methyleugenol	Alpha-1D adrenergic receptor	ADRA1D	P25100
Artemisiae Scopariae Herba	MOL000207	Methyleugenol	Beta-lactamase	DPEP1	P16444
Artemisiae Scopariae Herba	MOL000207	Methyleugenol	Amine oxidase [flavin-containing] B	MAOB	P27338
Artemisiae Scopariae Herba	MOL000207	Methyleugenol	Leukotriene A-4 hydrolase	LTA4H	P09960
Artemisiae Scopariae Herba	MOL000207	Methyleugenol	Sodium-dependent serotonin transporter	SLC6A4	P31645
Artemisiae Scopariae Herba	MOL000207	Methyleugenol	Urokinase-type plasminogen activator	PLAU	P00749
Artemisiae Scopariae Herba	MOL000207	Methyleugenol	cAMP-dependent protein kinase inhibitor alpha	PKIA	P61925
Artemisiae Scopariae Herba	MOL000251	Rhamnocitrin	Nitric oxide synthase, inducible	NOS2	P35228
Artemisiae Scopariae Herba	MOL000251	Rhamnocitrin	Prostaglandin G/H synthase 1	PTGS1	P23219
Artemisiae Scopariae Herba	MOL000251	Rhamnocitrin	Estrogen receptor	ESR1	P03372
Artemisiae Scopariae Herba	MOL000251	Rhamnocitrin	Androgen receptor	AR	P10275
Artemisiae Scopariae Herba	MOL000251	Rhamnocitrin	Peroxisome proliferator activated receptor gamma	PPARG	P37231
Artemisiae Scopariae Herba	MOL000251	Rhamnocitrin	Prostaglandin G/H synthase 2	PTGS2	P35354
Artemisiae Scopariae Herba	MOL000251	Rhamnocitrin	Estrogen receptor beta	ESR2	Q92731
Artemisiae Scopariae Herba	MOL000251	Rhamnocitrin	Dipeptidyl peptidase IV	DPP4	P27487
Artemisiae Scopariae Herba	MOL000251	Rhamnocitrin	Mitogen-activated protein kinase 14	MAPK14	Q16539
Artemisiae Scopariae Herba	MOL000251	Rhamnocitrin	Glycogen synthase kinase-3 beta	GSK3B	P49841
Artemisiae Scopariae Herba	MOL000251	Rhamnocitrin	Heat shock protein HSP 90	HSP90AB1	P08238
Artemisiae Scopariae Herba	MOL000251	Rhamnocitrin	Cell division protein kinase 2	CDK2	P24941
Artemisiae Scopariae Herba	MOL000251	Rhamnocitrin	Serine/threonine-protein kinase Chk1	CHEK1	O14757
Artemisiae Scopariae Herba	MOL000251	Rhamnocitrin	Trypsin-1	PRSS1	P07477
Artemisiae Scopariae Herba	MOL000251	Rhamnocitrin	Calmodulin	CAMSAP2	Q08AD1
Artemisiae Scopariae Herba	MOL000251	Rhamnocitrin	Nuclear receptor coactivator 2	NCOA2	Q15596
Artemisiae Scopariae Herba	MOL000251	Rhamnocitrin	Aldose reductase	AKR1B1	P15121
Artemisiae Scopariae Herba	MOL000254	eugenol	Prostaglandin G/H synthase 1	PTGS1	P23219
Artemisiae Scopariae Herba	MOL000254	eugenol	Dopamine D1 receptor	DRD1	P21728
Artemisiae Scopariae Herba	MOL000254	eugenol	Muscarinic acetylcholine receptor M3	CHRM3	P20309
Artemisiae Scopariae Herba	MOL000254	eugenol	Muscarinic acetylcholine receptor M1	CHRM1	P11229
Artemisiae Scopariae Herba	MOL000254	eugenol	Beta-1 adrenergic receptor	ADRB1	P08588
Artemisiae Scopariae Herba	MOL000254	eugenol	Prostaglandin G/H synthase 2	PTGS2	P35354
Artemisiae Scopariae Herba	MOL000254	eugenol	Alpha-2A adrenergic receptor	ADRA2A	P08913
Artemisiae Scopariae Herba	MOL000254	eugenol	Alpha-2C adrenergic receptor	ADRA2C	P18825
Artemisiae Scopariae Herba	MOL000254	eugenol	Sodium-dependent noradrenaline transporter	SLC6A2	P23975
Artemisiae Scopariae Herba	MOL000254	eugenol	Alpha-1A adrenergic receptor	ADRA1A	P35348
Artemisiae Scopariae Herba	MOL000254	eugenol	Muscarinic acetylcholine receptor M2	CHRM2	P08172
Artemisiae Scopariae Herba	MOL000254	eugenol	Alpha-1B adrenergic receptor	ADRA1B	P35368
Artemisiae Scopariae Herba	MOL000254	eugenol	Sodium-dependent dopamine transporter	SLC6A3	Q01959
Artemisiae Scopariae Herba	MOL000254	eugenol	Beta-2 adrenergic receptor	ADRB2	P07550
Artemisiae Scopariae Herba	MOL000254	eugenol	Beta-lactamase	DPEP1	P16444

Artemisiae Scopariae Herba	MOL000254	eugenol	Amine oxidase [flavin-containing] B	MAOB	P27338
Artemisiae Scopariae Herba	MOL000254	eugenol	Amine oxidase [flavin-containing] A	MAOA	P21397
Artemisiae Scopariae Herba	MOL000254	eugenol	Lysozyme	LYZ	P61626
Artemisiae Scopariae Herba	MOL000254	eugenol	Chymotrypsinogen B	CTRB1	P17538
Artemisiae Scopariae Herba	MOL000254	eugenol	Alpha-1D adrenergic receptor	ADRA1D	P25100
Artemisiae Scopariae Herba	MOL000254	eugenol	Leukotriene A-4 hydrolase	LTA4H	P09960
Artemisiae Scopariae Herba	MOL000254	eugenol	Alpha-2B adrenergic receptor	ADRA2B	P18089
Artemisiae Scopariae Herba	MOL000254	eugenol	Urokinase-type plasminogen activator	PLAU	P00749
Artemisiae Scopariae Herba	MOL000254	eugenol	Sodium channel protein type 5 subunit alpha	SCN5A	Q14524
Artemisiae Scopariae Herba	MOL000254	eugenol	Transcription factor p65	RELA	Q04206
Artemisiae Scopariae Herba	MOL000254	eugenol	Cytochrome P450 1A1	CYP1A1	P04798
Artemisiae Scopariae Herba	MOL000254	eugenol	Cytochrome P450 1B1	CYP1B1	Q16678
Artemisiae Scopariae Herba	MOL000254	eugenol	Arachidonate 5-lipoxygenase	ALOX5	P09917
Artemisiae Scopariae Herba	MOL000254	eugenol	Aryl hydrocarbon receptor	AHR	P35869
Artemisiae Scopariae Herba	MOL000254	eugenol	Canalicular multispecific organic anion transporter 1	ABCC2	Q92887
Artemisiae Scopariae Herba	MOL000254	eugenol	T-lymphocyte activation antigen CD86	CD86	P42081
Artemisiae Scopariae Herba	MOL000254	eugenol	e/threonine-protein phosphatase 2B catalytic subunit alpha is	PPP3CA	Q08209
Artemisiae Scopariae Herba	MOL000254	eugenol	Pepsin A	PGA5	P0DJD9
Artemisiae Scopariae Herba	MOL000254	eugenol	Mucin-1	MUC1	P15941
Artemisiae Scopariae Herba	MOL000254	eugenol	Quinone oxidoreductase	CRYZ	Q08257
Artemisiae Scopariae Herba	MOL000254	eugenol	Calcium-transporting ATPase type 2C member 1	ATP2C1	P98194
Artemisiae Scopariae Herba	MOL000254	eugenol	Short transient receptor potential channel 3	TRPC3	Q13507
Artemisiae Scopariae Herba	MOL000254	eugenol	ansient receptor potential cation channel subfamily V membe	TRPV3	Q8NET8
Artemisiae Scopariae Herba	MOL000254	eugenol	Ecto-NOX disulfide-thiol exchanger 2	ENOX2	Q16206
Artemisiae Scopariae Herba	MOL002818	Piceol	Alcohol dehydrogenase 1C	ADH1C	P00326
Artemisiae Scopariae Herba	MOL002818	Piceol	Lysozyme	LYZ	P61626
Artemisiae Scopariae Herba	MOL002818	Piceol	Alcohol dehydrogenase 1B	ADH1B	P00325
Artemisiae Scopariae Herba	MOL000339	Isoscopoletin	Prostaglandin G/H synthase 1	PTGS1	PTGS1
Artemisiae Scopariae Herba	MOL000339	Isoscopoletin	Dopamine D1 receptor	DRD1	P21728
Artemisiae Scopariae Herba	MOL000339	Isoscopoletin	Muscarinic acetylcholine receptor M1	CHRM1	P11229
Artemisiae Scopariae Herba	MOL000339	Isoscopoletin	Prostaglandin G/H synthase 2	PTGS2	P35354
Artemisiae Scopariae Herba	MOL000339	Isoscopoletin	Beta-2 adrenergic receptor	ADRB2	P07550
Artemisiae Scopariae Herba	MOL000339	Isoscopoletin	Leukotriene A-4 hydrolase	LTA4H	P09960
Artemisiae Scopariae Herba	MOL000339	Isoscopoletin	Amine oxidase [flavin-containing] B	MAOB	P27338
Artemisiae Scopariae Herba	MOL000339	Isoscopoletin	Glutamate receptor 2	GRIA2	P42262
Artemisiae Scopariae Herba	MOL000339	Isoscopoletin	cAMP-dependent protein kinase inhibitor alpha	PKIA	P61925
Artemisiae Scopariae Herba	MOL000339	Isoscopoletin	NRH dehydrogenase [quinone] 2	NQO2	P16083
Artemisiae Scopariae Herba	MOL000354	isorhamnetin	Nitric oxide synthase, inducible	NOS2	P35228
Artemisiae Scopariae Herba	MOL000354	isorhamnetin	Prostaglandin G/H synthase 1	PTGS1	P23219

Artemisiae Scopariae Herba	MOL000354	isorhamnetin	Estrogen receptor	ESR1	P03372
Artemisiae Scopariae Herba	MOL000354	isorhamnetin	Androgen receptor	AR	P10275
Artemisiae Scopariae Herba	MOL000354	isorhamnetin	Peroxisome proliferator activated receptor gamma	PPARG	P37231
Artemisiae Scopariae Herba	MOL000354	isorhamnetin	Prostaglandin G/H synthase 2	PTGS2	P35354
Artemisiae Scopariae Herba	MOL000354	isorhamnetin	Estrogen receptor beta	ESR2	Q92731
Artemisiae Scopariae Herba	MOL000354	isorhamnetin	Dipeptidyl peptidase IV	DPP4	P27487
Artemisiae Scopariae Herba	MOL000354	isorhamnetin	Mitogen-activated protein kinase 14	MAPK14	Q16539
Artemisiae Scopariae Herba	MOL000354	isorhamnetin	Glycogen synthase kinase-3 beta	GSK3B	P49841
Artemisiae Scopariae Herba	MOL000354	isorhamnetin	Heat shock protein HSP 90	HSP90AB1	P08238
Artemisiae Scopariae Herba	MOL000354	isorhamnetin	Cell division protein kinase 2	CDK2	P24941
Artemisiae Scopariae Herba	MOL000354	isorhamnetin	Trypsin-1	PRSS1	P07477
Artemisiae Scopariae Herba	MOL000354	isorhamnetin	Cyclin-A2	CCNA2	P20248
Artemisiae Scopariae Herba	MOL000354	isorhamnetin	Nuclear receptor coactivator 2	NCOA2	Q15596
Artemisiae Scopariae Herba	MOL000354	isorhamnetin	Calmodulin	CAMSAP2	Q08AD1
Artemisiae Scopariae Herba	MOL000354	isorhamnetin	Glycogen phosphorylase, muscle form	PYGM	P11217
Artemisiae Scopariae Herba	MOL000354	isorhamnetin	Peroxisome proliferator activated receptor delta	PPARD	Q03181
Artemisiae Scopariae Herba	MOL000354	isorhamnetin	Serine/threonine-protein kinase Chk1	CHEK1	O14757
Artemisiae Scopariae Herba	MOL000354	isorhamnetin	Aldose reductase	AKR1B1	P15121
Artemisiae Scopariae Herba	MOL000354	isorhamnetin	Nuclear receptor coactivator 1	NCOA1	Q15788
Artemisiae Scopariae Herba	MOL000354	isorhamnetin	Coagulation factor VII	F7	P08709
Artemisiae Scopariae Herba	MOL000354	isorhamnetin	Acetylcholinesterase	COLQ	Q9Y215
Artemisiae Scopariae Herba	MOL000354	isorhamnetin	Gamma-aminobutyric acid receptor subunit alpha-1	GABRA1	P14867
Artemisiae Scopariae Herba	MOL000354	isorhamnetin	Amine oxidase [flavin-containing] B	MAOB	P27338
Artemisiae Scopariae Herba	MOL000354	isorhamnetin	Glutamate receptor 2	GRIA2	P42262
Artemisiae Scopariae Herba	MOL000354	isorhamnetin	Transcription factor p65	RELA	Q04206
Artemisiae Scopariae Herba	MOL000354	isorhamnetin	Neutrophil cytosol factor 1	NCF1	P14598
Artemisiae Scopariae Herba	MOL000354	isorhamnetin	Oxidized low-density lipoprotein receptor 1	OLR1	P78380
Artemisiae Scopariae Herba	MOL000358	beta-sitosterol	Progesterone receptor	PGR	P06401
Artemisiae Scopariae Herba	MOL000358	beta-sitosterol	Nuclear receptor coactivator 2	NCOA2	Q15596
Artemisiae Scopariae Herba	MOL000358	beta-sitosterol	Prostaglandin G/H synthase 1	PTGS1	P23219
Artemisiae Scopariae Herba	MOL000358	beta-sitosterol	Prostaglandin G/H synthase 2	PTGS2	P35354
Artemisiae Scopariae Herba	MOL000358	beta-sitosterol	Heat shock protein HSP 90	HSP90AB1	P08238
Artemisiae Scopariae Herba	MOL000358	beta-sitosterol	Potassium voltage-gated channel subfamily H member 2	KCNH2	Q12809
Artemisiae Scopariae Herba	MOL000358	beta-sitosterol	Dopamine D1 receptor	DRD1	P21728
Artemisiae Scopariae Herba	MOL000358	beta-sitosterol	Muscarinic acetylcholine receptor M3	CHRM3	P20309
Artemisiae Scopariae Herba	MOL000358	beta-sitosterol	Muscarinic acetylcholine receptor M1	CHRM1	P11229
Artemisiae Scopariae Herba	MOL000358	beta-sitosterol	Sodium channel protein type 5 subunit alpha	SCN5A	Q14524
Artemisiae Scopariae Herba	MOL000358	beta-sitosterol	Muscarinic acetylcholine receptor M4	CHRM4	P08173
Artemisiae Scopariae Herba	MOL000358	beta-sitosterol	Alpha-1A adrenergic receptor	ADRA1A	P25100

ᐱrtemisiae Scopariae Herba	MOL000358	beta-sitosterol	Muscarinic acetylcholine receptor M2	CHRM2	P08172
ᐱrtemisiae Scopariae Herba	MOL000358	beta-sitosterol	Alpha-1B adrenergic receptor	ADRA1B	P35368
ᐱrtemisiae Scopariae Herba	MOL000358	beta-sitosterol	Beta-2 adrenergic receptor	ADRB2	P07550
ᐱrtemisiae Scopariae Herba	MOL000358	beta-sitosterol	Neuronal acetylcholine receptor subunit alpha-2	CHRNA2	Q15822
ᐱrtemisiae Scopariae Herba	MOL000358	beta-sitosterol	Sodium-dependent serotonin transporter	SLC6A4	P31645
ᐱrtemisiae Scopariae Herba	MOL000358	beta-sitosterol	Mu-type opioid receptor	OPRM1	P35372
ᐱrtemisiae Scopariae Herba	MOL000358	beta-sitosterol	Gamma-aminobutyric acid receptor subunit alpha-1	GABRA1	P14867
ᐱrtemisiae Scopariae Herba	MOL000358	beta-sitosterol	Apoptosis regulator Bcl-2	BCL2	P10415
ᐱrtemisiae Scopariae Herba	MOL000358	beta-sitosterol	Apoptosis regulator BAX	BAX	Q07812
ᐱrtemisiae Scopariae Herba	MOL000358	beta-sitosterol	Caspase-9	CASP9	P55211
ᐱrtemisiae Scopariae Herba	MOL000358	beta-sitosterol	Transcription factor AP-1	JUN	P05412
ᐱrtemisiae Scopariae Herba	MOL000358	beta-sitosterol	Caspase-3	CASP3	P42574
ᐱrtemisiae Scopariae Herba	MOL000358	beta-sitosterol	Caspase-8	CASP8	Q14790
ᐱrtemisiae Scopariae Herba	MOL000358	beta-sitosterol	Protein kinase C alpha type	PRKCA	P17252
ᐱrtemisiae Scopariae Herba	MOL000358	beta-sitosterol	Serum paraoxonase/arylesterase 1	PON1	P27169
ᐱrtemisiae Scopariae Herba	MOL000358	beta-sitosterol	Microtubule-associated protein 2	MAP2	P11137
ᐱrtemisiae Scopariae Herba	MOL000040	Scopoletol	Prostaglandin G/H synthase 1	PTGS1	P23219
ᐱrtemisiae Scopariae Herba	MOL000040	Scopoletol	NRH dehydrogenase [quinone] 2	NQO2	P16083
ᐱrtemisiae Scopariae Herba	MOL000040	Scopoletol	Prostaglandin G/H synthase 2	PTGS2	P35354
ᐱrtemisiae Scopariae Herba	MOL000040	Scopoletol	Carbonic anhydrase I	CA1	P00915
ᐱrtemisiae Scopariae Herba	MOL000040	Scopoletol	Beta-2 adrenergic receptor	ADRB2	P07550
ᐱrtemisiae Scopariae Herba	MOL000040	Scopoletol	Leukotriene A-4 hydrolase	LTA4H	P09960
ᐱrtemisiae Scopariae Herba	MOL000040	Scopoletol	Amine oxidase [flavin-containing] B	MAOB	P27338
ᐱrtemisiae Scopariae Herba	MOL000040	Scopoletol	Glutamate receptor 2	GRIA2	P42262
ᐱrtemisiae Scopariae Herba	MOL000040	Scopoletol	Sodium-dependent noradrenaline transporter	SLC6A2	P23975
ᐱrtemisiae Scopariae Herba	MOL000415	rutin	Transcription factor p65	RELA	Q04206
ᐱrtemisiae Scopariae Herba	MOL000415	rutin	Tumor necrosis factor	TNFAIP6	P98066
ᐱrtemisiae Scopariae Herba	MOL000415	rutin	Interleukin-6	IL6R	P08887
ᐱrtemisiae Scopariae Herba	MOL000415	rutin	Caspase-3	CASP3	P42574
ᐱrtemisiae Scopariae Herba	MOL000415	rutin	NADPH--cytochrome P450 reductase	POR	P16435
ᐱrtemisiae Scopariae Herba	MOL000415	rutin	Superoxide dismutase [Cu-Zn]	SOD1	P08294
ᐱrtemisiae Scopariae Herba	MOL000415	rutin	Catalase	CAT	P04040
ᐱrtemisiae Scopariae Herba	MOL000415	rutin	Interleukin-1 beta	IL1B	P01584
ᐱrtemisiae Scopariae Herba	MOL000415	rutin	Interleukin-8	CXCL8	P10145
ᐱrtemisiae Scopariae Herba	MOL000415	rutin	Protein kinase C beta type	PRKCB	P05771
ᐱrtemisiae Scopariae Herba	MOL000415	rutin	Arachidonate 5-lipoxygenase	ALOX5	P09917
ᐱrtemisiae Scopariae Herba	MOL000415	rutin	3-hydroxy-3-methylglutaryl-coenzyme A reductase	HMGCR	P04035
ᐱrtemisiae Scopariae Herba	MOL000415	rutin	Hyaluronan synthase 2	HAS2	Q92819
ᐱrtemisiae Scopariae Herba	MOL000415	rutin	Glutathione S-transferase P	GSTP1	P09211

Artemisiae Scopariae Herba	MOL000415	rutin	Type I iodothyronine deiodinase	DIO1	P49895
Artemisiae Scopariae Herba	MOL000415	rutin	C5a anaphylatoxin chemotactic receptor	C5AR1	Q9P296
Artemisiae Scopariae Herba	MOL000415	rutin	Insulin	TMEM219	Q86XT9
Artemisiae Scopariae Herba	MOL000415	rutin	Low affinity immunoglobulin epsilon Fc receptor	FCER2	P06734
Artemisiae Scopariae Herba	MOL000415	rutin	Integrin beta-2	ITGB2	P05107
Artemisiae Scopariae Herba	MOL000415	rutin	Thromboxane A2 receptor	TBXA2R	P21731
Artemisiae Scopariae Herba	MOL004609	Areapillin	Nitric oxide synthase, inducible	NOS2	P60321
Artemisiae Scopariae Herba	MOL004609	Areapillin	Androgen receptor	AR	P10275
Artemisiae Scopariae Herba	MOL004609	Areapillin	Sodium channel protein type 5 subunit alpha	SCN5A	Q14524
Artemisiae Scopariae Herba	MOL004609	Areapillin	Prostaglandin G/H synthase 2	PTGS2	P35354
Artemisiae Scopariae Herba	MOL004609	Areapillin	Coagulation factor VII	F7	P08709
Artemisiae Scopariae Herba	MOL004609	Areapillin	Estrogen receptor beta	ESR2	Q92731
Artemisiae Scopariae Herba	MOL004609	Areapillin	Dipeptidyl peptidase IV	DPP4	P27487
Artemisiae Scopariae Herba	MOL004609	Areapillin	Heat shock protein HSP 90	HSP90AB1	P08238
Artemisiae Scopariae Herba	MOL004609	Areapillin	Ig gamma-1 chain C region	IGHG1	P01857
Artemisiae Scopariae Herba	MOL004609	Areapillin	Trypsin-1	PRSS1	P07477
Artemisiae Scopariae Herba	MOL004609	Areapillin	Nuclear receptor coactivator 2	NCOA2	Q15596
Artemisiae Scopariae Herba	MOL004609	Areapillin	Nuclear receptor coactivator 1	NCOA1	Q15788
Artemisiae Scopariae Herba	MOL004609	Areapillin	Calmodulin	CAMSAP2	Q08AD1
