# Supplementary material for: Network Pharmacology-Based Analysis on the Potential Biological Mechanisms of Yinzhihuang Oral Liquid in Treating Neonatal Hyperbilirubinemia
Source: Evid Based Complement Alternat Med. 2022 Oct 5;2022:1672670. doi: 10.1155/2022/1672670 (PMC9556251; doi:10.1155/2022/1672670)
Supplement: Supplementary Materials — Table S1: active herbal ingredients in Scutellariae Radix. Table S2: active herbal ingredients in Lonicerae Japonicae Flos. Table S3: active herbal ingredients in Artemisiae Scopariae Herba. Table S4: active herbal ingredients in Gardeniae Fructus. Table S5: ingredients in Scutellariae Radix and corresponding targets. Table S6: ingredients in Lonicerae Japonicae Flos and corresponding targets. Table S7: ingredients in Artemisiae Scopariae Herba and corresponding targets. Table S8: ingredients in Gardeniae Fructus and corresponding targets. Table S9: compound-common target network of YZH and neonatal hyperbilirubinemia. Table S10: PPI network into Cytoscape for YZH and neonatal hyperbilirubinemia analysis (minimum required interaction score of 0.9). Table S11: Gene Ontology (GO) Biological Process analysis (p < 0.05). [file 1672670.f1.zip › Table S8.pdf]

Table S8 Ingredients in Gardeniae Fructus and corresponding targets

Ingredients	MOL_ID	Molecule_Name	Protein name	Gene Name	Uniprot
Gardeniae Fructus	MOL004557	geniposide	Carbonic anhydrase II	CA2	P00918
Gardeniae Fructus	MOL004557	geniposide	Apoptosis regulator Bcl-2	BCL2	P10415
Gardeniae Fructus	MOL004557	geniposide	Heme oxygenase 1	HMOX1	P09601
Gardeniae Fructus	MOL004557	geniposide	Neuromodulin	GAP43	P17677
Gardeniae Fructus	MOL004557	geniposide	Phospholipase B1, membrane-associated	PLB1	Q6P1J6
Gardeniae Fructus	MOL004557	geniposide	Glucagon	GLP1R	P01275
Gardeniae Fructus	MOL004557	geniposide	Glutathione S-transferase Mu 1	GSTM1	P09488
Gardeniae Fructus	MOL004557	geniposide	Glutathione S-transferase Mu 2	GSTM2	P28161
Gardeniae Fructus	MOL000415	rutin	DNA topoisomerase II	TOP2A	P11388
Gardeniae Fructus	MOL000415	rutin	Transcription factor p65	RELA	Q04206
Gardeniae Fructus	MOL000415	rutin	Tumor necrosis factor	TNFAIP6	P98066
Gardeniae Fructus	MOL000415	rutin	Interleukin-6	IL6R	P05231
Gardeniae Fructus	MOL000415	rutin	Caspase-3	CASP3	P42574
Gardeniae Fructus	MOL000415	rutin	NADPH--cytochrome P450 reductase	POR	P16435
Gardeniae Fructus	MOL000415	rutin	Superoxide dismutase [Cu-Zn]	SOD1	P00441
Gardeniae Fructus	MOL000415	rutin	Catalase	CAT	P04040
Gardeniae Fructus	MOL000415	rutin	Interleukin-1 beta	IL1B	P01584
Gardeniae Fructus	MOL000415	rutin	Interleukin-8	CXCL8	P10145
Gardeniae Fructus	MOL000415	rutin	Protein kinase C beta type	PRKCB	P05771
Gardeniae Fructus	MOL000415	rutin	Arachidonate 5-lipoxygenase	ALOX5	P09917
Gardeniae Fructus	MOL000415	rutin	3-hydroxy-3-methylglutaryl-coenzyme A reductase	HMGCR	P04035
Gardeniae Fructus	MOL000415	rutin	Hyaluronan synthase 2	HAS2	Q92819
Gardeniae Fructus	MOL000415	rutin	Glutathione S-transferase P	GSTP1	P09211
Gardeniae Fructus	MOL000415	rutin	Type I iodothyronine deiodinase	DIO1	P49895
Gardeniae Fructus	MOL000415	rutin	C5a anaphylatoxin chemotactic receptor	C5AR1	P21730
Gardeniae Fructus	MOL000415	rutin	Insulin	TMEM219	Q86XT9
Gardeniae Fructus	MOL000415	rutin	Low affinity immunoglobulin epsilon Fc receptor	FCER2	P06734
Gardeniae Fructus	MOL000415	rutin	Integrin beta-2	ITGB2	P05107
Gardeniae Fructus	MOL000415	rutin	Thromboxane A2 receptor	TBXA2R	P21731
Gardeniae Fructus	MOL004559	3,4,5-trihydroxy-6-(hydroxymethyl)oxan-2-yl]	Carbonic anhydrase II	CA2	P00918
Gardeniae Fructus	MOL004559	3,4,5-trihydroxy-6-(hydroxymethyl)oxan-2-yl]	Dipeptidyl peptidase IV	DPP4	P27487
Gardeniae Fructus	MOL004560	SHANZHISIDE_qt	Trypsin-1	PRSS1	P07477
Gardeniae Fructus	MOL004560	SHANZHISIDE_qt	Glutamate receptor 2	GRIA2	P42262
Gardeniae Fructus	MOL004561	Sudan III	Estrogen receptor	ESR1	P03372
Gardeniae Fructus	MOL004561	Sudan III	Prostaglandin G/H synthase 2	PTGS2	P35354
Gardeniae Fructus	MOL004561	Sudan III	Coagulation factor VII	F7	P08709
Gardeniae Fructus	MOL004561	Sudan III	Estrogen receptor beta	ESR2	Q92731

Gardeniae Fructus	MOL004561	Sudan III	Dipeptidyl peptidase IV	DPP4	P27487
Gardeniae Fructus	MOL004561	Sudan III	Mitogen-activated protein kinase 14	MAPK14	Q16539
Gardeniae Fructus	MOL004561	Sudan III	Glycogen synthase kinase-3 beta	GSK3B	P49841
Gardeniae Fructus	MOL004561	Sudan III	Mitogen-activated protein kinase 10	MAPK10	P53779
Gardeniae Fructus	MOL004561	Sudan III	Cell division protein kinase 2	CDK2	P24941
Gardeniae Fructus	MOL004561	Sudan III	Cyclin-A2	CCNA2	P20248
Gardeniae Fructus	MOL000511	ursolic acid	Urokinase-type plasminogen activator	PLAU	P00749
Gardeniae Fructus	MOL000511	ursolic acid	Cathepsin B	CTSB	P07858
Gardeniae Fructus	MOL000511	ursolic acid	Transcription factor p65	RELA	Q04206
Gardeniae Fructus	MOL000511	ursolic acid	Signal transducer and activator of transcription 3	STAT3	P40763
Gardeniae Fructus	MOL000511	ursolic acid	Vascular endothelial growth factor A	VEGFA	P15692
Gardeniae Fructus	MOL000511	ursolic acid	G1/S-specific cyclin-D1	CCND1	P24385
Gardeniae Fructus	MOL000511	ursolic acid	Apoptosis regulator Bcl-2	BCL2	P10415
Gardeniae Fructus	MOL000511	ursolic acid	Bcl-2-like protein 1	BCL2L1	Q07817
Gardeniae Fructus	MOL000511	ursolic acid	Proto-oncogene c-Fos	FOS	P01100
Gardeniae Fructus	MOL000511	ursolic acid	Cyclin-dependent kinase inhibitor 1	CDKN1A	P38936
Gardeniae Fructus	MOL000511	ursolic acid	Apoptosis regulator BAX	BAX	Q07812
Gardeniae Fructus	MOL000511	ursolic acid	Caspase-9	CASP9	P55211
Gardeniae Fructus	MOL000511	ursolic acid	72 kDa type IV collagenase	MMP2	P08253
Gardeniae Fructus	MOL000511	ursolic acid	Matrix metalloproteinase-9	MMP9	P14780
Gardeniae Fructus	MOL000511	ursolic acid	Cell division protein kinase 4	CDK4	P11802
Gardeniae Fructus	MOL000511	ursolic acid	Tumor necrosis factor	TNFAIP6	P98066
Gardeniae Fructus	MOL000511	ursolic acid	Transcription factor AP-1	JUN	P05412
Gardeniae Fructus	MOL000511	ursolic acid	Interleukin-6	IL6R	P05231
Gardeniae Fructus	MOL000511	ursolic acid	Cell division protein kinase 6	CDK6	Q00534
Gardeniae Fructus	MOL000511	ursolic acid	Caspase-3	CASP3	P42574
Gardeniae Fructus	MOL000511	ursolic acid	Cellular tumor antigen p53	TP53	P04637
Gardeniae Fructus	MOL000511	ursolic acid	Mitogen-activated protein kinase 8	MAPK8	P45983
Gardeniae Fructus	MOL000511	ursolic acid	Prostaglandin G/H synthase 2	PTGS2	P35354
Gardeniae Fructus	MOL000511	ursolic acid	NF-kappa-B inhibitor alpha	NFKBIA	P25963
Gardeniae Fructus	MOL000511	ursolic acid	Caspase-8	CASP8	Q14790
Gardeniae Fructus	MOL000511	ursolic acid	Fatty acid synthase	FASN	P49327
Gardeniae Fructus	MOL000511	ursolic acid	Interstitial collagenase	MMP1	P03956
Gardeniae Fructus	MOL000511	ursolic acid	Stromelysin-1	MMP3	P08254
Gardeniae Fructus	MOL000511	ursolic acid	Heparin-binding growth factor 2	FGF2	P09038
Gardeniae Fructus	MOL000511	ursolic acid	Stromelysin-2	MMP10	P09238
Gardeniae Fructus	MOL000511	ursolic acid	Intercellular adhesion molecule 1	ICAM1	P05362
Gardeniae Fructus	MOL000511	ursolic acid	Interleukin-1 beta	IL1B	P01584
Gardeniae Fructus	MOL000511	ursolic acid	Cyclic AMP-responsive element-binding protein 1	CREB1	P16220

Gardeniae Fructus	MOL000511	ursolic acid	E-selectin	SELE	P16581
Gardeniae Fructus	MOL000511	ursolic acid	Prostaglandin E2 receptor EP3 subtype	PTGER3	P43115
Gardeniae Fructus	MOL000511	ursolic acid	Prostaglandin G/H synthase 1	PTGS1	P23219
Gardeniae Fructus	MOL000511	ursolic acid	Induced myeloid leukemia cell differentiation protein Mcl-1	MCL1	Q07820
Gardeniae Fructus	MOL000511	ursolic acid	Protein kinase C gamma type	PRKCG	P05129
Gardeniae Fructus	MOL000511	ursolic acid	Cyclic AMP-dependent transcription factor ATF-2	ATF2	P15336
Gardeniae Fructus	MOL000511	ursolic acid	Granulocyte-macrophage colony-stimulating factor	CSF2	P04141
Gardeniae Fructus	MOL000511	ursolic acid	Platelet endothelial cell adhesion molecule	PECAM1	P16284
Gardeniae Fructus	MOL000511	ursolic acid	C-Jun-amino-terminal kinase-interacting protein 2	MAPK8IP2	Q13387
Gardeniae Fructus	MOL000511	ursolic acid	Baculoviral IAP repeat-containing protein 5	BIRC5	O15392
Gardeniae Fructus	MOL000511	ursolic acid	Tyrosine-protein phosphatase non-receptor type 6	PTPN6	P29350
Gardeniae Fructus	MOL000511	ursolic acid	Neuromodulin	GAP43	P17677
Gardeniae Fructus	MOL000511	ursolic acid	Dual oxidase 2	DUOX2	Q9NRD8
Gardeniae Fructus	MOL000511	ursolic acid	Nitric oxide synthase, endothelial	NOS3	P29474
Gardeniae Fructus	MOL000511	ursolic acid	Tyrosine-protein phosphatase non-receptor type 1	PTPN1	P18031
Gardeniae Fructus	MOL000511	ursolic acid	ipopolysaccharide-induced tumor necrosis factor-alpha factc	LITAF	Q99732
Gardeniae Fructus	MOL000511	ursolic acid	G1/S-specific cyclin-D2	CCND2	P30279
Gardeniae Fructus	MOL000511	ursolic acid	Tumor necrosis factor ligand superfamily member 6	FASLG	P48023
Gardeniae Fructus	MOL000511	ursolic acid	Caspase-1	CASP1	P29466
Gardeniae Fructus	MOL000511	ursolic acid	nucleotide pyrophosphatase/phosphodiesterase family memb	ENPP7	Q6UWV6
Gardeniae Fructus	MOL000003	MTL	Alcohol dehydrogenase 1C	ADH1C	P00326
Gardeniae Fructus	MOL000023	Hemo-sol	Prostaglandin G/H synthase 2	PTGS2	P35354
Gardeniae Fructus	MOL000023	Hemo-sol	Gamma-aminobutyric acid receptor subunit alpha-1	GABRA1	P14867
Gardeniae Fructus	MOL000023	Hemo-sol	Alcohol dehydrogenase 1B	ADH1B	P00325
Gardeniae Fructus	MOL000023	Hemo-sol	Alcohol dehydrogenase 1C	ADH1C	P00326
Gardeniae Fructus	MOL000023	Hemo-sol	Nuclear receptor coactivator 2	NCOA2	Q15596
Gardeniae Fructus	MOL000023	Hemo-sol	Nuclear receptor coactivator 1	NCOA1	Q15788
Gardeniae Fructus	MOL000023	Hemo-sol	Muscarinic acetylcholine receptor M2	CHRM2	P08172
Gardeniae Fructus	MOL000023	Hemo-sol	Muscarinic acetylcholine receptor M1	CHRM1	P11229
Gardeniae Fructus	MOL000023	Hemo-sol	Ig gamma-1 chain C region	IGHG1	P01857
Gardeniae Fructus	MOL000035	beta-Selinene	Prostaglandin G/H synthase 1	PTGS1	P23219
Gardeniae Fructus	MOL000035	beta-Selinene	Muscarinic acetylcholine receptor M3	CHRM3	P20309
Gardeniae Fructus	MOL000035	beta-Selinene	Muscarinic acetylcholine receptor M1	CHRM1	P11229
Gardeniae Fructus	MOL000035	beta-Selinene	Prostaglandin G/H synthase 2	PTGS2	P35354
Gardeniae Fructus	MOL000035	beta-Selinene	Retinoic acid receptor RXR-alpha	RXRA	P19793
Gardeniae Fructus	MOL000035	beta-Selinene	Sodium-dependent noradrenaline transporter	SLC6A2	P23975
Gardeniae Fructus	MOL000035	beta-Selinene	Muscarinic acetylcholine receptor M2	CHRM2	P08172
Gardeniae Fructus	MOL000035	beta-Selinene	Alpha-1B adrenergic receptor	ADRA1B	P35368
Gardeniae Fructus	MOL000035	beta-Selinene	Gamma-aminobutyric acid receptor subunit alpha-1	GABRA1	P14867

Gardeniae Fructus	MOL000035	beta-Selinene	Nuclear receptor coactivator 2	NCOA2	Q15596
Gardeniae Fructus	MOL000105	protocatechuic acid	Prostaglandin G/H synthase 1	PTGS1	P23219
Gardeniae Fructus	MOL000105	protocatechuic acid	Arachidonate 5-lipoxygenase	ALOX5	P09917
Gardeniae Fructus	MOL000105	protocatechuic acid	Prostaglandin G/H synthase 2	PTGS2	P35354
Gardeniae Fructus	MOL000105	protocatechuic acid	Amine oxidase [flavin-containing] B	MAOB	P27338
Gardeniae Fructus	MOL000105	protocatechuic acid	Lysozyme	LYZ	P61626
Gardeniae Fructus	MOL000105	protocatechuic acid	Alcohol dehydrogenase 1C	ADH1C	P00326
Gardeniae Fructus	MOL000105	protocatechuic acid	Trypsin-3	PRSS3	P35030
Gardeniae Fructus	MOL000105	protocatechuic acid	Protein kinase C alpha type	PRKCA	P17252
Gardeniae Fructus	MOL000105	protocatechuic acid	Protein kinase C beta type	PRKCB	P05771
Gardeniae Fructus	MOL000105	protocatechuic acid	Protein kinase C gamma type	PRKCG	P05129
Gardeniae Fructus	MOL000105	protocatechuic acid	Protein kinase C zeta type	PRKCZ	Q05513
