# Supplementary material for: Network Pharmacology-Based Analysis on the Potential Biological Mechanisms of Yinzhihuang Oral Liquid in Treating Neonatal Hyperbilirubinemia
Source: Evid Based Complement Alternat Med. 2022 Oct 5;2022:1672670. doi: 10.1155/2022/1672670 (PMC9556251; doi:10.1155/2022/1672670)
Supplement: Supplementary Materials — Table S1: active herbal ingredients in Scutellariae Radix. Table S2: active herbal ingredients in Lonicerae Japonicae Flos. Table S3: active herbal ingredients in Artemisiae Scopariae Herba. Table S4: active herbal ingredients in Gardeniae Fructus. Table S5: ingredients in Scutellariae Radix and corresponding targets. Table S6: ingredients in Lonicerae Japonicae Flos and corresponding targets. Table S7: ingredients in Artemisiae Scopariae Herba and corresponding targets. Table S8: ingredients in Gardeniae Fructus and corresponding targets. Table S9: compound-common target network of YZH and neonatal hyperbilirubinemia. Table S10: PPI network into Cytoscape for YZH and neonatal hyperbilirubinemia analysis (minimum required interaction score of 0.9). Table S11: Gene Ontology (GO) Biological Process analysis (p < 0.05). [file 1672670.f1.zip › Table S9.pdf]

Table S9 compound-common target network of YZH and neonatal hyperbilirubinemia

Node1	Node2	Net	MolName
MOL001300	ADH1A	target	PEL
MOL001300	MAOA	target	PEL
MOL000018	GABRA1	target	(+/-)-Isoborneol
MOL000018	PTGS2	target	(+/-)-Isoborneol
MOL000198	PTGS1	target	(R)-linalool
MOL000198	PTGS2	target	(R)-linalool
MOL000198	KDR	target	(R)-linalool
MOL000198	GABRA1	target	(R)-linalool
MOL000219	ADH1A	target	BOX
MOL000219	PTGS2	target	BOX
MOL000219	RXRA	target	BOX
MOL000219	GABRA1	target	BOX
MOL002573	PTGS2	target	β-patchoulene
MOL002573	GABRA1	target	β-patchoulene
MOL002714	PTGS1	target	baicalein
MOL002714	AR	target	baicalein
MOL002714	PTGS2	target	baicalein
MOL002714	DPP4	target	baicalein
MOL002714	PRSS1	target	baicalein
MOL002714	RELA	target	baicalein
MOL002714	AKT1	target	baicalein
MOL002714	VEGFA	target	baicalein
MOL002714	BCL2	target	baicalein
MOL002714	FOS	target	baicalein
MOL002714	BAX	target	baicalein
MOL002714	MMP9	target	baicalein
MOL002714	CASP3	target	baicalein
MOL002714	TP53	target	baicalein
MOL002714	HIF1A	target	baicalein
MOL002714	CDK1	target	baicalein
MOL002714	MPO	target	baicalein
MOL002714	AHR	target	baicalein
MOL002714	IGF2	target	baicalein
MOL002714	CYCS	target	baicalein
MOL002714	APOD	target	baicalein
MOL002737	PTGS1	target	scutellarein
MOL002737	AR	target	scutellarein
MOL002737	PTGS2	target	scutellarein
MOL002737	PRSS1	target	scutellarein
MOL002737	VEGFA	target	scutellarein
MOL002909	NOS2	target	5,7,2,5-tetrahydroxy-8,6-dimethoxyflavone
MOL002909	AR	target	5,7,2,5-tetrahydroxy-8,6-dimethoxyflavone
MOL002909	PTGS2	target	5,7,2,5-tetrahydroxy-8,6-dimethoxyflavone
MOL002909	DPP4	target	5,7,2,5-tetrahydroxy-8,6-dimethoxyflavone
MOL002909	PYGM	target	5,7,2,5-tetrahydroxy-8,6-dimethoxyflavone
MOL002909	PRSS1	target	5,7,2,5-tetrahydroxy-8,6-dimethoxyflavone
MOL002910	PTGS1	target	Carthamidin
MOL002910	PTGS2	target	Carthamidin
MOL002913	PTGS1	target	Dihydrobaicalin_qt
MOL002913	PTGS2	target	Dihydrobaicalin_qt
MOL002914	PTGS1	target	Eriodyctiol (flavanone)
MOL002914	PTGS2	target	Eriodyctiol (flavanone)
MOL002914	PYGM	target	Eriodyctiol (flavanone)
MOL002915	NOS2	target	Salvigenin
MOL002915	PTGS1	target	Salvigenin
MOL002915	PTGS2	target	Salvigenin
MOL002915	RXRA	target	Salvigenin
MOL002915	ADRB2	target	Salvigenin

MOL002915	DPP4	target	Salvigenin
MOL002915	PRSS1	target	Salvigenin
MOL002915	F7	target	Salvigenin
MOL002916	NOS2	target	2-(2,6-dihydroxyphenyl)-3,5,7-trihydroxy-chromone
MOL002916	PTGS1	target	2-(2,6-dihydroxyphenyl)-3,5,7-trihydroxy-chromone
MOL002916	AR	target	2-(2,6-dihydroxyphenyl)-3,5,7-trihydroxy-chromone
MOL002916	PPARG	target	2-(2,6-dihydroxyphenyl)-3,5,7-trihydroxy-chromone
MOL002916	PTGS2	target	2-(2,6-dihydroxyphenyl)-3,5,7-trihydroxy-chromone
MOL002917	NOS2	target	5,2',6'-Trihydroxy-7,8-dimethoxyflavone
MOL002917	PTGS1	target	5,2',6'-Trihydroxy-7,8-dimethoxyflavone
MOL002917	AR	target	5,2',6'-Trihydroxy-7,8-dimethoxyflavone
MOL002917	PTGS2	target	5,2',6'-Trihydroxy-7,8-dimethoxyflavone
MOL002917	ESR2	target	5,2',6'-Trihydroxy-7,8-dimethoxyflavone
MOL002917	DPP4	target	5,2',6'-Trihydroxy-7,8-dimethoxyflavone
MOL002917	CDK2	target	5,2',6'-Trihydroxy-7,8-dimethoxyflavone
MOL002917	PRSS1	target	5,2',6'-Trihydroxy-7,8-dimethoxyflavone
MOL002918	NOS2	target	Ganhuangenin
MOL002918	AR	target	Ganhuangenin
MOL002918	PTGS2	target	Ganhuangenin
MOL002918	F7	target	Ganhuangenin
MOL002918	ESR2	target	Ganhuangenin
MOL002918	DPP4	target	Ganhuangenin
MOL002918	PRSS1	target	Ganhuangenin
MOL002922	PTGS1	target	5-(2-hydroxyethyl)-2-methoxyphenol
MOL002922	PTGS2	target	5-(2-hydroxyethyl)-2-methoxyphenol
MOL002922	ADRB2	target	5-(2-hydroxyethyl)-2-methoxyphenol
MOL002922	MAOA	target	5-(2-hydroxyethyl)-2-methoxyphenol
MOL002924	NOS2	target	darendoside B_qt
MOL002924	ESR1	target	darendoside B_qt
MOL002924	PTGS2	target	darendoside B_qt
MOL002924	DPP4	target	darendoside B_qt
MOL002924	CDK2	target	darendoside B_qt
MOL002925	PTGS1	target	5,7,2',6'-Tetrahydroxyflavone
MOL002925	AR	target	5,7,2',6'-Tetrahydroxyflavone
MOL002925	PTGS2	target	5,7,2',6'-Tetrahydroxyflavone
MOL002925	DPP4	target	5,7,2',6'-Tetrahydroxyflavone
MOL002927	NOS2	target	Skullcapflavone II
MOL002927	PTGS1	target	Skullcapflavone II
MOL002927	AR	target	Skullcapflavone II
MOL002927	PTGS2	target	Skullcapflavone II
MOL002927	F7	target	Skullcapflavone II
MOL002927	KDR	target	Skullcapflavone II
MOL002927	DPP4	target	Skullcapflavone II
MOL002927	PRSS1	target	Skullcapflavone II
MOL002928	NOS2	target	oroxylin a
MOL002928	PTGS1	target	oroxylin a
MOL002928	AR	target	oroxylin a
MOL002928	PTGS2	target	oroxylin a
MOL002928	RXRA	target	oroxylin a
MOL002928	ADRB2	target	oroxylin a
MOL002928	DPP4	target	oroxylin a
MOL002928	PRSS1	target	oroxylin a
MOL002928	BCL2	target	oroxylin a
MOL002928	IL6R	target	oroxylin a
MOL002928	CASP3	target	oroxylin a
MOL002928	CDK1	target	oroxylin a
MOL002928	CYP1A2	target	oroxylin a
MOL002928	CYP2C9	target	oroxylin a
MOL002930	MAOA	target	Tyrosol
MOL002931	BCL2L1	target	scutellarin

MOL002931	CASP3	target	scutellarin
MOL002932	NOS2	target	Panicolin
MOL002932	PTGS1	target	Panicolin
MOL002932	AR	target	Panicolin
MOL002932	PTGS2	target	Panicolin
MOL002932	ESR2	target	Panicolin
MOL002932	DPP4	target	Panicolin
MOL002932	CDK2	target	Panicolin
MOL002932	PRSS1	target	Panicolin
MOL002933	NOS2	target	5,7,4'-Trihydroxy-8-methoxyflavone
MOL002933	PTGS1	target	5,7,4'-Trihydroxy-8-methoxyflavone
MOL002933	ESR1	target	5,7,4'-Trihydroxy-8-methoxyflavone
MOL002933	AR	target	5,7,4'-Trihydroxy-8-methoxyflavone
MOL002933	PPARG	target	5,7,4'-Trihydroxy-8-methoxyflavone
MOL002933	PTGS2	target	5,7,4'-Trihydroxy-8-methoxyflavone
MOL002933	DPP4	target	5,7,4'-Trihydroxy-8-methoxyflavone
MOL002933	PYGM	target	5,7,4'-Trihydroxy-8-methoxyflavone
MOL002933	MAPK14	target	5,7,4'-Trihydroxy-8-methoxyflavone
MOL002933	GSK3B	target	5,7,4'-Trihydroxy-8-methoxyflavone
MOL002933	CDK2	target	5,7,4'-Trihydroxy-8-methoxyflavone
MOL002933	PRSS1	target	5,7,4'-Trihydroxy-8-methoxyflavone
MOL002934	NOS2	target	NEOBAICALEIN
MOL002934	ESR1	target	NEOBAICALEIN
MOL002934	AR	target	NEOBAICALEIN
MOL002934	PPARG	target	NEOBAICALEIN
MOL002934	PTGS2	target	NEOBAICALEIN
MOL002934	F7	target	NEOBAICALEIN
MOL002934	ESR2	target	NEOBAICALEIN
MOL002934	DPP4	target	NEOBAICALEIN
MOL002934	PYGM	target	NEOBAICALEIN
MOL002934	GSK3B	target	NEOBAICALEIN
MOL002934	PRSS1	target	NEOBAICALEIN
MOL002936	NOS2	target	5,8-Dihydroxy-6,7-dimethoxyflavone
MOL002936	PTGS1	target	5,8-Dihydroxy-6,7-dimethoxyflavone
MOL002936	ESR1	target	5,8-Dihydroxy-6,7-dimethoxyflavone
MOL002936	AR	target	5,8-Dihydroxy-6,7-dimethoxyflavone
MOL002936	PPARG	target	5,8-Dihydroxy-6,7-dimethoxyflavone
MOL002936	PTGS2	target	5,8-Dihydroxy-6,7-dimethoxyflavone
MOL002936	F7	target	5,8-Dihydroxy-6,7-dimethoxyflavone
MOL002936	ESR2	target	5,8-Dihydroxy-6,7-dimethoxyflavone
MOL002936	DPP4	target	5,8-Dihydroxy-6,7-dimethoxyflavone
MOL002936	MAPK14	target	5,8-Dihydroxy-6,7-dimethoxyflavone
MOL002936	GSK3B	target	5,8-Dihydroxy-6,7-dimethoxyflavone
MOL002936	CDK2	target	5,8-Dihydroxy-6,7-dimethoxyflavone
MOL002936	PRSS1	target	5,8-Dihydroxy-6,7-dimethoxyflavone
MOL002937	PTGS1	target	DIHYDROOROXYLIN
MOL002937	PTGS2	target	DIHYDROOROXYLIN
MOL002937	RXRA	target	DIHYDROOROXYLIN
MOL002937	ADRB2	target	DIHYDROOROXYLIN
MOL000357	PTGS1	target	Sitogluside
MOL000357	PTGS2	target	Sitogluside
MOL000357	RXRA	target	Sitogluside
MOL000357	ADRB2	target	Sitogluside
MOL000358	PTGS1	target	beta-sitosterol
MOL000358	PTGS2	target	beta-sitosterol
MOL000358	ADRB2	target	beta-sitosterol
MOL000358	GABRA1	target	beta-sitosterol
MOL000358	BCL2	target	beta-sitosterol
MOL000358	BAX	target	beta-sitosterol
MOL000358	CASP9	target	beta-sitosterol

MOL000358	JUN	target	beta-sitosterol
MOL000358	CASP3	target	beta-sitosterol
MOL000358	CASP8	target	beta-sitosterol
MOL000358	PRKCA	target	beta-sitosterol
MOL000358	PON1	target	beta-sitosterol
MOL000359	NR3C2	target	sitosterol
MOL000396	PTGS2	target	(+)-Syringaresinol
MOL000525	NOS2	target	Norwogonin
MOL000525	PTGS1	target	Norwogonin
MOL000525	AR	target	Norwogonin
MOL000525	PPARG	target	Norwogonin
MOL000525	PTGS2	target	Norwogonin
MOL000525	DPP4	target	Norwogonin
MOL000525	CDK2	target	Norwogonin
MOL000552	NOS2	target	5,2'-Dihydroxy-6,7,8-trimethoxyflavone
MOL000552	PTGS1	target	5,2'-Dihydroxy-6,7,8-trimethoxyflavone
MOL000552	AR	target	5,2'-Dihydroxy-6,7,8-trimethoxyflavone
MOL000552	PTGS2	target	5,2'-Dihydroxy-6,7,8-trimethoxyflavone
MOL000552	F7	target	5,2'-Dihydroxy-6,7,8-trimethoxyflavone
MOL000552	ESR2	target	5,2'-Dihydroxy-6,7,8-trimethoxyflavone
MOL000552	DPP4	target	5,2'-Dihydroxy-6,7,8-trimethoxyflavone
MOL000552	PRSS1	target	5,2'-Dihydroxy-6,7,8-trimethoxyflavone
MOL000552	KDR	target	5,2'-Dihydroxy-6,7,8-trimethoxyflavone
MOL000116	GABRA1	target	Nonanal
MOL001168	PTGS1	target	(1S,2S)-2-isopropenyl-4-isopropylidene-1-methyl-1-vinylcyclohexane
MOL001168	PTGS2	target	(1S,2S)-2-isopropenyl-4-isopropylidene-1-methyl-1-vinylcyclohexane
MOL001168	RXRA	target	(1S,2S)-2-isopropenyl-4-isopropylidene-1-methyl-1-vinylcyclohexane
MOL001168	GABRA1	target	(1S,2S)-2-isopropenyl-4-isopropylidene-1-methyl-1-vinylcyclohexane
MOL001180	PTGS1	target	γ-muurolene
MOL001180	PTGS2	target	γ-muurolene
MOL001180	RXRA	target	γ-muurolene
MOL001180	GABRA1	target	γ-muurolene
MOL000123	CCND1	target	geraniol
MOL000123	MAPK3	target	geraniol
MOL000123	CDK4	target	geraniol
MOL000123	BAK1	target	geraniol
MOL000123	PRKCB	target	geraniol
MOL000123	HMGCR	target	geraniol
MOL000123	CYP2B6	target	geraniol
MOL000123	LCT	target	geraniol
MOL000125	GABRA1	target	(-)-alpha-Pinene
MOL001283	PTGS2	target	C09704
MOL001285	PRKCB	target	octanol
MOL001388	GABRA1	target	(+)-Ledol
MOL001393	PTGS1	target	myristic acid
MOL001393	PTGS2	target	myristic acid
MOL001393	LCAT	target	myristic acid
MOL000511	PLAU	target	ursolic acid
MOL000511	RELA	target	ursolic acid
MOL000511	STAT3	target	ursolic acid
MOL000511	VEGFA	target	ursolic acid
MOL000511	CCND1	target	ursolic acid
MOL000511	BCL2	target	ursolic acid
MOL000511	BCL2L1	target	ursolic acid
MOL000511	FOS	target	ursolic acid
MOL000511	CDKN1A	target	ursolic acid
MOL000511	BAX	target	ursolic acid
MOL000511	CASP9	target	ursolic acid
MOL000511	MMP2	target	ursolic acid
MOL000511	MMP9	target	ursolic acid

MOL000511	CDK4	target	ursolic acid
MOL000511	JUN	target	ursolic acid
MOL000511	IL6R	target	ursolic acid
MOL000511	CDK6	target	ursolic acid
MOL000511	CASP3	target	ursolic acid
MOL000511	TP53	target	ursolic acid
MOL000511	MAPK8	target	ursolic acid
MOL000511	PTGS2	target	ursolic acid
MOL000511	NFKBIA	target	ursolic acid
MOL000511	CASP8	target	ursolic acid
MOL000511	FASN	target	ursolic acid
MOL000511	MMP1	target	ursolic acid
MOL000511	MMP3	target	ursolic acid
MOL000511	FGF2	target	ursolic acid
MOL000511	ICAM1	target	ursolic acid
MOL000511	IL1B	target	ursolic acid
MOL000511	CREB1	target	ursolic acid
MOL000511	SELE	target	ursolic acid
MOL000511	PTGS1	target	ursolic acid
MOL000511	MCL1	target	ursolic acid
MOL000511	CSF2	target	ursolic acid
MOL000511	PECAM1	target	ursolic acid
MOL000511	BIRC5	target	ursolic acid
MOL000511	DUOX2	target	ursolic acid
MOL000511	NOS3	target	ursolic acid
MOL000511	FASLG	target	ursolic acid
MOL000511	CASP1	target	ursolic acid
MOL001494	PTGS1	target	Mandenol
MOL001494	PTGS2	target	Mandenol
MOL001495	PTGS1	target	Ethyl linolenate
MOL001600	PTGS2	target	copaene
MOL001600	GABRA1	target	copaene
MOL001600	RXRA	target	copaene
MOL001604	GABRA1	target	Linalool
MOL001719	GABRA1	target	2-[(2S,5R)-5-ethenyl-5-methyloxolan-2-yl]propan-2-ol
MOL000172	ADH1A	target	Furol
MOL001773	IFNG	target	indole
MOL001773	GCLC	target	indole
MOL000019	GABRA1	target	D-Camphene
MOL000019	PTGS2	target	D-Camphene
MOL000196	GABRA1	target	L-Bornyl acetate
MOL000196	GRIA2	target	L-Bornyl acetate
MOL000196	PTGS2	target	L-Bornyl acetate
MOL002042	PTGS1	target	thymol
MOL002042	PTGS2	target	thymol
MOL002042	ADRB2	target	thymol
MOL002042	ELANE	target	thymol
MOL000009	PRSS1	target	luteolin-7-o-glucoside
MOL000009	NOS2	target	luteolin-7-o-glucoside
MOL000009	PTGS2	target	luteolin-7-o-glucoside
MOL002083	NOS2	target	tricin
MOL002083	ESR1	target	tricin
MOL002083	AR	target	tricin
MOL002083	PPARG	target	tricin
MOL002083	PTGS2	target	tricin
MOL002083	F7	target	tricin
MOL002083	ESR2	target	tricin
MOL002083	DPP4	target	tricin
MOL002083	MAPK14	target	tricin
MOL002083	GSK3B	target	tricin

MOL002083	CDK2	target	tricin
MOL002083	PRSS1	target	tricin
MOL002083	PTGS1	target	tricin
MOL002085	PTGS2	target	alpha-Cubebene
MOL002085	GABRA1	target	alpha-Cubebene
MOL002121	PTGS1	target	(1S,4E,8E,10R)-4,8,11,11-tetramethylbicyclo[8.1.0]undeca-4,8-diene
MOL002121	PTGS2	target	(1S,4E,8E,10R)-4,8,11,11-tetramethylbicyclo[8.1.0]undeca-4,8-diene
MOL002121	GABRA1	target	(1S,4E,8E,10R)-4,8,11,11-tetramethylbicyclo[8.1.0]undeca-4,8-diene
MOL000252	PTGS2	target	farnesol
MOL000252	PTGS1	target	farnesol
MOL000252	RXRA	target	farnesol
MOL000252	IL6R	target	farnesol
MOL000252	CASP3	target	farnesol
MOL000252	BAK1	target	farnesol
MOL000252	TLR4	target	farnesol
MOL000252	LPL	target	farnesol
MOL000252	HMGCR	target	farnesol
MOL000252	PPARA	target	farnesol
MOL000252	TLR2	target	farnesol
MOL000252	NR1H4	target	farnesol
MOL000118	GABRA1	target	(L)-alpha-Terpineol
MOL000118	PTGS1	target	(L)-alpha-Terpineol
MOL000118	PTGS2	target	(L)-alpha-Terpineol
MOL001801	PTGS1	target	salicylic acid
MOL001801	PTGS2	target	salicylic acid
MOL001801	ALOX5	target	salicylic acid
MOL001801	ADH1A	target	salicylic acid
MOL001801	RELA	target	salicylic acid
MOL001801	FASN	target	salicylic acid
MOL001801	SOD1	target	salicylic acid
MOL001801	CAT	target	salicylic acid
MOL001801	EDNRA	target	salicylic acid
MOL001801	IL4	target	salicylic acid
MOL001801	GSTP1	target	salicylic acid
MOL001801	PON1	target	salicylic acid
MOL001801	APOA1	target	salicylic acid
MOL001801	PLG	target	salicylic acid
MOL001801	NFKB1	target	salicylic acid
MOL001801	IFNB1	target	salicylic acid
MOL001801	TPO	target	salicylic acid
MOL001801	GUSB	target	salicylic acid
MOL001801	HEY1	target	salicylic acid
MOL001801	TAT	target	salicylic acid
MOL001880	PTGS1	target	OXL
MOL001880	ALDH2	target	OXL
MOL001880	PTGS2	target	OXL
MOL001880	GRIN1	target	OXL
MOL001880	KYNU	target	OXL
MOL001880	GRIN2A	target	OXL
MOL001880	GABRA1	target	OXL
MOL001880	CTSD	target	OXL
MOL001880	LTF	target	OXL
MOL001880	MMP12	target	OXL
MOL001880	ADH1A	target	OXL
MOL001880	CAT	target	OXL
MOL001880	GLB1	target	OXL
MOL001880	MTR	target	OXL
MOL001880	TF	target	OXL
MOL001880	GLUD1	target	OXL
MOL001880	GPT	target	OXL

MOL001880	SDHA	target	OXL
MOL001880	PDHB	target	OXL
MOL001880	TPI1	target	OXL
MOL001880	AKR1B1	target	OXL
MOL001880	PYGM	target	OXL
MOL001880	NDUFS1	target	OXL
MOL001880	SLC25A13	target	OXL
MOL001999	PTGS1	target	scoparone
MOL001999	PTGS2	target	scoparone
MOL001999	ADRB2	target	scoparone
MOL001999	GABRA1	target	scoparone
MOL001999	GRIA2	target	scoparone
MOL001999	RELA	target	scoparone
MOL001999	NFKBIA	target	scoparone
MOL001999	CCL2	target	scoparone
MOL001999	CXCL8	target	scoparone
MOL001999	TYR	target	scoparone
MOL000207	PTGS1	target	Methyleugenol
MOL000207	PTGS2	target	Methyleugenol
MOL000207	ADRB2	target	Methyleugenol
MOL000207	PLAU	target	Methyleugenol
MOL000251	NOS2	target	Rhamnocitrin
MOL000251	PTGS1	target	Rhamnocitrin
MOL000251	ESR1	target	Rhamnocitrin
MOL000251	AR	target	Rhamnocitrin
MOL000251	PPARG	target	Rhamnocitrin
MOL000251	PTGS2	target	Rhamnocitrin
MOL000251	ESR2	target	Rhamnocitrin
MOL000251	DPP4	target	Rhamnocitrin
MOL000251	MAPK14	target	Rhamnocitrin
MOL000251	GSK3B	target	Rhamnocitrin
MOL000251	CDK2	target	Rhamnocitrin
MOL000251	PRSS1	target	Rhamnocitrin
MOL000251	AKR1B1	target	Rhamnocitrin
MOL000254	PTGS1	target	eugenol
MOL000254	PTGS2	target	eugenol
MOL000254	ADRB2	target	eugenol
MOL000254	MAOA	target	eugenol
MOL000254	PLAU	target	eugenol
MOL000254	RELA	target	eugenol
MOL000254	CYP1A1	target	eugenol
MOL000254	ALOX5	target	eugenol
MOL000254	AHR	target	eugenol
MOL000254	ABCC2	target	eugenol
MOL000254	MUC1	target	eugenol
MOL000339	PTGS1	target	Isoscopoletin
MOL000339	PTGS2	target	Isoscopoletin
MOL000339	ADRB2	target	Isoscopoletin
MOL000339	GRIA2	target	Isoscopoletin
MOL000354	NOS2	target	isorhamnetin
MOL000354	PTGS1	target	isorhamnetin
MOL000354	ESR1	target	isorhamnetin
MOL000354	AR	target	isorhamnetin
MOL000354	PPARG	target	isorhamnetin
MOL000354	PTGS2	target	isorhamnetin
MOL000354	ESR2	target	isorhamnetin
MOL000354	DPP4	target	isorhamnetin
MOL000354	MAPK14	target	isorhamnetin
MOL000354	GSK3B	target	isorhamnetin
MOL000354	CDK2	target	isorhamnetin

MOL000354	PRSS1	target	isorhamnetin
MOL000354	CCNA2	target	isorhamnetin
MOL000354	PYGM	target	isorhamnetin
MOL000354	AKR1B1	target	isorhamnetin
MOL000354	F7	target	isorhamnetin
MOL000354	GABRA1	target	isorhamnetin
MOL000354	GRIA2	target	isorhamnetin
MOL000354	RELA	target	isorhamnetin
MOL000040	PTGS1	target	Scopoletol
MOL000040	PTGS2	target	Scopoletol
MOL000040	ADRB2	target	Scopoletol
MOL000040	GRIA2	target	Scopoletol
MOL000415	RELA	target	rutin
MOL000415	IL6R	target	rutin
MOL000415	CASP3	target	rutin
MOL000415	POR	target	rutin
MOL000415	SOD1	target	rutin
MOL000415	CAT	target	rutin
MOL000415	IL1B	target	rutin
MOL000415	CXCL8	target	rutin
MOL000415	PRKCB	target	rutin
MOL000415	ALOX5	target	rutin
MOL000415	HMGCR	target	rutin
MOL000415	GSTP1	target	rutin
MOL000415	C5AR1	target	rutin
MOL000415	FCER2	target	rutin
MOL000415	ITGB2	target	rutin
MOL004609	NOS2	target	Areapillin
MOL004609	AR	target	Areapillin
MOL004609	PTGS2	target	Areapillin
MOL004609	F7	target	Areapillin
MOL004609	ESR2	target	Areapillin
MOL004609	DPP4	target	Areapillin
MOL004609	PRSS1	target	Areapillin
MOL004557	CA2	target	geniposide
MOL004557	BCL2	target	geniposide
MOL004557	HMOX1	target	geniposide
MOL004557	GLP1R	target	geniposide
MOL004557	GSTM1	target	geniposide
MOL004559	CA2	target	(1S,4aS,5R,7S,7aS)-5,7-dihydroxy-7-methyl-1-[(2S,3R,4S,5S,6R)-3,4,5-trihydroxy-6-(hydroxymethyl)oxan-2-yl]oxy-4a,5,6,7a-tetrahydro-1H-cyclopenta[d]pyran-4-carboxylic acid
MOL004559	DPP4	target	(1S,4aS,5R,7S,7aS)-5,7-dihydroxy-7-methyl-1-[(2S,3R,4S,5S,6R)-3,4,5-trihydroxy-6-(hydroxymethyl)oxan-2-yl]oxy-4a,5,6,7a-tetrahydro-1H-cyclopenta[d]pyran-4-carboxylic acid
MOL004560	PRSS1	target	SHANZHISIDE_qt
MOL004560	GRIA2	target	SHANZHISIDE_qt
MOL004561	ESR1	target	Sudan III
MOL004561	PTGS2	target	Sudan III
MOL004561	F7	target	Sudan III
MOL004561	ESR2	target	Sudan III
MOL004561	DPP4	target	Sudan III
MOL004561	MAPK14	target	Sudan III
MOL004561	GSK3B	target	Sudan III
MOL004561	CDK2	target	Sudan III
MOL004561	CCNA2	target	Sudan III
MOL000023	PTGS2	target	Hemo-sol
MOL000023	GABRA1	target	Hemo-sol
MOL000035	PTGS1	target	beta-Selinene
MOL000035	PTGS2	target	beta-Selinene
MOL000035	RXRA	target	beta-Selinene
MOL000035	GABRA1	target	beta-Selinene
MOL000105	PTGS1	target	protocatechuic acid
MOL000105	ALOX5	target	protocatechuic acid

MOL000105	PTGS2	target	protocatechuic acid
MOL000105	PRKCA	target	protocatechuic acid
MOL000105	PRKCB	target	protocatechuic acid
MOL000105	PRKCZ	target	protocatechuic acid
